# NGEF is a potential prognostic biomarker and could serve as an indicator for immunotherapy and chemotherapy in lung adenocarcinoma

**DOI:** 10.1186/s12890-024-03046-1

**Published:** 2024-05-19

**Authors:** Xin Chen, Tao Zhang, Yan-qiu He, Ti-wei Miao, Jie Yin, Qian Ding, Mei Yang, Fang-ying Chen, Hong-ping Zeng, Jie Liu, Qi Zhu

**Affiliations:** 1 Department of Integrated Traditional Chinese and Western Medicine, Zigong First People’s Hospital, Zigong, China; 2 Department of Intensive Care Unit, Chongqing General Hospital, University of Chinese Academy of Sciences, Chongqing, China; 3 School of Automation & Information Engineering, Sichuan university of Science & Engineering, Zigong, China; 4 Department of Tuberculosis, The Third People’s Hospital of Tibet Autonomous Region, Lhasa, China

**Keywords:** Neuronal guanine nucleotide exchange factor, Lung adenocarcinoma, Prognosis, Immune infiltration, Immunotherapy, Chemotherapy

## Abstract

**Background:**

Neuronal guanine nucleotide exchange factor (NGEF) plays a key role in several cancers; however, its role in lung adenocarcinoma (LUAD) remains unclear. The aim of this study was to evaluate the efficacy of NGEF as a prognostic biomarker and potential therapeutic target for LUAD.

**Methods:**

NGEF expression data for multiple cancers and LUAD were downloaded from multiple databases. The high- and low-NGEF expression groups were constructed based on median NGEF expression in LUAD samples, and then performed Kaplan–Meier survival analysis. Differentially expressed genes (DEGs) from the two NGEF expression groups were screened and applied to construct a protein-protein interaction network. The primary pathways were obtained using gene set enrichment analysis. The associations between NGEF expression and clinical characteristics, immune infiltration, immune checkpoint inhibitors (ICIs), sensitivity to chemotherapy, and tumor mutation burden (TMB) were investigated using R. Levels of NGEF expression in the lung tissue was validated using single-cell RNA sequencing, quantitative polymerase chain reaction (qPCR), immunohistochemical staining, and western blot analysis.

**Results:**

The expression of NGEF mRNA was upregulated in multiple cancers. mRNA and protein expression levels of NGEF were higher in patients with LUAD than in controls, as validated using qPCR and western blot. High NGEF expression was an independent prognostic factor for LUAD and was associated with advanced tumor stage, large tumor size, more lymph node metastasis, and worse overall survival (OS). A total of 182 overlapping DEGs were screened between The Cancer Genome Atlas and GSE31210, among which the top 20 hub genes were identified. NGEF expression was mainly enriched in the pathways of apoptosis, cell cycle, and DNA replication. Moreover, elevated NGEF expression were associated with a high fraction of activated memory CD4^+^ T cells and M_0_ macrophages; elevated expression levels of the ICIs: programmed cell death 1 and programmed cell death 1 ligand 1 expression; higher TMB; and better sensitivity to bortezomib, docetaxel, paclitaxel, and parthenolide, but less sensitivity to axitinib and metformin.

**Conclusion:**

NGEF expression is upregulated in LUAD and is significantly associated with tumor stages, OS probability, immune infiltration, immunotherapy response, and chemotherapy response. NGEF may be a potential diagnostic and prognostic biomarker and therapeutic target in LUAD.

**Supplementary Information:**

The online version contains supplementary material available at 10.1186/s12890-024-03046-1.

## Introduction

Lung cancer accounts for approximately 18% of the deaths caused by cancer worldwide [1], and its largest subgroup is lung adenocarcinoma (LUAD) [2]. LUAD is primarily located in distal lung and is difficult to diagnose through bronchoscopy, thereby posing a significant challenge on diagnosis [3]. Non-small cell lung cancer (NSCLC) is often not detected until they reach stages IIIB and IV; therefore, they have no opportunity for surgery [4]. Recently, a CT-guided thoracic core-needle biopsy shows an improved diagnostic accuracy for LUAD [5]. Although there has been a constant progress in disease diagnosis and targeted and immune therapies for LUAD patients over the past decades, the average 5-year overall survival (OS) probability maintains not more than 20% [6, 7]. Therefore, identifying effective biomarkers for predicting patient prognosis and/or therapeutic response is important for patients with LUAD.

Several biomarkers have been explored to diagnose LUAD and predict its prognosis. For example, the molecule CD1b (CD1B) is a novel prognostic biomarker in LUAD and related to its tumor mutation burden (TMB) as well as antitumor immunity [8]. Tumor protein D52-like 2 (TPD52L2) is an oncogene in LUAD, and associates with prognosis, and immune infiltration [9]. Mini-chromosome maintenance 5 (MCM5) expression is related to OS probability and clinical parameters, including TNM stage, pathological stage, and smoking status, in LUAD [10]. High eukaryotic initiation factor 3 (eIF3D) expression is independently associated with lower OS probability in LUAD [11]. Programmed death-ligand 1 (PD-L1) expression was positively correlated with the emergence of invasiveness and pathological subtype or biological behavior of early-stage lung adenocarcinoma [12]. However, these biomarkers are currently insufficient for the diagnosis and prognosis prediction of LUAD because of their complex molecular mechanisms and disease heterogenicity. For example, a joint analysis of transcriptome and proteome data shows that LUAD can be clustered into four novel subtypes with distinct molecular characteristics [13]. Therefore, new biomarkers need to be explored to better understand the complex molecular mechanisms, facilitate novel therapeutic methods, and improve the prognosis of LUAD patients.

Neuronal guanine nucleotide exchange factor (NGEF, also known as Ephexin1) is a member of a subfamily of the Dbl family of guanine nucleotide exchange factors (GEFs), and acts as a direct link between Eph receptors and the Rho-family of GTPases [14–16]. It was localized in mouse chromosome 1 and human chromosome 2q37 [16]. NGEF is mainly correlated with myopia and obesity-related diseases [17, 18]. However, several reports have demonstrated the involvement of NGEF in cancer. Wang et al. demonstrated that NGEF expression was upregulated in patients with malignant thyroid nodules; therefore, they reported NGEF as a potential diagnostic biomarker [19]. In addition, Han et al. showed that levels of NGEF expression were risen in patients with papillary thyroid cancer and were associated with a lower OS [20]. Thus, NGEF may act as an oncogene and may be associated with cancer prognosis. However, the diagnostic and prognostic importance of NGEF in patients with LUAD remains unclear. In this context, the current study explored the diagnostic and prognostic importance of NGEF in patients with LUAD.

In our study, NGEF expression, methylation, and gene mutation data for LUAD were comprehensively analyzed with the Gene Expression Profiling Interactive Analysis 2 (GEPIA2), The Cancer Genome Atlas (TCGA), and Gene Expression Omnibus (GEO) databases. The expression levels of NGEF mRNA were compared between LUAD and control samples. The results of bioinformatic analyses were validated by using quantitative polymerase chain reaction (qPCR), immunohistochemical (IHC) staining, single-cell RNA sequencing (scRNA-seq), and western blot analysis. The potential prognostic value of NGEF in LUAD was assessed using Kaplan–Meier survival curve, multivariable Cox regression analyses, and a nomogram. Differentially expressed genes (DEGs) from the two NGEF expression groups were screened and used to construct a protein-protein interaction (PPI) network. The primary pathways between the two NGEF expression groups were identified with gene set enrichment analysis. The R was used to evaluate the associations of NGEF expression with tumor stage, immune infiltration, immune checkpoint inhibitors (ICIs), TMB, and sensitivity to chemotherapy.

## Methods and materials

### Data collection and processing

The NGEF mRNA expression data for the most common cancers in China, such as LUAD (483 cancer tissues and 347 normal tissues), lung squamous cell carcinoma (LUSC, 486 cancer tissues and 338 normal tissues), stomach adenocarcinoma (STAD, 408 cancer tissues and 211 normal tissues), liver hepatocellular carcinoma (LIHC, 369 cancer tissues and 160 normal tissues), colon adenocarcinoma (COAD, 275 cancer tissues and 349 normal tissues), rectum adenocarcinoma (READ, 92 cancer tissues and 318 normal tissues), breast invasive carcinoma (BRCA, 1085 cancer tissues and 291 normal tissues), and thyroid carcinoma (THCA, 512 cancer tissues, and 337 normal tissues) were downloaded from the GEPIA2 database (http://gepia2.cancer-pku.cn/#index) [21, 22]. The mRNA expression data in the GEPIA2 database were acquired from TCGA and GTEx databases. In addition, 535 LUAD tissues and 59 normal tissues with mRNA expression data (FPKM format), 437 LUAD tissues and 29 normal tissues with methylation data, 560 LUAD tissues with mutation data, and 522 LUAD tissues with clinical characteristics data were downloaded from TCGA-GDC database (https://portal.gdc.cancer.gov/) [23, 24]. Additionally, 226 LUAD tissues and 20 normal tissues with mRNA expression data and 226 LUAD tissues with clinical characteristics data were obtained from the GEO database (https://www.ncbi.nlm.nih.gov/gds/) [25, 26]. GSE31210 dataset was generated by GPL570 [HG-U133_Plus_2] Affymetrix Human Genome U133 Plus 2.0 Array. Levels of NGEF expression were compared between LUAD samples and control samples in both TCGA and GSE31210 datasets. The ROC curve analysis was performed using GraphPad Prism (version 7.00), and the area under the curve (AUC) value, cut-off value, sensitivity, and specificity were calculated. Patients with LUAD who had complete follow-up information were enrolled for further analysis.

### Associations of NGEF expression levels with clinical characteristics

The high- or low-NGEF expression groups were constructed using all patients according to the median NGEF expression values obtained using R (version 4.0.2). Kaplan–Meier survival analysis was performed between the two NGEF expression groups and compared by the log-rank test using “limma” packages in R. The survival curve was plotted using “ggpubr” packages. Levels of NGEF expression were compared among different age groups, gender groups, Union for International Cancer Control (UICC) stages, T stages, N stages, and M stages. Univariate and multivariate Cox regression analyses were performed to screen independent prognostic factors for LUAD using “survival” packages in R and drew forest plot. *P*-value < 0.05 was seen as statistical significance, and the hazard ratio (HR) was estimated.

### Construction of nomogram and calibration plots

A nomogram can be constructed using multiple parameters, including clinical characteristics and RNA sequencing data [27, 28]; thus, nomograms can better reflect prognosis and guide individualized therapy. A nomogram was established including UICC stage, and NGEF values using the “survival,” “regplot,” and “rms” packages in R. Difference of actual and predicted OS probability at 1, 3, and 5 years was evaluated using calibration plots of the nomogram.

### Identification of DEGs

The DEGs between the two NGEF expression groups were identified in TCGA and GSE31210 using the “limma” package in R based on |log_2_ fold change (FC)| ≥ 0.5 and false discovery rate (FDR) < 0.05. Heatmaps were drawn in TCGA and GSE31210 using the “pheatmap” packages in R. The overlapping DEGs were screened between TCGA and GSE31210 database using “Venn” packages in R.

### Establishment of PPI network and identification of hub genes

The STRING (https://string-db.org, version 11.0) is an online database applied to establish a PPI network of genes based on the known and predicted targets [29]. The PPI network was generated by utilizing the overlapping DEGs in the STRING database and then imported into Cytoscape (version 3.7.1) to identify the top 20 hub genes.

### Gene set enrichment analysis (GSEA) of NGEF

GSEA can be applied to determine whether a previously defined set of genes show statistical significance between two different groups [30]. According to the median NGEF expression values in LUAD, the high- and low-NGEF expression groups (NGEF.cls) and mRNA expression values (NGEF.gct) were obtained using Strawberry Perl (version 5.34.1). GSEA (version 4.0.3) was obtained from GSEA online website (http://www.gsea-msigdb.org/gsea/index.jsp). Pathway analyses between the two NGEF expression groups were run using GSEA using “c2.cp.kegg.v7.5.1. symbols.gmt”, and the phenotype label was “H-versus-L” and the standard of permutations was 1,000. A nominal *P*-value of < 0.05 and FDR of < 0.05 was considered statistical significance.

### Methylation and tumor mutation burden analyses

Methylation data and matrix for LUAD were obtained using Strawberry Perl. The Mann-Whitney test was used to evaluate difference in methylation between the two NGEF-expression groups. The data for gene mutation were downloaded from TCGA database. Gene mutation frequencies in the different NGEF expression groups were estimated utilizing “maftools” packages in R. The correlations between NGEF mRNA expression levels and TMB were evaluated using the Mann-Whitney test and Spearman rank correlation analysis. Kaplan–Meier survival analysis was performed between the high-NGEF expression + high TMB and low-NGEF expression + low TMB groups and compared using log-rank test with R.

### Association of NGEF with immune infiltration and immunotherapy

Tumor micro-environment score was obtained by “limma” and “e1071” packages in R according to mRNA expression matrix of LUAD. Violin plot was applied to compare and display different immune infiltration profiles between the high- and low-NGEF expression groups using “vioplot” packages in R. In addition, the associations between immune infiltration levels and NGEF expression levels were evaluated using R. Comparisons were performed using Spearman rank correlation analysis, and *P*-value of < 0.05 and |r| > 0.1 were statistical significance. The mRNA levels of ICIs (PD1 and PDL1) were compared between the different NGEF groups, and correlations were evaluated using Spearman rank correlation analysis.

### Association of NGEF with sensitivity to chemotherapy

Six chemotherapy drugs, including bortezomib [31, 32], docetaxel [33, 34], paclitaxel [35, 36], parthenolide [37, 38], axitinib [39–41], and metformin [42, 43], had shown their antitumor effects in lung cancer. The sensitivity to chemotherapy was calculated and evaluated between the two NGEF-expression groups using “pRRophetic” packages in R, and the half inhibitory concentration (IC50) was viewed as a reference standard of drug sensitivity.

### RNA extraction and quantitative PCR validation

Lung tissues (30 controls, 30 LUAD) were obtained from Zigong First People’s Hospital. The E.Z.N.A. HP Total RNA Kit (Omega, GA, USA) was used to extract total RNA, and then synthesized cDNA using the PrimeScript™ RT Reagent Kit (Takara, Japan) according to the instructions. The iQ™ SYBR Green Supermix (Bio-Rad) was applied to perform qPCR following the protocol. Beta-actin Ct value (endogenous reference) was used to normalize the relative gene expression levels, using the 2^−ΔΔC t^ relative quantification method. The quantitative PCR primers used were as follows:

NGEF-forward: 5′-TCCTGGACAAGACTGACGAC‐3′.

NGEF-reverse: 5′-TCCATCTTGTGGACACGGAA‐3′.

Beta-actin-forward: 5′‐CCACGAAACTACCTTCAACTCC‐3′.

Beta-actin -reverse: 5′‐GTGATCTCCTTCTGCATCCTGT‐3′.

### Single-cell RNA sequencing analysis

Single-cell RNA sequencing data for lung cancer, NSCLC, and LUAD were obtained from the GEO database. The inclusion criteria for the datasets were: (1) human samples, (2) LUAD, and (3) different cell types. The raw data and phenotype information of the selective dataset were obtained from the above database. The relative expression levels of NGEF were compared among different cell types.

### Total protein extraction and western blot analysis

Protein phosphatase inhibitor cocktail (Applygen Technologies, China), phenylmethylsulfonyl fluoride (PMSF) (Beyotime Biotechnology, China), and radioimmunoprecipitation assay (RIPA) buffer (Beyotime Biotechnology, China) were applied to extract total protein from cancer and control tissues of LUAD, and then determined its concentration via the BCA Protein Assay Kit (Thermo, USA). The sodium dodecyl sulfate-polyacrylamide gel electrophoresis with 10% running gel was used to load protein and then transferred onto a polyvinylidene fluoride (PVDF) membrane (Merck, USA). The raw PVDF membrane have been cropped according to the molecular weight and the top and bottom two markers of NGEF and Beta-actin membranes were retained. The cropped PVDF membranes were then incubated with primary antibodies against NGEF (1:1000, Abcam, ab157593, UK) or Beta-actin (1:2000, Proteintech, China) overnight at 4℃ after blocking using 5% bovine serum albumin for 2 h. The PVDF membranes were washed with Tris-buffered saline (TBS-T) five times for five minutes, incubated with secondary antibody (1:5,000, boster, China) for 2 h, and washed again. Subsequently, NGEF protein expression levels were determined.

### Immunohistochemical (IHC) staining

IHC staining of NGEF in normal lung tissues and LUAD tissues was downloaded from the Human Protein Atlas database (https://www.proteinatlas.org/) [44].

### Statistical analysis

GraphPad Prism (version 7.00) and R were used to perform statistical analyses and drawing all the figures. According to the nonparametric distribution, levels of gene relative expression were displayed as median (interquartile range), and statistical analysis were performed using the Mann–Whitney test. A *P*-value of < 0.05 was considered statistical significance.

## Results

### Increased levels of NGEF expression in multiple cancers

**Fig. 1:**
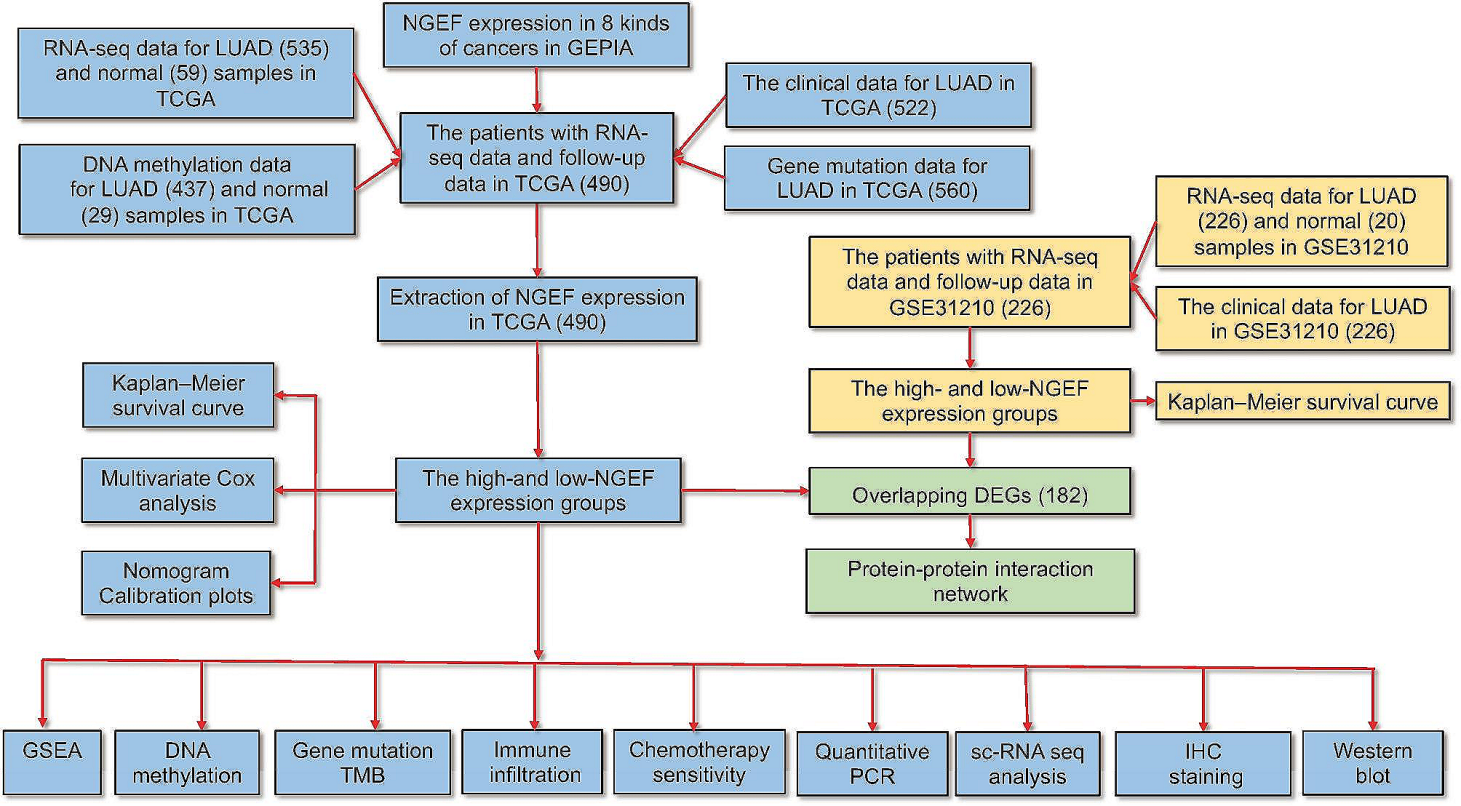
Study flowchart

**Fig. 2:**
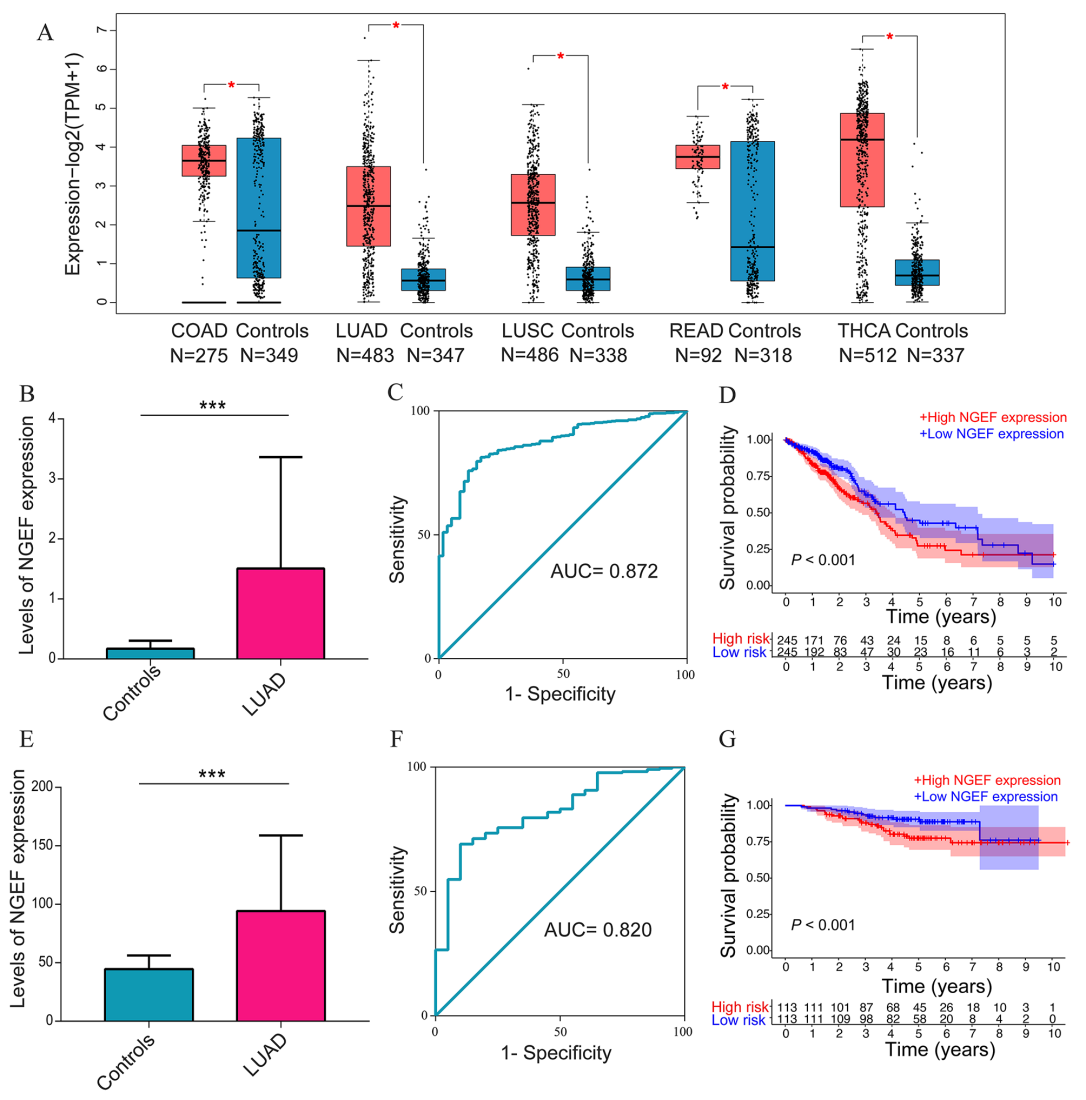
Diagnostic and prognostic value of NGEF. **(A)** Levels of NGEF expression in five tumor types in the GEPIA2. (**B**, **E**) Levels of NGEF expression in LUAD in TCGA and GSE31210. (**C**, **F**) Receiver operating characteristic curve analysis in TCGA and GSE31210. (**D**, **G**) Kaplan–Meier survival curve analysis in TCGA and GSE31210. NGEF, neuronal guanine nucleotide exchange factor; GEPIA2, Gene Expression Profiling Interactive Analysis 2; TCGA, The Cancer Genome Atlas, COAD, colon adenocarcinoma; LUAD, lung adenocarcinoma; LUSC, lung squamous cell carcinoma; READ, rectum adenocarcinoma; THCA, thyroid carcinoma; N, sample size. *P < 0.05, **P < 0.01, ***P < 0.001

A flowchart of this study is demonstrated in Fig. 1. The data from the GEPIA2 database reported that NGEF expression levels were upregulated in LUAD, LUSC, COAD, READ, and THCA (*P* < 0.05, Fig. 2A). Lung cancer is primary reason for the deaths caused by cancer [1]. LUAD is the largest subgroup of lung cancers [2]. Therefore, LUAD was selected for further analyses. 490 patients from TCGA and 226 patients from GSE31210 were included. Table 1 shows baseline information of all patients enrolled. The patients with LUAD presented an increase in levels of NGEF expression in comparison with controls in TCGA database (*P* < 0.001, Fig. 2B). The AUC value with 95% CIs for NGEF levels in the lungs for the diagnosis of LUAD was 0.872 (0.834–0.910), with a cut-off value of 0.3761, sensitivity of 81.31%, and specificity of 83.05% based on Youden’s index (Fig. 2C). Moreover, NGEF expression levels were risen in LUAD compared with controls in GSE31210 (*P* < 0.001, Fig. 2E), and its AUC value for the diagnosis of cancer was 0.820 (0.736–0.904) (Fig. 2F). Thus, NGEF is a diagnostic biomarker for LUAD.

**Table 1:** Clinical features of patients with LUAD from TCGA and GSE31210

Clinical characteristics	TCGA (490)	GSE31210 (226)
**Age (years)**		
>=65	219 (44.69%)	62 (27.43%)
< 65	271 (55.315)	164 (72.57%)
unknown	0	0
**Gender**		
Male	224 (45.71%)	105 (46.46%)
Female	266 (54.29%)	121 (53.54)
unknown	0	0
**UICC stages**		
Stage I-II	378 (77.14%)	226 (100%)
Stage III-IV	104 (21.22%)	/
unknown	8 (1.63%)	/
**T stages**		
T1	166 (33.88%)	/
T2-4	321 (66.51%)	/
unknown	3 (0.61%)	/
**N stages**		
N0	317 (64.69%)	/
N1-3	162 (33.06%)	/
unknown	11 (2.24%)	/
**M stages**		
M0	322 (65.71%)	/
M1	24 (4.90%)	/
unknown	144 (29.39%)	/

### Increased NGEF expression in LUAD was correlated with prognosis and clinical parameters

**Fig. 3:**
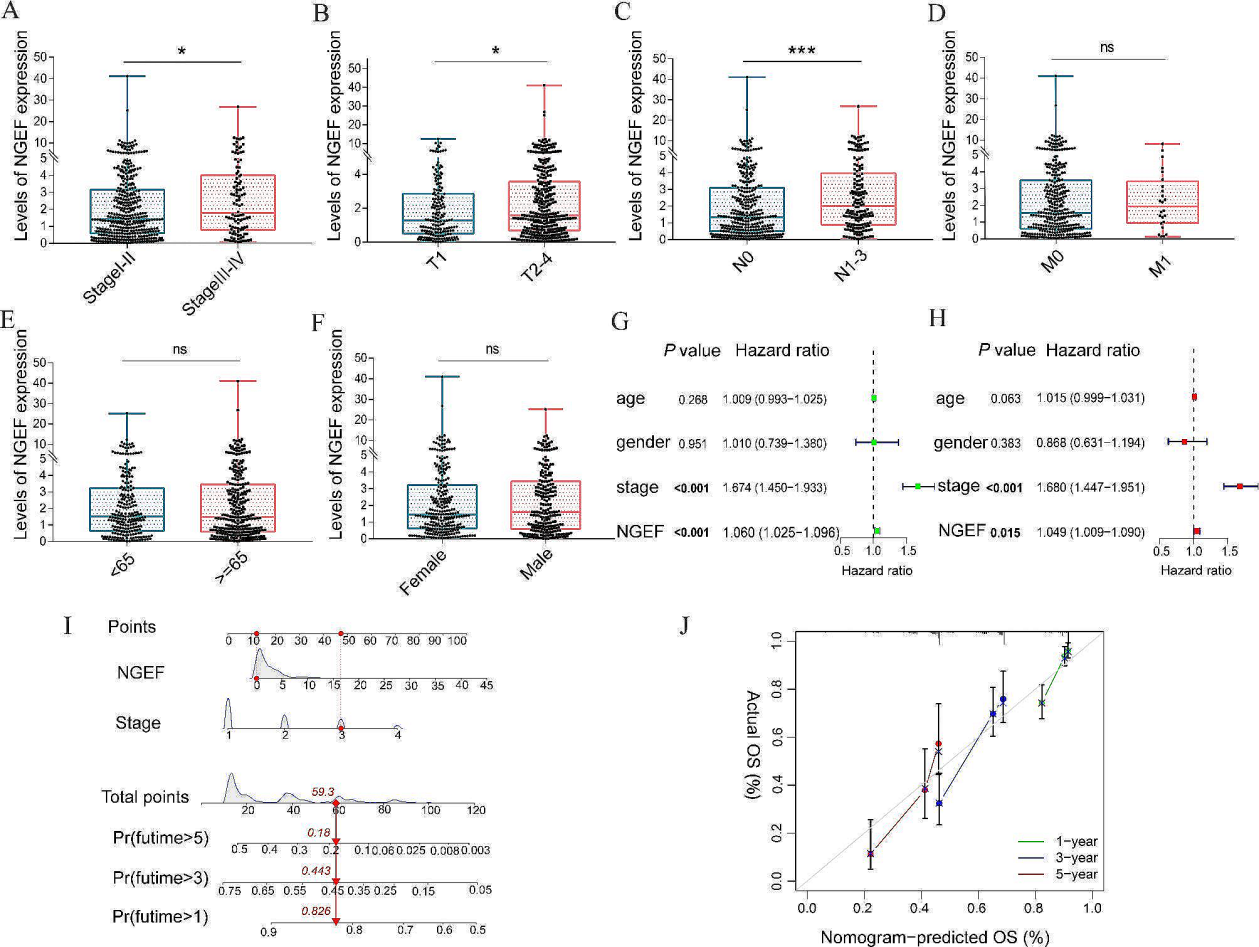
Correlations of NGEF with clinical characteristics. **(A)** UICC stages. **(B)** Tumor size (≤ 3 cm and > 3 cm). **(C)** Lymph node metastasis (0 and ≥ 1). **(D)** M stages. **(E)** Age groups. **(F)** Gender groups. (**G**, **H**) Univariate and multivariate Cox regression analyses for prognosis in LUAD. **(I)** Nomogram to predict OS at 1, 3, and 5 years. **(J)** Calibration plots of the nomogram. NGEF, neuronal guanine nucleotide exchange factor; UICC, Union for International Cancer Control; OS, overall survival; ns, no significance. *P < 0.05, **P < 0.01, ***P < 0.001

We next investigated the prognostic value of NGEF in LUAD. Kaplan–Meier survival curve demonstrated that the high NGEF expression levels experienced a shorter OS than the low NGEF expression levels in LUAD from TCGA database (*P* < 0.001, Fig. 2D) and GSE31210 datasets (*P* < 0.001, Fig. 2G). NGEF expression levels were positively correlated with UICC stage (*P* = 0.042, Fig. 3A), tumor size (> 3 cm, *P* = 0.020, Fig. 3B), and lymph node metastasis (number ≥ 1, *P* < 0.001, Fig. 3C). However, there was not a statistical significance in different M stages (*P* = 0.682), age groups (*P* = 0.887), or gender groups (*P* = 0.685) (Fig. 3D-F). The UICC stage (*P* < 0.001; hazard ratio, HR: 1.674) and NGEF (*P* < 0.001; HR: 1.060) were associated with prognosis in univariate Cox regression analysis (Fig. 3G). The UICC stage (*P* < 0.001, HR: 1.680) and NGEF (*P* = 0.015, HR: 1.049) were independently associated with prognosis in multivariate Cox regression analysis (Fig. 3H). Thus, the above findings show that NGEF is an independently prognostic biomarker for LUAD.

### Construction of nomogram and calibration plots

To further study prognostic value of NGEF in LUAD, a nomogram was drawn using the UICC stage and NGEF expression levels, and the estimated OS probability at 1, 3, and 5 years can be calculated by this nomogram (Fig. 3I). Calibration plots of the nomogram proved its high predictive accuracy (Fig. 3J).

### Identification of DEGs and hub genes

**Fig. 4:**
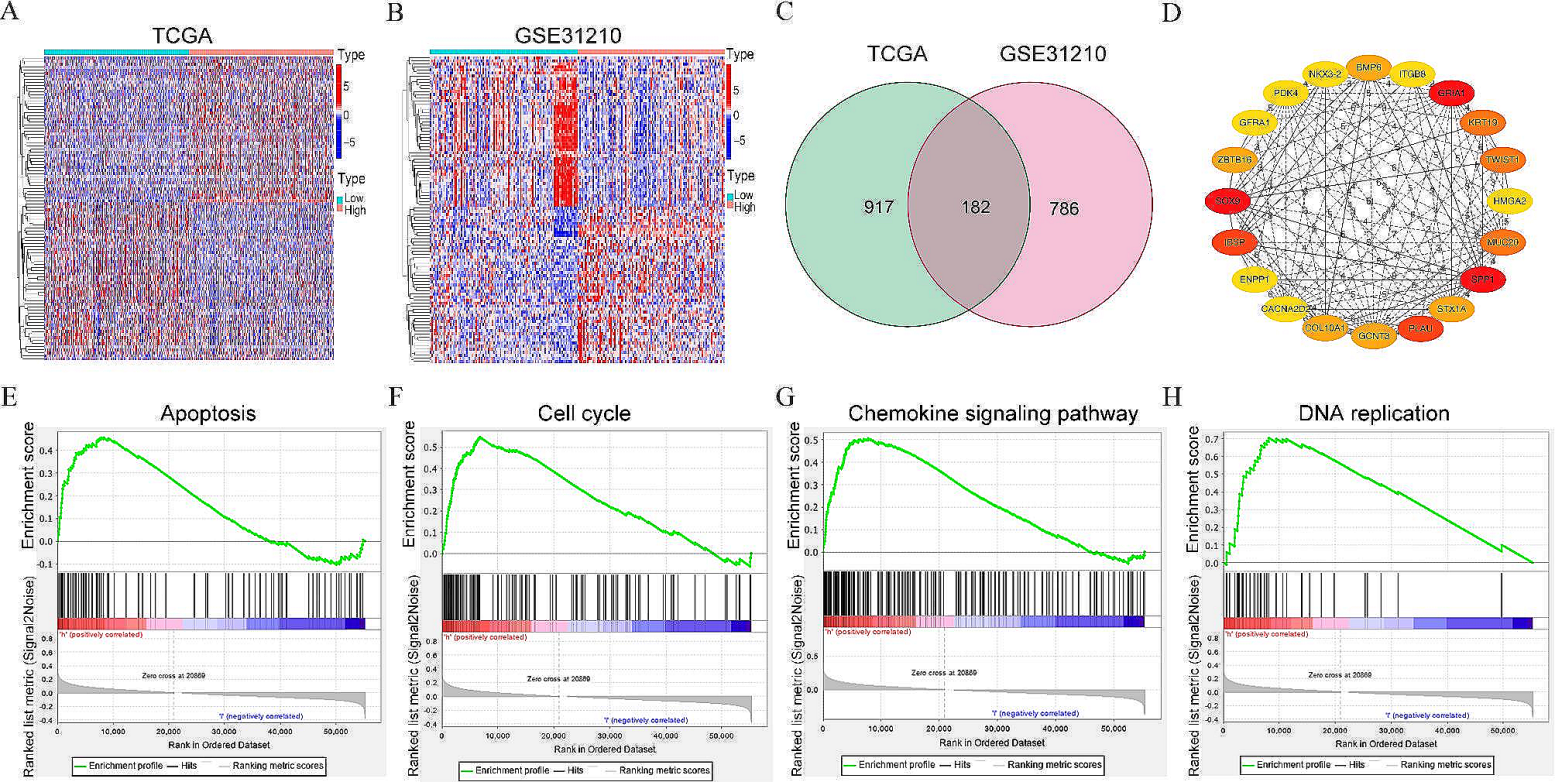
DEGs, hub genes, and GSEA. (**A**, **B**) DEGs between the high-NGEF expression and low-NGEF expression groups in LUAD in TCGA and GSE31210. **(C)** Overlapping DEGs between TCGA and GSE31210. **(D)** Top 20 hub genes. The red color represents the most significant genes, while the yellow color represents the less significant genes. **(E)** Apoptosis. **(F)** Cell cycle. **(G)** Chemokine signaling pathway. **(H)** DNA replication. DEGs, differentially expressed genes; NGEF, neuronal guanine nucleotide exchange factor; LUAD, lung adenocarcinoma; TCGA, The Cancer Genome Atlas; GSEA, gene set enrichment analysis

The differentially expressed analysis was performed, and a total of 1,099 DEGs (766 up-regulated and 333 down-regulated genes) were obtained using the “limma” packages in R when comparing the high-NGEF expression group with the low-NGEF expression group in TCGA (|log_2_ FC| ≥ 0.5 and FDR < 0.05, Table S1). Moreover, 968 DEGs (529 up-regulated and 439 down-regulated genes) were screened in GSE31210 (|log_2_ FC| ≥ 0.5 and FDR < 0.05, Table S2). Heatmaps created using TCGA and GSE31210 are illustrated in Fig. 4A and B, respectively. A total of 182 overlapping DEGs were screened between TCGA and GSE31210 datasets using the “Venn” packages (Fig. 4C). A PPI network was generated using 182 overlapping DEGs in the STRING database (Figure S1) and was visualized in the Cytoscape. The top 20 hub genes were screened using “Degree” in the Cytoscape (Fig. 4D, and Table 2), among which the top five genes were SPP1, SOX9, GRIA1, IBSP, and PLAU.

**Table 2:** Top 20 hub genes ranked by degree in Cytoscape software

Rank	Gene ID	Log_2_ FC (TCGA)	FDR (TCGA)
1	GRIA1	-0.998	0.005
1	SOX9	0.609	< 0.001
1	SPP1	0.763	< 0.001
4	IBSP	1.618	0.001
4	PLAU	1.093	< 0.001
6	TWIST1	0.599	< 0.001
6	MUC20	0.668	< 0.001
6	KRT19	0.563	< 0.001
9	GCNT3	1.047	< 0.001
9	BMP6	-1.398	< 0.001
9	COL10A1	0.576	0.023
9	STX1A	0.519	< 0.001
9	ZBTB16	-0.958	< 0.001
14	PDK4	-0.638	0.002
14	CACNA2D2	-1.431	< 0.001
14	NKX3-2	0.881	0.001
14	GFRA1	-0.871	0.004
14	ENPP1	0.608	< 0.001
14	HMGA2	1.135	< 0.001
14	ITGB8	0.995	< 0.001

### GSEA

To identify potential pathways between the two NGEF expression groups, GSEA was run. The findings showed that gene sets were primarily enriched in apoptosis, cell cycle, chemokine signaling pathway, and DNA replication in the high-NGEF expression group in LUAD (FDR < 0.05, nominal *P* < 0.05; Fig. 4E-H). Thus, we can infer that various pathways are enriched in the high-NGEF expression group.

### Increased NGEF expression was associated with higher TMB but not with methylation levels

**Fig. 5:**
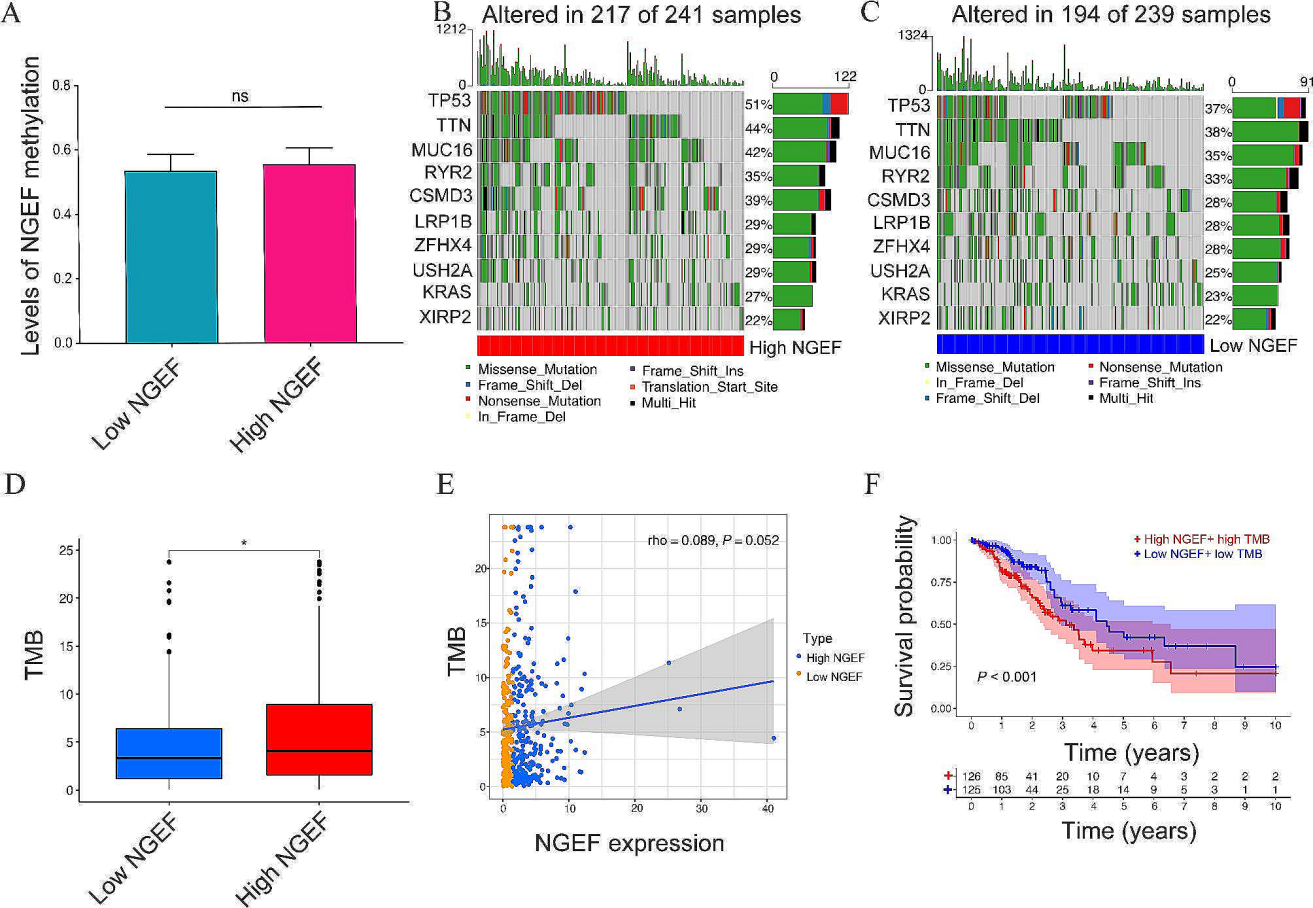
Methylation and mutation analyses. **(A)** Levels of NGEF methylation between the two NGEF expression groups. (**B**, **C**) Top 10 mutated genes in the high- and low-NGEF expression groups. (**D**, **E**) Correlation of NGEF expression with TMB. **(F)** Kaplan–Meier survival analysis between the high-NGEF expression + high TMB group and low-NGEF expression + low TMB group. NGEF, neuronal guanine nucleotide exchange factor; TMB, tumor mutation burden; ns, no significance. *P < 0.05

Since DNA methylation controls gene expression and thus influence prognosis and TMB is also correlated with prognosis, methylation levels and tumor mutation burden were analyzed between the two NGEF expression groups. Methylation levels of NGEF between the two groups did not reach a statistical significance (Fig. 5A). The high-NGEF expression group had higher gene mutation frequencies compared to low-NGEF expression group, and the top three mutated genes were TP53, TTN, and MUC16 in both NGEF expression groups (Fig. 5B-C). TMB was risen in the high-NGEF expression group compared with the low-NGEF expression group (*P* = 0.041, Fig. 5D), and the correlation of NGEF mRNA expression levels with TMB was at the border of a statistical difference (*P* = 0.052, rho = 0.089, Fig. 5E). Kaplan–Meier survival curve demonstrated that high-NGEF expression + high TMB group was correlated with a worse OS, compared to the low-NGEF expression + low TMB group (*P* < 0.001, Fig. 5F). The results show that the high-NGEF expression group had a higher TMB compared with the low-NGEF expression group and may thus lead to worse prognosis.

#### Increased NGEF expression was correlated with immune infiltration

**Fig. 6:**
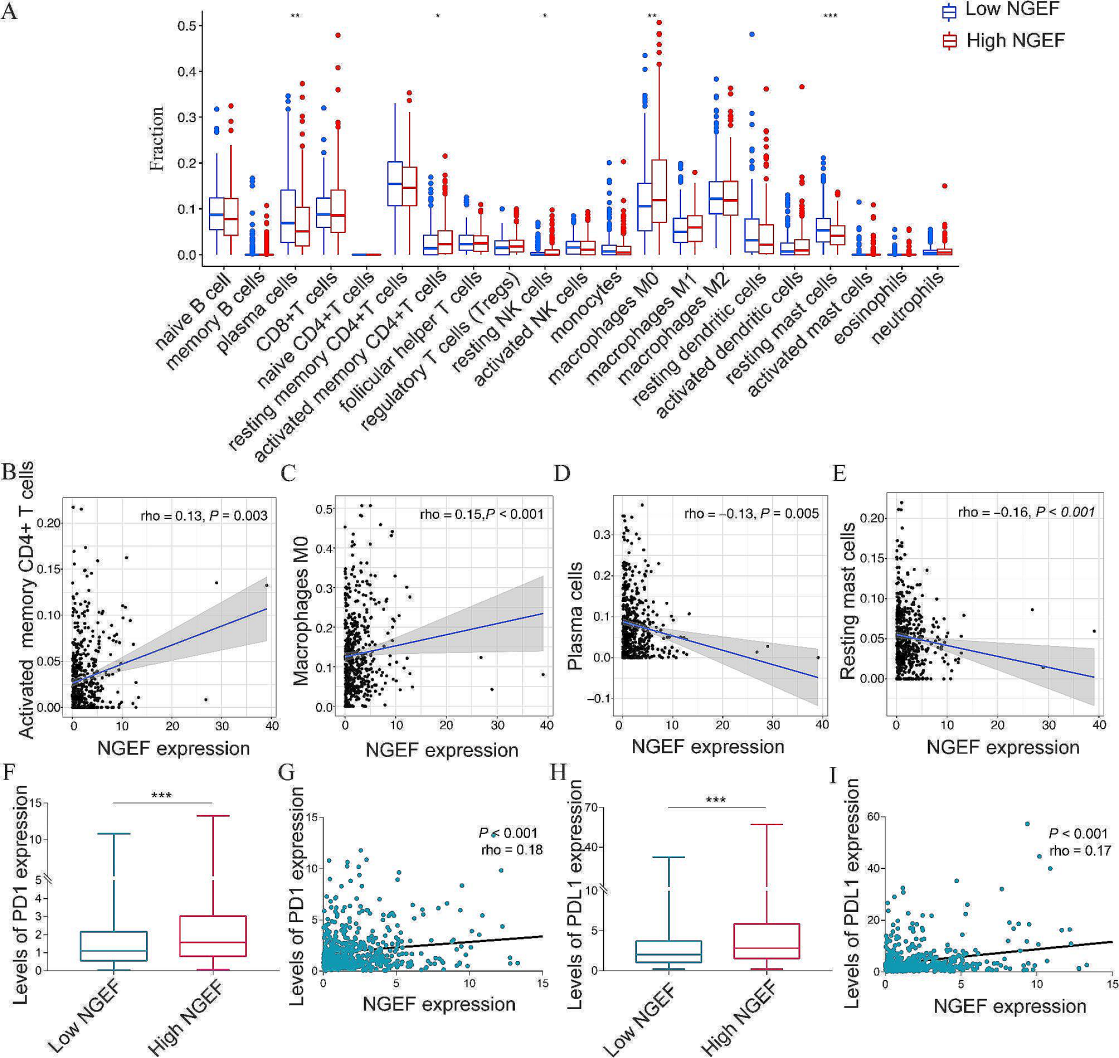
Correlations of NGEF expression with immune infiltration and immune checkpoint inhibitors. **(A)** Immune infiltration. **(B)** Activated memory CD4^+^ T cells. **(C)** M_0_ Macrophage. **(D)** Plasma cells. **(E)** Resting mast cells. (**F**, **G**) PD1. (**H**, **I**) PDL1. NGEF, neuronal guanine nucleotide exchange factor; PD1, programmed cell death 1; PDL1, programmed cell death 1 ligand 1. *P < 0.05, **P < 0.01, ***P < 0.001

We next investigated correlations of NGEF expression with immune infiltration. The fraction of activated memory CD4^+^ T cells (*P* < 0.05), resting NK cells (*P* < 0.05), and M_0_ macrophage (*P* < 0.01) in the high-NGEF expression group was higher than that in the low-NGEF expression group in LUAD (Fig. 6A). Furthermore, NGEF expression levels were positively associated with activated memory CD4^+^ T cells (*P* = 0.003, rho = 0.13) and M_0_ macrophage (*P* < 0.001, rho = 0.15) (Fig. 6B–C) and were negatively associated with plasma cells (*P* = 0.005, rho = -0.13) and resting mast cells (*P* < 0.001, rho = -0.16) (Fig. 6D–E). The correlation of NGEF expression levels with levels of ICI mRNA expression was evaluated, and the results indicated that levels of ICIs, including PD1 (*P* < 0.001) and PDL1 (*P* < 0.001), in the high-NGEF expression group showed an obvious growth, compared to the low-NGEF expression group (Fig. 6F, H). Moreover, increased NGEF expression was correlated with higher PD1 (*P* < 0.001, rho = 0.18) and PDL1 (*P* < 0.001, rho = 0.17) expression (Fig. 6G, I). Therefore, the high-NGEF expression group is correlated with a dysregulated immune infiltration and may be greater sensitivity to immunotherapy.

### Increased NGEF expression was correlated with chemotherapeutic sensitivity

**Fig. 7:**
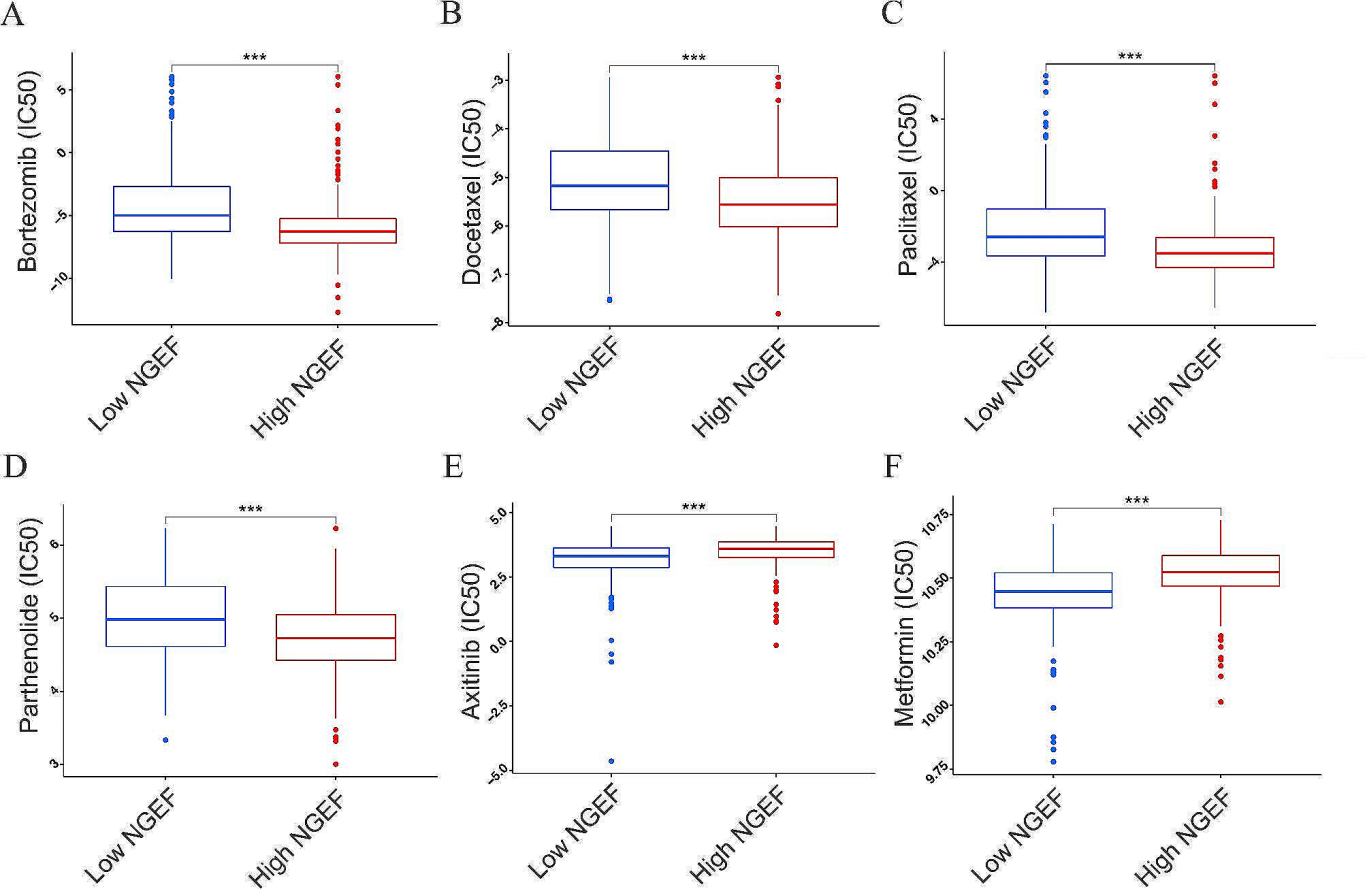
IC50 of six chemotherapeutic drugs. **(A)** Bortezomib. **(B)** Docetaxel. **(C)** Paclitaxel. **(D)** Parthenolide. **(E)** Axitinib. **(F)** Metformin. NGEF, neuronal guanine nucleotide exchange factor; IC50, half inhibitory concentration. ***P < 0.001

Chemotherapy still remains an important therapeutic approach for advanced LUAD because a part of cancer patients benefits from immunotherapy according to our results and previous researches [45, 46]. Therefore, correlations of NGEF expression with chemotherapy were analyzed. The results showed that the IC50 of bortezomib, docetaxel, paclitaxel, and parthenolide was lower in the high-NGEF expression group than in the low-NGEF expression group (*P* < 0.001, Fig. 7A-D), whereas the IC50 of metformin and axitinib was lower in the low-NGEF expression group (*P* < 0.001, Fig. 7E-F). Thus, NGEF expression may serve as reference for chemotherapeutic drug choice.

#### qPCR validation

**Fig. 8:**
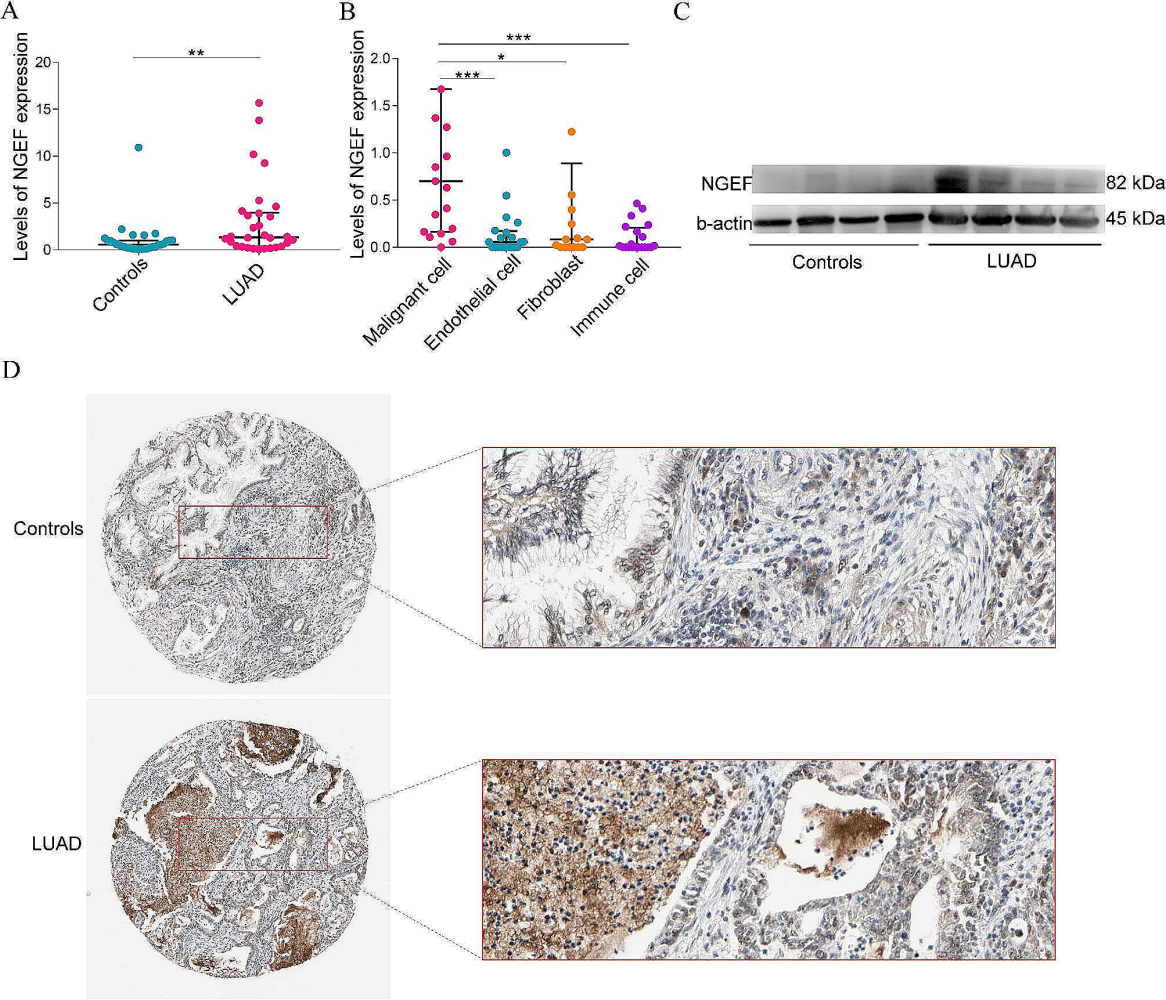
Validation of NGEF in LUAD. **(A)** Quantitative PCR validation of NGEF expression. **(B)** Single-cell RNA sequencing-based analysis of NGEF expression. **(C)** Western blot analysis of NGEF expression. **(D)**) Immunohistochemical data of NGEF from The Human Protein Atlas. . NGEF, neuronal guanine nucleotide exchange factor; LUAD, lung adenocarcinoma. *P < 0.05, **P < 0.01, ***P < 0.001

The baseline information of LUAD in our center is displayed in Table 3. To verify the results of bioinformatic analysis, qPCR was carried out using lung tissues. Results reported that the relative expression of NGEF was higher in LUAD than in controls (*P =* 0.007, Fig. 8A).

**Table 3:** Clinical characteristics of 30 paired lung tissues from patients with LUAD.

Clinical characteristics	patients (30)
Age, years	70 (65–73)
Male, n (%)	24 (80.00%)
Smoking status, *n* (%)	21 (70.00%)
UICC stage I-II, *n* (%)	11 (36.67%)

#### Single-cell RNA sequencing analysis

To investigate the source of increased NGEF expression, single-cell RNA sequencing analysis was performed. There were 76 datasets in GEO database when tailoring to “series” and “human sapiens,” among which only 10 datasets contained more than 100 different cell samples, including GSE176021, GSE111892, GSE151531, GSE151537, GSE156138, GSE69405, GSE127462, GSE167381, GSE111907, and GSE138734. The GSE111907 was the only dataset that met the inclusion criteria. Subsequently, 19 malignant cell samples, 20 endothelial cell samples, 17 fibroblast samples, and 20 immune cell samples from GSE111907 were included in this study. Levels of NGEF expression was significantly higher in malignant cell samples than in endothelial cell samples (*P* < 0.001), fibroblast samples (*P* = 0.013), and immune cell samples (*P* < 0.001). However, there was not a statistical significance among the endothelial cell, fibroblast, and immune cell samples (Fig. 8B).

#### Western blot and immunohistochemical staining

We next performed western blot and immunohistochemical staining to investigate the protein levels of NGEF between LUAD tissues and the corresponding control tissues. The results showed that the levels of NGEF-coded protein expression were higher in LUAD than in matching controls (Fig. 8C-D). Thus, we can further conclude that increased NGEF is a diagnostic biomarker for LUAD.

## Discussion

In LUAD, NGEF mRNA and protein expression levels were upregulated and correlated with advanced tumor stage and worse OS. NGEF is an independent prognostic indicator for LUAD. In addition, the increased levels of NGEF expression were related to a higher fraction of activated memory CD4^+^ T cells and M_0_ macrophage, elevated expression levels of ICIs, higher TMB, and better chemotherapeutic sensitivity (bortezomib, docetaxel, paclitaxel, and parthenolide).

Previous studies have demonstrated that NGEF expression is upregulated in malignant thyroid nodules [19], colorectal cancer [47] and papillary thyroid cancer [20]. Bioinformatic analyses demonstrated that NGEF expression was higher in multiple tumors. Thus, our study further confirmed that NGEF may act as an oncogene. However, the potential correlation between the NGEF and LUAD remains unclear. NGEF expression levels were upregulated in LUAD tissues in comparison with normal tissues in TCGA-LUAD and GSE31210 datasets. In addition, qPCR, IHC staining, and western blot analysis validated increased NGEF mRNA and protein levels in LUAD. Besides, AUC value of ROC curve exceeded 0.8 in both TCGA and GSE31210, showing a high diagnostic value. These findings indicate that NGEF could be a potential diagnostic biomarker for LUAD. The associations of NGEF with clinical characteristics were evaluated, and the results showed that NGEF expression levels were positively correlated with the tumor size and lymph node metastasis, further corroborating that NGEF expression might be related to the disease stage and degree of malignancy of LUAD. Kaplan-Meier survival curves showed that increased NGEF expression was associated with a shorter OS, which was consistent with the previous results in papillary thyroid cancer [20]. In addition, NGEF expression was independently associated with prognosis. The constructed nomogram could predict the OS probability at 1-, 3-, and 5-year in LUAD with a good predictive value. Therefore, NGEF may serve as a prognostic biomarker for patients with LUAD.

Interactions between epidermal growth factor receptor (EGFR) and EphA2 promote tumorigenesis through the action of Ephexin1 [48]. Besides, AKT-mediated Ephexin1-Ras interaction promotes oncogenic Ras signaling and cancer cell proliferation in colorectal and lung cancer [47]. However, whether these potential mechanisms are involved in LUAD deserves further investigation. GSEA showed that apoptosis, cell cycle, chemokine signaling, and DNA replication were the main pathways in the high-NGEF expression group of LUAD. Defects in apoptotic pathways foster malignant transformation of cells, tumor metastasis, and therapeutic resistance [49]. Besides, decrease in apoptotic activity contribute to tumorigenesis [50]. One of the crucial features on tumor is cell cycle dysregulation [51]. Disorder in cell cycle progression leads to unlimited proliferation and growth of tumor cells [52]. DNA replication may cause mutations [53], and gene mutations have enabled small cell lung cancer to be resistant to chemotherapy and have a lower OS probability [54]. Thus, NGEF may be related to the progression and prognosis of LUAD by activating these pathways. Disordered TMB showed its correlation with disease prognosis in cancer [55]. For example, TMB is a prognostic indicator for LUAD [56]. The high-NGEF expression group had a higher TMB, and the high-NGEF expression + high TMB group presented a shorter OS in the current study. Therefore, worse OS in the high-NGEF expression group may be associated with increased TMB.

In the tumor microenvironment, immune cells are crucial factors for tumor progression and response on all kinds of therapy [57]. Tumor-associated macrophages (TAMs) foster disease progression and immune escape via producing various inflammatory cytokines and chemokines [58]. Higher fractions of macrophages in cancer are associated with a worse OS [59]. The previous study has showed that TAMs are generally characterized by M2-like macrophages [60], which promote angiogenesis, invasion, metastasis, and resistance to therapy [61]. However, our findings only indicated a positive correlation of NGEF expression with M_0_ macrophage. Another study shows that knockdown of Circ_0001715 in M_0_ macrophages suppresses LUAD cell proliferation, migration and invasion [62]. Thus, M_0_ macrophages may play important role in cancer cell proliferation, migration and invasion. However, the potential mechanisms of M_0_ macrophages in LUAD need to be further investigated in future studies. ICIs have been proved its antitumor immunity [63, 64]. Clinically, atezolizumab and sintilimab showed improved OS, quality of life, and a favorable safety profile in NSCLC [65, 66]. Besides, nivolumab plus ipilimumab showed durable long-term efficacy in advanced NSCLC [67]. However, only a subset of patients with NSCLC can clinically benefit from it [68]. Selecting the right patient for a given therapy remains a critical unmet clinical need. Our results showed that NGEF expression was positively associated with ICIs (PD1 and PDL1) levels. Thus, these results reveal that high NGEF expression may be a useful indicator for response to immunotherapy. However, the patients with the low NGEF expression may benefit less from immunotherapy; thus, chemotherapeutic sensitivity was performed to screen proper chemotherapeutic drugs for the low-NGEF expression group. Our study showed that two chemotherapeutic drugs (axitinib and metformin) were more sensitive in the low-NGEF expression group. Besides, four chemotherapeutic drugs were more sensitive in the high-NGEF expression group. Cancer patients may benefit more from immunotherapy combined with chemotherapy compared with single immunotherapy or chemotherapy [69, 70], such as nivolumab plus ipilimumab with chemotherapy [71] and nivolumab plus chemotherapy [72, 73]. Thus, a better therapeutic strategy for high-NGEF expression group may be an immunotherapy combined with chemotherapy. Therefore, NGEF may serve as a reference for individualized therapy.

The present study highlights the following findings. First, our study reported that NGEF acts as an oncogene in several tumors and that NGEF is a diagnostic and prognostic biomarker for LUAD. Second, comprehensive and deep bioinformatic analysis was performed in the current study, including diagnostic and prognostic value; mechanism levels, such as methylation, mutation, GSEA, and immune infiltration; and therapeutic levels, such as immunotherapy and chemotherapy. Third, qPCR, IHC staining, scRNA-seq analysis, and western blot validated NGEF expression in the lung tissue. However, some limitations of our study must be noted. Although bioinformatic analyses revealed that NGEF expression was associated with immune infiltration and that apoptosis, cell cycle, chemokine signaling pathway, and DNA replication were the main pathways, further studies are warranted to investigate the specific role of NGEF in the tumor microenvironment and related pathways. Additionally, the mechanism by which NGEF is involved in tumor migration, invasion, and metastasis needs to be confirmed in vivo and in vitro.

## Conclusion

Using bioinformatic analysis, we systematically analyzed the expression patterns and prognostic and therapeutic value of NGEF in patients with LUAD from various databases. Our results indicate that the high NGEF expression has an advanced tumor stage and worse OS and that NGEF is an independent prognostic factor for LUAD. Moreover, increased NGEF expression was related to dysregulated immune infiltration, elevated ICI levels, higher TMB, and better sensitivity to four chemotherapeutic drugs (bortezomib, docetaxel, paclitaxel, and parthenolide). However, the low NGEF expression was more sensitive to two chemotherapeutic drugs. These findings reveal that NGEF may be a potential diagnostic and prognostic biomarker and therapeutic target for immunotherapy and chemotherapy in LUAD.

### Electronic supplementary material

Below is the link to the electronic supplementary material.

Supplementary Material 1

## Data Availability

The datasets used in this study are available from the GEPIA2 database (http://gepia2.cancer-pku.cn/#index), TCGA database (https://portal.gdc.cancer.gov/), GEO database (https://www.ncbi.nlm.nih.gov/gds/), the Human Protein Atlas database (https://www.proteinatlas.org/).
