# Supplementary material for: NGEF is a potential prognostic biomarker and could serve as an indicator for immunotherapy and chemotherapy in lung adenocarcinoma
Source: BMC Pulm Med. 2024 May 19;24:248. doi: 10.1186/s12890-024-03046-1 (PMC11102621; doi:10.1186/s12890-024-03046-1)
Supplement: Supplementary file 1 — Supplementary Material 1 [file 12890_2024_3046_MOESM1_ESM.docx]

**NGEF is a Potential Prognostic Biomarker and Could Serve as an Indicator for** **Immunotherapy and Chemotherapy in Lung Adenocarcinoma**

Running title: NGEF as a biomarker in LUAD

Xin Chen^1^*^#^, Tao Zhang^2^*, Yan-qiu He^1^*, Ti-wei Miao^1^, Jie Yin^3^, Qian Ding^1^, Mei Yang^1^, Fang-ying Chen^4^, Hong-ping Zeng^1^, Jie Liu^1^**,** Qi Zhu^1^

^1^Department of Integrated Traditional Chinese and Western Medicine, Zigong First People's Hospital, Zigong, China.

^2^Department of Intensive Care Unit, Chongqing General Hospital, University of Chinese Academy of Sciences, Chongqing, China.

^3^School of Automation & Information Engineering, Sichuan university of Science & Engineering, Zigong, China.

^4^Department of Tuberculosis, The Third People's Hospital of Tibet Autonomous Region, Lhasa, China.

Table S1. The DEGs between the high-NGEF expression group and the low-NGEF expression group in TCGA

| gene | lowMean | highMean | logFC | pValue | fdr |
| --- | --- | --- | --- | --- | --- |
| CGB5 | 0.089 | 3.981 | 5.476 | 0.000 | 0.000 |
| REG4 | 0.865 | 33.134 | 5.260 | 0.000 | 0.001 |
| TFF2 | 4.692 | 91.147 | 4.280 | 0.000 | 0.001 |
| PRSS33 | 0.026 | 0.361 | 3.769 | 0.009 | 0.028 |
| FER1L6 | 0.130 | 1.711 | 3.715 | 0.000 | 0.001 |
| NTSR1 | 0.099 | 1.158 | 3.551 | 0.000 | 0.000 |
| CEACAM7 | 0.720 | 8.252 | 3.518 | 0.000 | 0.001 |
| PCDH8 | 0.043 | 0.481 | 3.480 | 0.019 | 0.048 |
| CREB3L3 | 0.042 | 0.398 | 3.250 | 0.000 | 0.002 |
| ONECUT3 | 0.037 | 0.353 | 3.239 | 0.000 | 0.001 |
| ANXA10 | 1.255 | 11.444 | 3.189 | 0.000 | 0.001 |
| ALDOB | 0.288 | 2.475 | 3.104 | 0.000 | 0.000 |
| NMRK2 | 0.029 | 0.233 | 3.009 | 0.000 | 0.002 |
| CIDEC | 0.099 | 0.755 | 2.929 | 0.000 | 0.000 |
| HRG | 0.018 | 0.132 | 2.866 | 0.001 | 0.003 |
| PSCA | 7.047 | 49.848 | 2.822 | 0.000 | 0.000 |
| NGEF | 0.630 | 4.378 | 2.798 | 0.000 | 0.000 |
| CDHR2 | 0.243 | 1.653 | 2.764 | 0.000 | 0.000 |
| ANKRD40CL | 0.014 | 0.090 | 2.687 | 0.000 | 0.000 |
| CDHR5 | 0.306 | 1.878 | 2.616 | 0.000 | 0.002 |
| B3GALT5 | 0.141 | 0.856 | 2.601 | 0.000 | 0.000 |
| MIA | 0.315 | 1.851 | 2.555 | 0.000 | 0.000 |
| ASB11 | 0.058 | 0.335 | 2.537 | 0.009 | 0.026 |
| MYPN | 0.023 | 0.135 | 2.535 | 0.000 | 0.000 |
| MYO1A | 0.135 | 0.753 | 2.484 | 0.000 | 0.000 |
| MOGAT3 | 0.014 | 0.074 | 2.457 | 0.000 | 0.002 |
| PRR20G | 0.243 | 1.336 | 2.457 | 0.000 | 0.000 |
| NR1I2 | 0.083 | 0.452 | 2.453 | 0.000 | 0.000 |
| PRSS3 | 0.786 | 4.152 | 2.401 | 0.000 | 0.000 |
| UGT1A1 | 0.024 | 0.126 | 2.384 | 0.014 | 0.039 |
| GABRP | 1.242 | 6.475 | 2.382 | 0.001 | 0.004 |
| SERPINA4 | 0.522 | 2.712 | 2.377 | 0.000 | 0.001 |
| GDPD2 | 0.079 | 0.391 | 2.299 | 0.000 | 0.000 |
| SLC13A5 | 0.059 | 0.287 | 2.282 | 0.000 | 0.000 |
| APOBEC1 | 0.333 | 1.608 | 2.272 | 0.000 | 0.000 |
| SLC3A1 | 0.044 | 0.207 | 2.247 | 0.001 | 0.005 |
| TCN1 | 6.753 | 31.150 | 2.206 | 0.000 | 0.000 |
| CST4 | 0.626 | 2.844 | 2.185 | 0.000 | 0.000 |
| GJB4 | 0.162 | 0.710 | 2.136 | 0.000 | 0.000 |
| ODAPH | 0.064 | 0.278 | 2.110 | 0.000 | 0.000 |
| SERPINA6 | 0.151 | 0.650 | 2.107 | 0.002 | 0.008 |
| USH1C | 0.514 | 2.211 | 2.105 | 0.000 | 0.000 |
| LRRC66 | 0.158 | 0.659 | 2.062 | 0.000 | 0.000 |
| CYP3A5 | 0.663 | 2.765 | 2.060 | 0.000 | 0.000 |
| TINAG | 0.248 | 1.014 | 2.034 | 0.000 | 0.000 |
| SLC9A4 | 0.100 | 0.408 | 2.030 | 0.000 | 0.000 |
| TRIM10 | 0.049 | 0.195 | 1.995 | 0.000 | 0.000 |
| SLCO1B1 | 0.075 | 0.297 | 1.994 | 0.002 | 0.008 |
| GLP2R | 0.092 | 0.358 | 1.963 | 0.004 | 0.014 |
| EVX1 | 0.025 | 0.096 | 1.956 | 0.000 | 0.000 |
| HNF4A | 0.403 | 1.540 | 1.933 | 0.000 | 0.000 |
| AHSG | 0.031 | 0.120 | 1.929 | 0.000 | 0.000 |
| EPS8L3 | 0.931 | 3.532 | 1.924 | 0.000 | 0.000 |
| TRIM15 | 0.316 | 1.161 | 1.879 | 0.000 | 0.000 |
| PADI1 | 0.500 | 1.828 | 1.869 | 0.000 | 0.000 |
| DGKB | 0.028 | 0.101 | 1.865 | 0.003 | 0.012 |
| CREG2 | 0.114 | 0.411 | 1.853 | 0.000 | 0.000 |
| INSRR | 0.029 | 0.105 | 1.830 | 0.003 | 0.013 |
| CHST4 | 0.411 | 1.433 | 1.803 | 0.000 | 0.000 |
| SIX3 | 0.161 | 0.553 | 1.780 | 0.000 | 0.000 |
| TCHH | 0.133 | 0.454 | 1.773 | 0.000 | 0.000 |
| NPBWR1 | 0.121 | 0.409 | 1.763 | 0.000 | 0.000 |
| PDZD3 | 0.059 | 0.196 | 1.731 | 0.000 | 0.000 |
| LPO | 0.045 | 0.148 | 1.707 | 0.000 | 0.000 |
| SMIM24 | 0.939 | 3.007 | 1.679 | 0.019 | 0.048 |
| SERPINA5 | 0.570 | 1.817 | 1.674 | 0.000 | 0.000 |
| C8orf74 | 0.023 | 0.072 | 1.664 | 0.000 | 0.000 |
| HTR1D | 0.610 | 1.894 | 1.634 | 0.000 | 0.000 |
| CALHM3 | 0.059 | 0.183 | 1.629 | 0.000 | 0.000 |
| MAGEC3 | 0.070 | 0.216 | 1.629 | 0.002 | 0.009 |
| MAGEA10 | 0.760 | 2.348 | 1.628 | 0.000 | 0.000 |
| IBSP | 0.758 | 2.328 | 1.618 | 0.000 | 0.001 |
| EREG | 3.124 | 9.421 | 1.593 | 0.000 | 0.000 |
| MFAP5 | 1.200 | 3.615 | 1.591 | 0.000 | 0.000 |
| HLA-G | 4.310 | 12.921 | 1.584 | 0.000 | 0.000 |
| NIPAL4 | 0.159 | 0.473 | 1.574 | 0.000 | 0.000 |
| COL17A1 | 5.646 | 16.623 | 1.558 | 0.000 | 0.000 |
| SYT9 | 0.036 | 0.106 | 1.553 | 0.000 | 0.001 |
| DMRTC2 | 0.075 | 0.219 | 1.553 | 0.002 | 0.008 |
| F5 | 1.586 | 4.599 | 1.536 | 0.000 | 0.000 |
| SLC15A1 | 0.543 | 1.559 | 1.522 | 0.002 | 0.009 |
| CYP2C18 | 0.879 | 2.524 | 1.522 | 0.000 | 0.000 |
| IGFBP1 | 1.412 | 4.043 | 1.517 | 0.000 | 0.002 |
| UGT2B7 | 0.308 | 0.877 | 1.509 | 0.004 | 0.014 |
| KRT12 | 0.039 | 0.110 | 1.509 | 0.000 | 0.000 |
| OR51B5 | 0.059 | 0.168 | 1.508 | 0.017 | 0.044 |
| GJB3 | 2.929 | 8.247 | 1.494 | 0.000 | 0.000 |
| GDA | 0.268 | 0.755 | 1.493 | 0.000 | 0.000 |
| PRL | 0.051 | 0.141 | 1.472 | 0.018 | 0.047 |
| PNPLA1 | 0.054 | 0.147 | 1.445 | 0.000 | 0.000 |
| SLC22A11 | 0.025 | 0.067 | 1.442 | 0.000 | 0.000 |
| AKR1C4 | 0.645 | 1.750 | 1.439 | 0.002 | 0.007 |
| MT1A | 2.727 | 7.393 | 1.439 | 0.000 | 0.000 |
| RFLNA | 1.215 | 3.284 | 1.435 | 0.000 | 0.000 |
| ATP12A | 0.721 | 1.935 | 1.425 | 0.014 | 0.039 |
| MGAT5B | 0.199 | 0.534 | 1.421 | 0.000 | 0.000 |
| ZNF488 | 0.142 | 0.381 | 1.418 | 0.000 | 0.000 |
| FAM133A | 0.860 | 2.288 | 1.411 | 0.000 | 0.000 |
| CDA | 10.312 | 27.210 | 1.400 | 0.000 | 0.000 |
| CYP2C9 | 0.178 | 0.470 | 1.400 | 0.000 | 0.001 |
| IL37 | 7.114 | 18.761 | 1.399 | 0.004 | 0.015 |
| SH3TC2 | 0.037 | 0.098 | 1.397 | 0.000 | 0.000 |
| VNN1 | 1.688 | 4.437 | 1.394 | 0.000 | 0.000 |
| LRRC19 | 0.074 | 0.194 | 1.393 | 0.008 | 0.024 |
| MYH7 | 0.021 | 0.055 | 1.393 | 0.005 | 0.017 |
| GJA3 | 0.068 | 0.179 | 1.392 | 0.000 | 0.000 |
| VILL | 2.265 | 5.886 | 1.378 | 0.015 | 0.040 |
| ADH6 | 0.275 | 0.714 | 1.374 | 0.003 | 0.012 |
| KLK7 | 1.666 | 4.293 | 1.366 | 0.000 | 0.000 |
| IGFL3 | 0.121 | 0.311 | 1.365 | 0.000 | 0.000 |
| KLK6 | 2.824 | 7.265 | 1.363 | 0.000 | 0.000 |
| LAMC2 | 28.685 | 73.055 | 1.349 | 0.000 | 0.000 |
| TAF7L | 0.189 | 0.478 | 1.340 | 0.002 | 0.008 |
| UCN2 | 0.209 | 0.525 | 1.330 | 0.000 | 0.000 |
| KCP | 0.350 | 0.873 | 1.316 | 0.000 | 0.000 |
| HOXA11 | 0.159 | 0.395 | 1.316 | 0.000 | 0.000 |
| AXDND1 | 0.030 | 0.074 | 1.305 | 0.002 | 0.007 |
| GCKR | 0.212 | 0.522 | 1.301 | 0.000 | 0.000 |
| SCN5A | 0.058 | 0.143 | 1.299 | 0.000 | 0.000 |
| SBSN | 0.612 | 1.504 | 1.296 | 0.000 | 0.000 |
| GJB5 | 1.226 | 2.988 | 1.285 | 0.000 | 0.000 |
| CLCN1 | 0.055 | 0.134 | 1.285 | 0.000 | 0.000 |
| PADI3 | 0.928 | 2.257 | 1.282 | 0.000 | 0.000 |
| IL1R2 | 1.143 | 2.773 | 1.279 | 0.000 | 0.000 |
| TRPM8 | 0.841 | 2.039 | 1.277 | 0.000 | 0.001 |
| LGALS9B | 0.142 | 0.344 | 1.277 | 0.001 | 0.003 |
| IL20RB | 2.478 | 6.004 | 1.276 | 0.000 | 0.000 |
| FAM83B | 0.466 | 1.120 | 1.265 | 0.000 | 0.000 |
| ADGRF4 | 1.160 | 2.781 | 1.262 | 0.000 | 0.000 |
| HOXB13 | 0.537 | 1.287 | 1.260 | 0.000 | 0.000 |
| ACSM4 | 0.053 | 0.126 | 1.252 | 0.000 | 0.000 |
| TIMP4 | 0.383 | 0.911 | 1.251 | 0.000 | 0.000 |
| MYOM3 | 0.078 | 0.186 | 1.247 | 0.000 | 0.000 |
| MELTF | 2.020 | 4.789 | 1.245 | 0.000 | 0.000 |
| TMEM171 | 0.669 | 1.583 | 1.242 | 0.000 | 0.000 |
| PIWIL2 | 0.101 | 0.238 | 1.241 | 0.000 | 0.000 |
| PRR15 | 3.441 | 8.124 | 1.239 | 0.000 | 0.000 |
| KRT3 | 0.077 | 0.180 | 1.237 | 0.000 | 0.000 |
| G0S2 | 21.861 | 51.498 | 1.236 | 0.000 | 0.000 |
| SMIM32 | 0.065 | 0.151 | 1.230 | 0.001 | 0.006 |
| NDP | 0.505 | 1.183 | 1.228 | 0.000 | 0.000 |
| CACNG6 | 0.583 | 1.365 | 1.227 | 0.001 | 0.004 |
| FOSL1 | 4.465 | 10.434 | 1.224 | 0.000 | 0.000 |
| SYT14 | 0.081 | 0.190 | 1.222 | 0.000 | 0.001 |
| SERPINB5 | 2.673 | 6.218 | 1.218 | 0.000 | 0.000 |
| BCL2L10 | 0.390 | 0.899 | 1.203 | 0.000 | 0.000 |
| SLC5A1 | 1.317 | 3.018 | 1.196 | 0.001 | 0.003 |
| SUN3 | 0.402 | 0.920 | 1.196 | 0.000 | 0.000 |
| OR10H1 | 0.046 | 0.105 | 1.189 | 0.000 | 0.000 |
| TGM3 | 0.191 | 0.431 | 1.176 | 0.000 | 0.002 |
| OR2I1P | 5.652 | 12.761 | 1.175 | 0.000 | 0.000 |
| LYPD5 | 0.606 | 1.367 | 1.173 | 0.000 | 0.000 |
| SOX30 | 0.171 | 0.387 | 1.173 | 0.000 | 0.000 |
| ZNF280A | 0.069 | 0.156 | 1.172 | 0.000 | 0.000 |
| CYP4F22 | 0.135 | 0.303 | 1.164 | 0.010 | 0.030 |
| ACP7 | 0.083 | 0.186 | 1.159 | 0.000 | 0.000 |
| MMP11 | 13.362 | 29.697 | 1.152 | 0.000 | 0.000 |
| GPR87 | 4.192 | 9.314 | 1.152 | 0.000 | 0.000 |
| PNPLA5 | 0.087 | 0.193 | 1.151 | 0.000 | 0.001 |
| CRYBG2 | 0.910 | 2.013 | 1.146 | 0.000 | 0.000 |
| RP1L1 | 0.028 | 0.061 | 1.145 | 0.004 | 0.014 |
| GPR78 | 0.047 | 0.104 | 1.144 | 0.000 | 0.000 |
| FOXL1 | 0.561 | 1.238 | 1.143 | 0.005 | 0.018 |
| SNCG | 14.600 | 32.143 | 1.139 | 0.000 | 0.000 |
| CYP2W1 | 0.143 | 0.315 | 1.136 | 0.000 | 0.000 |
| HMGA2 | 0.799 | 1.755 | 1.135 | 0.000 | 0.000 |
| TH | 0.061 | 0.134 | 1.130 | 0.011 | 0.031 |
| ANO1 | 3.462 | 7.574 | 1.129 | 0.000 | 0.000 |
| KRTAP4-1 | 0.666 | 1.454 | 1.126 | 0.000 | 0.000 |
| TEX15 | 0.055 | 0.119 | 1.121 | 0.000 | 0.000 |
| APCDD1L | 0.403 | 0.877 | 1.120 | 0.000 | 0.001 |
| IL36RN | 0.649 | 1.408 | 1.117 | 0.000 | 0.001 |
| PRKCG | 0.087 | 0.188 | 1.117 | 0.000 | 0.000 |
| HAVCR1 | 0.886 | 1.916 | 1.113 | 0.000 | 0.000 |
| MAT1A | 0.567 | 1.225 | 1.111 | 0.000 | 0.000 |
| SLC9A2 | 0.349 | 0.753 | 1.109 | 0.000 | 0.002 |
| UPK1B | 3.847 | 8.267 | 1.104 | 0.000 | 0.002 |
| VSTM5 | 0.147 | 0.315 | 1.103 | 0.000 | 0.000 |
| TRIM31 | 2.119 | 4.548 | 1.102 | 0.000 | 0.000 |
| METTL11B | 0.064 | 0.137 | 1.098 | 0.001 | 0.004 |
| PLAU | 42.665 | 91.014 | 1.093 | 0.000 | 0.000 |
| RASGEF1C | 0.164 | 0.349 | 1.093 | 0.001 | 0.006 |
| SPOCD1 | 0.928 | 1.972 | 1.088 | 0.000 | 0.000 |
| JPH3 | 0.253 | 0.536 | 1.084 | 0.000 | 0.002 |
| OXTR | 0.539 | 1.142 | 1.082 | 0.000 | 0.000 |
| LYPD3 | 5.272 | 11.143 | 1.080 | 0.000 | 0.000 |
| MOG | 0.006 | 0.013 | 1.079 | 0.001 | 0.004 |
| SPTSSB | 1.064 | 2.248 | 1.079 | 0.000 | 0.000 |
| CCL1 | 0.130 | 0.275 | 1.078 | 0.000 | 0.000 |
| ADAMTS20 | 0.040 | 0.084 | 1.077 | 0.000 | 0.000 |
| SLC17A7 | 0.114 | 0.240 | 1.075 | 0.005 | 0.017 |
| MEGF10 | 0.097 | 0.204 | 1.073 | 0.000 | 0.002 |
| TGFBI | 28.378 | 59.569 | 1.070 | 0.000 | 0.000 |
| CYP4F12 | 0.319 | 0.665 | 1.060 | 0.003 | 0.012 |
| ATP10B | 1.039 | 2.153 | 1.051 | 0.000 | 0.001 |
| FUT6 | 1.199 | 2.480 | 1.048 | 0.000 | 0.001 |
| GCNT3 | 5.725 | 11.833 | 1.047 | 0.000 | 0.000 |
| TMPRSS11E | 4.490 | 9.260 | 1.044 | 0.000 | 0.000 |
| DIO3 | 0.246 | 0.508 | 1.044 | 0.001 | 0.005 |
| SDCBP2 | 6.389 | 13.172 | 1.044 | 0.000 | 0.000 |
| IL36G | 0.338 | 0.696 | 1.042 | 0.000 | 0.000 |
| RBP4 | 3.424 | 7.048 | 1.041 | 0.003 | 0.011 |
| RAD21L1 | 0.017 | 0.034 | 1.041 | 0.001 | 0.003 |
| BCO1 | 0.673 | 1.379 | 1.035 | 0.000 | 0.000 |
| P2RY4 | 0.129 | 0.264 | 1.031 | 0.007 | 0.021 |
| PDE11A | 0.056 | 0.114 | 1.031 | 0.001 | 0.006 |
| SYT5 | 0.204 | 0.416 | 1.029 | 0.000 | 0.000 |
| ATP8B3 | 0.517 | 1.055 | 1.028 | 0.000 | 0.000 |
| ZG16B | 1.935 | 3.945 | 1.028 | 0.000 | 0.000 |
| KCNK12 | 0.107 | 0.218 | 1.026 | 0.000 | 0.002 |
| GJB2 | 10.875 | 22.124 | 1.025 | 0.000 | 0.000 |
| C2orf72 | 1.027 | 2.089 | 1.024 | 0.000 | 0.000 |
| CALCR | 0.194 | 0.394 | 1.024 | 0.000 | 0.001 |
| CAMK2N1 | 10.401 | 21.110 | 1.021 | 0.000 | 0.000 |
| BAIAP2L2 | 2.269 | 4.602 | 1.021 | 0.000 | 0.000 |
| CD200R1L | 0.035 | 0.071 | 1.020 | 0.001 | 0.004 |
| DCBLD2 | 8.427 | 17.072 | 1.018 | 0.000 | 0.000 |
| DCHS2 | 0.067 | 0.135 | 1.017 | 0.003 | 0.011 |
| SLC2A14 | 0.102 | 0.207 | 1.017 | 0.000 | 0.000 |
| IFNL3 | 0.022 | 0.045 | 1.011 | 0.000 | 0.000 |
| SMPX | 0.268 | 0.539 | 1.010 | 0.000 | 0.000 |
| NEURL3 | 0.329 | 0.662 | 1.007 | 0.000 | 0.000 |
| POPDC3 | 0.850 | 1.706 | 1.005 | 0.000 | 0.000 |
| COX6B2 | 0.170 | 0.341 | 1.001 | 0.000 | 0.000 |
| RARRES1 | 12.609 | 25.214 | 1.000 | 0.000 | 0.002 |
| AGMO | 0.206 | 0.412 | 0.997 | 0.006 | 0.021 |
| HOXA10 | 0.694 | 1.385 | 0.996 | 0.000 | 0.000 |
| ITGB8 | 1.948 | 3.882 | 0.995 | 0.000 | 0.000 |
| KCNA7 | 0.024 | 0.047 | 0.995 | 0.000 | 0.001 |
| ABCC2 | 2.469 | 4.910 | 0.992 | 0.015 | 0.040 |
| TM4SF19 | 0.510 | 1.014 | 0.991 | 0.000 | 0.000 |
| CXCL5 | 5.869 | 11.656 | 0.990 | 0.001 | 0.003 |
| C19orf84 | 0.177 | 0.350 | 0.986 | 0.000 | 0.000 |
| POF1B | 2.635 | 5.205 | 0.982 | 0.000 | 0.000 |
| SLC6A20 | 0.989 | 1.952 | 0.982 | 0.000 | 0.001 |
| ZNF648 | 0.029 | 0.058 | 0.980 | 0.001 | 0.005 |
| BECN2 | 0.059 | 0.117 | 0.976 | 0.000 | 0.000 |
| GATM | 2.446 | 4.812 | 0.976 | 0.000 | 0.000 |
| BTG4 | 0.056 | 0.110 | 0.975 | 0.000 | 0.000 |
| KRTAP2-3 | 0.112 | 0.220 | 0.975 | 0.000 | 0.000 |
| MUC12 | 0.061 | 0.120 | 0.973 | 0.000 | 0.000 |
| GABRA3 | 0.484 | 0.949 | 0.972 | 0.008 | 0.024 |
| DMBX1 | 0.438 | 0.857 | 0.969 | 0.000 | 0.000 |
| VWDE | 0.480 | 0.940 | 0.969 | 0.000 | 0.000 |
| CCL26 | 0.580 | 1.135 | 0.967 | 0.000 | 0.000 |
| LYPD8 | 0.200 | 0.391 | 0.964 | 0.008 | 0.025 |
| DHRS9 | 2.376 | 4.618 | 0.959 | 0.000 | 0.000 |
| KCNF1 | 0.435 | 0.846 | 0.959 | 0.000 | 0.003 |
| MAEL | 0.775 | 1.506 | 0.958 | 0.002 | 0.006 |
| CNNM1 | 0.262 | 0.509 | 0.955 | 0.000 | 0.000 |
| SPOCK1 | 1.865 | 3.615 | 0.955 | 0.000 | 0.000 |
| ALOXE3 | 0.125 | 0.241 | 0.955 | 0.000 | 0.000 |
| SERPINB2 | 0.306 | 0.594 | 0.954 | 0.005 | 0.016 |
| POM121L2 | 0.025 | 0.048 | 0.954 | 0.000 | 0.001 |
| AIM2 | 3.805 | 7.365 | 0.953 | 0.000 | 0.000 |
| SBK3 | 0.210 | 0.405 | 0.950 | 0.000 | 0.000 |
| NETO1 | 0.195 | 0.376 | 0.948 | 0.000 | 0.000 |
| CEACAM5 | 160.789 | 309.934 | 0.947 | 0.001 | 0.003 |
| TRIM7 | 0.286 | 0.551 | 0.945 | 0.000 | 0.000 |
| IGSF23 | 0.072 | 0.137 | 0.938 | 0.005 | 0.016 |
| CD109 | 2.991 | 5.729 | 0.938 | 0.000 | 0.000 |
| FIBCD1 | 0.617 | 1.180 | 0.935 | 0.000 | 0.000 |
| CNTN2 | 0.019 | 0.036 | 0.932 | 0.003 | 0.010 |
| DAW1 | 0.772 | 1.470 | 0.929 | 0.000 | 0.001 |
| CHRNA7 | 0.034 | 0.064 | 0.929 | 0.000 | 0.000 |
| PPP1R14D | 12.046 | 22.774 | 0.919 | 0.003 | 0.010 |
| FAM71D | 0.051 | 0.095 | 0.915 | 0.001 | 0.003 |
| NUTM1 | 0.013 | 0.024 | 0.911 | 0.000 | 0.002 |
| ARNTL2 | 3.810 | 7.146 | 0.907 | 0.000 | 0.000 |
| CCL7 | 0.939 | 1.756 | 0.903 | 0.000 | 0.000 |
| SYT8 | 2.132 | 3.982 | 0.901 | 0.000 | 0.000 |
| MT3 | 0.214 | 0.399 | 0.899 | 0.005 | 0.016 |
| NCCRP1 | 3.115 | 5.791 | 0.895 | 0.000 | 0.000 |
| DOK5 | 0.999 | 1.855 | 0.893 | 0.000 | 0.000 |
| C17orf99 | 0.032 | 0.059 | 0.891 | 0.001 | 0.004 |
| UPK3A | 1.181 | 2.191 | 0.891 | 0.000 | 0.000 |
| RNF103-CHMP3 | 0.062 | 0.114 | 0.886 | 0.000 | 0.000 |
| XIRP1 | 0.162 | 0.300 | 0.885 | 0.000 | 0.000 |
| GRM1 | 0.021 | 0.040 | 0.884 | 0.004 | 0.014 |
| FLNC | 2.419 | 4.457 | 0.882 | 0.000 | 0.000 |
| NKX3-2 | 0.116 | 0.214 | 0.881 | 0.000 | 0.001 |
| ANKS4B | 0.614 | 1.130 | 0.880 | 0.001 | 0.004 |
| KLHL34 | 0.049 | 0.089 | 0.880 | 0.000 | 0.000 |
| STEAP1 | 11.016 | 20.262 | 0.879 | 0.000 | 0.000 |
| MYO18B | 0.077 | 0.142 | 0.877 | 0.001 | 0.004 |
| SRPX2 | 9.349 | 17.147 | 0.875 | 0.000 | 0.000 |
| SLC24A2 | 0.087 | 0.159 | 0.874 | 0.000 | 0.001 |
| PLAC8 | 3.998 | 7.313 | 0.871 | 0.003 | 0.010 |
| GAP43 | 0.284 | 0.520 | 0.870 | 0.000 | 0.000 |
| OASL | 3.073 | 5.608 | 0.868 | 0.000 | 0.000 |
| UBD | 9.721 | 17.732 | 0.867 | 0.000 | 0.000 |
| CCL15 | 0.250 | 0.455 | 0.864 | 0.002 | 0.008 |
| GPR158 | 0.219 | 0.399 | 0.863 | 0.001 | 0.003 |
| FA2H | 6.327 | 11.497 | 0.862 | 0.000 | 0.000 |
| NPFFR2 | 0.278 | 0.505 | 0.861 | 0.000 | 0.001 |
| LHX1 | 0.136 | 0.246 | 0.860 | 0.000 | 0.000 |
| RGS20 | 0.462 | 0.838 | 0.859 | 0.000 | 0.001 |
| HOXB6 | 2.778 | 5.034 | 0.858 | 0.000 | 0.000 |
| AREG | 27.552 | 49.920 | 0.857 | 0.000 | 0.000 |
| AC243967.1 | 0.067 | 0.122 | 0.857 | 0.003 | 0.010 |
| CATSPER1 | 0.829 | 1.500 | 0.856 | 0.000 | 0.000 |
| AC009163.5 | 0.058 | 0.105 | 0.855 | 0.002 | 0.009 |
| ZNF114 | 0.874 | 1.578 | 0.853 | 0.002 | 0.007 |
| GBX1 | 0.058 | 0.104 | 0.853 | 0.003 | 0.012 |
| PCDHGA8 | 0.084 | 0.151 | 0.853 | 0.004 | 0.013 |
| SMCO2 | 0.144 | 0.261 | 0.852 | 0.000 | 0.000 |
| STEAP2 | 3.391 | 6.105 | 0.848 | 0.000 | 0.000 |
| SLCO4A1 | 2.315 | 4.167 | 0.848 | 0.000 | 0.000 |
| SLC2A1 | 27.850 | 50.129 | 0.848 | 0.000 | 0.000 |
| CA2 | 9.919 | 17.842 | 0.847 | 0.008 | 0.025 |
| VAT1L | 0.354 | 0.636 | 0.847 | 0.012 | 0.033 |
| RNASE7 | 0.140 | 0.252 | 0.846 | 0.000 | 0.000 |
| STEAP1B | 0.582 | 1.044 | 0.844 | 0.001 | 0.003 |
| FOXB1 | 0.327 | 0.586 | 0.844 | 0.012 | 0.033 |
| VSIG1 | 12.097 | 21.598 | 0.836 | 0.000 | 0.000 |
| FBXO47 | 0.023 | 0.041 | 0.836 | 0.000 | 0.002 |
| XDH | 2.832 | 5.054 | 0.836 | 0.000 | 0.000 |
| TRNP1 | 10.529 | 18.781 | 0.835 | 0.000 | 0.000 |
| NT5DC4 | 0.074 | 0.132 | 0.833 | 0.000 | 0.000 |
| CST1 | 19.599 | 34.846 | 0.830 | 0.001 | 0.004 |
| STPG4 | 0.077 | 0.137 | 0.827 | 0.000 | 0.000 |
| CDX2 | 0.220 | 0.390 | 0.826 | 0.001 | 0.004 |
| PCDH7 | 1.855 | 3.286 | 0.825 | 0.000 | 0.000 |
| GRIN2D | 1.183 | 2.090 | 0.822 | 0.000 | 0.000 |
| CARD11 | 5.534 | 9.780 | 0.821 | 0.000 | 0.000 |
| PGBD5 | 0.619 | 1.092 | 0.820 | 0.000 | 0.000 |
| NPTX2 | 2.108 | 3.716 | 0.818 | 0.001 | 0.003 |
| PLPP4 | 1.061 | 1.867 | 0.815 | 0.000 | 0.000 |
| LHFPL5 | 0.096 | 0.169 | 0.814 | 0.000 | 0.000 |
| KRT80 | 11.428 | 20.081 | 0.813 | 0.000 | 0.000 |
| IFNE | 0.148 | 0.260 | 0.812 | 0.000 | 0.002 |
| GCM1 | 0.111 | 0.196 | 0.812 | 0.000 | 0.000 |
| TNS4 | 6.034 | 10.571 | 0.809 | 0.000 | 0.000 |
| NLRP12 | 0.339 | 0.594 | 0.809 | 0.000 | 0.000 |
| CDH17 | 2.787 | 4.878 | 0.808 | 0.006 | 0.019 |
| F2RL1 | 8.144 | 14.227 | 0.805 | 0.000 | 0.000 |
| MMP7 | 35.677 | 62.317 | 0.805 | 0.001 | 0.004 |
| PLEK2 | 9.223 | 16.072 | 0.801 | 0.000 | 0.000 |
| STYK1 | 2.244 | 3.910 | 0.801 | 0.000 | 0.000 |
| GRAMD1B | 1.183 | 2.056 | 0.797 | 0.000 | 0.000 |
| HTR3A | 2.508 | 4.353 | 0.795 | 0.003 | 0.010 |
| ALDH1L1 | 0.307 | 0.532 | 0.794 | 0.000 | 0.000 |
| AC008750.7 | 0.031 | 0.053 | 0.794 | 0.000 | 0.001 |
| SH2D5 | 0.104 | 0.181 | 0.794 | 0.000 | 0.000 |
| PDLIM4 | 5.326 | 9.226 | 0.793 | 0.000 | 0.000 |
| SIM1 | 0.032 | 0.056 | 0.792 | 0.002 | 0.007 |
| BPIFC | 0.014 | 0.024 | 0.791 | 0.004 | 0.015 |
| HOXA1 | 0.632 | 1.092 | 0.790 | 0.000 | 0.000 |
| NOX1 | 0.671 | 1.160 | 0.790 | 0.004 | 0.013 |
| WNT10A | 1.436 | 2.481 | 0.789 | 0.000 | 0.000 |
| PLCD3 | 2.159 | 3.727 | 0.788 | 0.000 | 0.000 |
| TNNI3 | 1.323 | 2.283 | 0.787 | 0.000 | 0.000 |
| KIAA0319 | 0.870 | 1.500 | 0.787 | 0.004 | 0.015 |
| AL136531.2 | 0.165 | 0.285 | 0.784 | 0.000 | 0.000 |
| AFAP1L2 | 2.895 | 4.982 | 0.783 | 0.000 | 0.000 |
| KRT79 | 0.067 | 0.115 | 0.781 | 0.000 | 0.003 |
| ATP6V1FNB | 0.438 | 0.752 | 0.779 | 0.000 | 0.000 |
| BEND6 | 0.376 | 0.644 | 0.779 | 0.000 | 0.000 |
| SEMA7A | 3.801 | 6.518 | 0.778 | 0.000 | 0.000 |
| EDN2 | 3.338 | 5.723 | 0.778 | 0.000 | 0.001 |
| PPARG | 3.333 | 5.712 | 0.777 | 0.000 | 0.000 |
| CST2 | 2.765 | 4.732 | 0.775 | 0.005 | 0.017 |
| LIPK | 0.188 | 0.322 | 0.774 | 0.000 | 0.000 |
| CACNA1E | 0.040 | 0.068 | 0.773 | 0.000 | 0.002 |
| IL1RN | 4.935 | 8.428 | 0.772 | 0.000 | 0.000 |
| ITGB3 | 1.148 | 1.957 | 0.770 | 0.000 | 0.002 |
| UNC5A | 0.212 | 0.362 | 0.769 | 0.003 | 0.012 |
| DNAH3 | 0.410 | 0.699 | 0.769 | 0.000 | 0.000 |
| KLF14 | 0.045 | 0.077 | 0.768 | 0.013 | 0.036 |
| ACKR4 | 0.394 | 0.669 | 0.764 | 0.001 | 0.004 |
| SPP1 | 212.129 | 359.872 | 0.763 | 0.000 | 0.000 |
| FNDC11 | 0.268 | 0.454 | 0.762 | 0.000 | 0.000 |
| LRRN1 | 2.417 | 4.098 | 0.762 | 0.017 | 0.045 |
| C17orf64 | 0.136 | 0.231 | 0.761 | 0.000 | 0.001 |
| UTS2 | 0.203 | 0.343 | 0.759 | 0.000 | 0.000 |
| NCAN | 0.010 | 0.017 | 0.758 | 0.015 | 0.039 |
| B3GNT3 | 16.114 | 27.221 | 0.756 | 0.000 | 0.000 |
| CHST6 | 1.213 | 2.048 | 0.756 | 0.000 | 0.000 |
| SLC30A2 | 0.282 | 0.476 | 0.756 | 0.000 | 0.002 |
| TNNT1 | 8.433 | 14.224 | 0.754 | 0.000 | 0.000 |
| SLC5A11 | 0.096 | 0.161 | 0.753 | 0.011 | 0.032 |
| SLC23A1 | 0.342 | 0.577 | 0.753 | 0.005 | 0.016 |
| RSPH14 | 0.277 | 0.466 | 0.752 | 0.000 | 0.001 |
| FGF16 | 0.019 | 0.032 | 0.752 | 0.011 | 0.031 |
| RAET1L | 0.151 | 0.254 | 0.751 | 0.000 | 0.000 |
| PLA2G2F | 0.053 | 0.089 | 0.750 | 0.000 | 0.000 |
| KRT39 | 0.100 | 0.169 | 0.749 | 0.001 | 0.006 |
| LAMB3 | 70.597 | 118.619 | 0.749 | 0.000 | 0.000 |
| TINAGL1 | 7.428 | 12.464 | 0.747 | 0.000 | 0.000 |
| TNFSF9 | 2.633 | 4.417 | 0.746 | 0.000 | 0.000 |
| KLHDC7B | 1.923 | 3.223 | 0.745 | 0.001 | 0.006 |
| ASPHD2 | 1.522 | 2.546 | 0.742 | 0.000 | 0.000 |
| GATA3 | 1.495 | 2.501 | 0.742 | 0.000 | 0.000 |
| L1CAM | 0.656 | 1.096 | 0.741 | 0.000 | 0.001 |
| PRSS1 | 1.619 | 2.706 | 0.741 | 0.005 | 0.016 |
| FSIP2 | 0.103 | 0.172 | 0.739 | 0.003 | 0.011 |
| MPO | 0.127 | 0.212 | 0.738 | 0.000 | 0.001 |
| MT1M | 2.807 | 4.679 | 0.737 | 0.002 | 0.009 |
| MGAM | 0.099 | 0.164 | 0.736 | 0.000 | 0.001 |
| SLC8A2 | 0.145 | 0.241 | 0.735 | 0.001 | 0.004 |
| SAA4 | 0.432 | 0.720 | 0.735 | 0.000 | 0.000 |
| ULBP2 | 1.434 | 2.386 | 0.735 | 0.000 | 0.000 |
| ITGA6 | 10.887 | 18.097 | 0.733 | 0.000 | 0.000 |
| SNORC | 0.387 | 0.644 | 0.733 | 0.000 | 0.000 |
| KLK4 | 0.119 | 0.197 | 0.729 | 0.001 | 0.005 |
| SLC2A10 | 5.002 | 8.284 | 0.728 | 0.000 | 0.000 |
| TRIM29 | 3.131 | 5.182 | 0.727 | 0.000 | 0.000 |
| FBXO17 | 1.247 | 2.064 | 0.727 | 0.000 | 0.000 |
| GSDME | 1.370 | 2.267 | 0.726 | 0.000 | 0.000 |
| GBP7 | 0.033 | 0.055 | 0.726 | 0.001 | 0.005 |
| EPHB2 | 2.592 | 4.284 | 0.725 | 0.000 | 0.000 |
| TBX21 | 0.872 | 1.438 | 0.723 | 0.009 | 0.027 |
| COL7A1 | 2.140 | 3.528 | 0.721 | 0.000 | 0.001 |
| CCNE1 | 2.968 | 4.887 | 0.719 | 0.000 | 0.000 |
| CPNE4 | 0.494 | 0.813 | 0.719 | 0.000 | 0.000 |
| KRT78 | 0.160 | 0.263 | 0.717 | 0.000 | 0.000 |
| SYT12 | 2.368 | 3.888 | 0.715 | 0.000 | 0.000 |
| PRSS21 | 2.485 | 4.075 | 0.714 | 0.009 | 0.028 |
| OAS1 | 15.423 | 25.276 | 0.713 | 0.000 | 0.000 |
| KPNA7 | 1.867 | 3.059 | 0.712 | 0.000 | 0.000 |
| LIPH | 14.060 | 23.020 | 0.711 | 0.000 | 0.000 |
| KIAA1549L | 0.197 | 0.322 | 0.710 | 0.000 | 0.000 |
| SLC5A12 | 0.129 | 0.211 | 0.705 | 0.000 | 0.002 |
| METTL7B | 9.314 | 15.169 | 0.704 | 0.000 | 0.000 |
| GBX2 | 0.081 | 0.132 | 0.703 | 0.000 | 0.002 |
| RFX8 | 0.249 | 0.404 | 0.702 | 0.000 | 0.002 |
| ARHGEF4 | 0.826 | 1.340 | 0.698 | 0.000 | 0.000 |
| CD70 | 0.654 | 1.061 | 0.698 | 0.000 | 0.000 |
| PRSS22 | 10.213 | 16.567 | 0.698 | 0.000 | 0.000 |
| FAM81A | 0.648 | 1.051 | 0.697 | 0.000 | 0.000 |
| ERO1A | 24.882 | 40.290 | 0.695 | 0.000 | 0.000 |
| HOXB7 | 6.058 | 9.801 | 0.694 | 0.000 | 0.000 |
| C11orf86 | 1.937 | 3.133 | 0.694 | 0.000 | 0.000 |
| MT2A | 82.190 | 132.784 | 0.692 | 0.000 | 0.000 |
| ZFP57 | 0.512 | 0.825 | 0.689 | 0.000 | 0.000 |
| BCAN | 0.361 | 0.581 | 0.689 | 0.000 | 0.002 |
| ACVR1C | 0.160 | 0.257 | 0.686 | 0.000 | 0.000 |
| RPL39L | 12.022 | 19.336 | 0.686 | 0.000 | 0.000 |
| PLOD2 | 12.967 | 20.853 | 0.685 | 0.000 | 0.000 |
| C1QL1 | 0.420 | 0.675 | 0.685 | 0.000 | 0.000 |
| IGSF11 | 0.135 | 0.217 | 0.684 | 0.002 | 0.007 |
| FABP6 | 3.098 | 4.974 | 0.683 | 0.000 | 0.000 |
| AC008687.4 | 0.267 | 0.428 | 0.680 | 0.000 | 0.000 |
| NOX5 | 0.125 | 0.201 | 0.679 | 0.000 | 0.000 |
| PPP1R36 | 0.364 | 0.583 | 0.679 | 0.000 | 0.000 |
| ITGB4 | 29.011 | 46.430 | 0.678 | 0.000 | 0.000 |
| ITGA5 | 11.718 | 18.753 | 0.678 | 0.000 | 0.000 |
| GPER1 | 0.873 | 1.397 | 0.678 | 0.001 | 0.004 |
| ZNF365 | 0.195 | 0.312 | 0.677 | 0.000 | 0.001 |
| GALNT3 | 12.267 | 19.611 | 0.677 | 0.000 | 0.000 |
| ALX1 | 0.213 | 0.340 | 0.675 | 0.000 | 0.002 |
| ADTRP | 0.908 | 1.449 | 0.675 | 0.000 | 0.000 |
| SEC16B | 0.031 | 0.050 | 0.675 | 0.000 | 0.000 |
| IFNL1 | 0.071 | 0.113 | 0.673 | 0.000 | 0.002 |
| MFSD6L | 2.041 | 3.254 | 0.673 | 0.001 | 0.005 |
| IGF2BP2 | 4.870 | 7.762 | 0.672 | 0.000 | 0.000 |
| PRPH | 0.062 | 0.099 | 0.672 | 0.007 | 0.022 |
| SPRR2F | 0.802 | 1.277 | 0.670 | 0.001 | 0.003 |
| KLHDC8A | 0.559 | 0.889 | 0.670 | 0.010 | 0.028 |
| SAA2 | 2.793 | 4.443 | 0.669 | 0.000 | 0.000 |
| RNF128 | 6.587 | 10.475 | 0.669 | 0.002 | 0.008 |
| MUC20 | 10.745 | 17.077 | 0.668 | 0.000 | 0.000 |
| SH3RF2 | 1.591 | 2.528 | 0.668 | 0.000 | 0.000 |
| SYT13 | 2.792 | 4.434 | 0.667 | 0.000 | 0.000 |
| ENTHD1 | 0.099 | 0.158 | 0.667 | 0.001 | 0.004 |
| GJB6 | 1.656 | 2.629 | 0.667 | 0.000 | 0.000 |
| TNNI2 | 1.757 | 2.789 | 0.667 | 0.001 | 0.006 |
| ATG9B | 0.933 | 1.480 | 0.666 | 0.000 | 0.000 |
| CST6 | 14.578 | 23.098 | 0.664 | 0.000 | 0.000 |
| PRDM8 | 0.803 | 1.272 | 0.664 | 0.000 | 0.000 |
| FHOD3 | 0.886 | 1.402 | 0.663 | 0.000 | 0.000 |
| SEMA3C | 8.565 | 13.556 | 0.662 | 0.000 | 0.000 |
| MAP7D2 | 2.042 | 3.231 | 0.662 | 0.000 | 0.001 |
| TUBAL3 | 0.351 | 0.555 | 0.661 | 0.000 | 0.000 |
| RHPN2 | 6.568 | 10.372 | 0.659 | 0.000 | 0.000 |
| FOXL2NB | 0.026 | 0.041 | 0.659 | 0.000 | 0.000 |
| CRLF2 | 0.403 | 0.637 | 0.658 | 0.000 | 0.001 |
| GPAT3 | 2.500 | 3.943 | 0.658 | 0.000 | 0.001 |
| BIRC3 | 11.319 | 17.853 | 0.657 | 0.000 | 0.000 |
| PCDHAC2 | 0.432 | 0.681 | 0.657 | 0.000 | 0.001 |
| FOXE3 | 0.063 | 0.099 | 0.656 | 0.000 | 0.000 |
| PRR18 | 0.184 | 0.290 | 0.655 | 0.001 | 0.004 |
| IFI27 | 56.917 | 89.624 | 0.655 | 0.000 | 0.000 |
| PKP2 | 2.336 | 3.676 | 0.654 | 0.000 | 0.000 |
| GALNT5 | 4.348 | 6.843 | 0.654 | 0.000 | 0.000 |
| ITPKA | 5.657 | 8.899 | 0.654 | 0.000 | 0.000 |
| C6orf141 | 1.453 | 2.284 | 0.653 | 0.000 | 0.000 |
| FTCD | 0.208 | 0.327 | 0.653 | 0.004 | 0.013 |
| ASAP2 | 1.971 | 3.099 | 0.652 | 0.000 | 0.000 |
| RHO | 0.008 | 0.012 | 0.652 | 0.002 | 0.007 |
| HES2 | 0.689 | 1.082 | 0.651 | 0.002 | 0.008 |
| BEAN1 | 1.176 | 1.845 | 0.650 | 0.000 | 0.000 |
| RDH5 | 0.254 | 0.398 | 0.649 | 0.000 | 0.000 |
| DEUP1 | 0.204 | 0.320 | 0.648 | 0.000 | 0.001 |
| ELOVL6 | 2.151 | 3.367 | 0.647 | 0.000 | 0.000 |
| PRAC2 | 0.284 | 0.444 | 0.646 | 0.001 | 0.004 |
| UNC13D | 7.345 | 11.493 | 0.646 | 0.000 | 0.000 |
| UPP1 | 12.497 | 19.554 | 0.646 | 0.000 | 0.000 |
| FSCN1 | 26.486 | 41.402 | 0.644 | 0.000 | 0.000 |
| STAC | 1.381 | 2.159 | 0.644 | 0.000 | 0.000 |
| ACOT11 | 0.750 | 1.172 | 0.644 | 0.000 | 0.000 |
| PLA1A | 3.457 | 5.393 | 0.642 | 0.001 | 0.004 |
| GPR35 | 1.605 | 2.502 | 0.640 | 0.000 | 0.001 |
| HS3ST1 | 2.602 | 4.049 | 0.638 | 0.000 | 0.000 |
| LHB | 0.368 | 0.573 | 0.638 | 0.000 | 0.001 |
| DCDC1 | 0.058 | 0.090 | 0.635 | 0.010 | 0.030 |
| GALNT4 | 0.235 | 0.365 | 0.635 | 0.000 | 0.001 |
| AK4 | 2.991 | 4.643 | 0.634 | 0.000 | 0.000 |
| GREB1L | 0.389 | 0.604 | 0.634 | 0.000 | 0.000 |
| CACNG4 | 4.501 | 6.984 | 0.634 | 0.000 | 0.001 |
| NAV3 | 0.340 | 0.526 | 0.631 | 0.011 | 0.030 |
| FCHO1 | 2.028 | 3.141 | 0.631 | 0.000 | 0.000 |
| GALNT6 | 8.653 | 13.399 | 0.631 | 0.000 | 0.000 |
| PPP2R2C | 1.107 | 1.713 | 0.631 | 0.000 | 0.002 |
| CXCL11 | 3.918 | 6.063 | 0.630 | 0.000 | 0.001 |
| MT1H | 1.092 | 1.690 | 0.630 | 0.006 | 0.021 |
| MYH15 | 0.144 | 0.223 | 0.629 | 0.001 | 0.004 |
| DMP1 | 0.036 | 0.056 | 0.629 | 0.002 | 0.006 |
| SOWAHA | 0.197 | 0.305 | 0.629 | 0.001 | 0.005 |
| LDHAL6B | 0.065 | 0.100 | 0.628 | 0.004 | 0.014 |
| FERMT1 | 4.878 | 7.535 | 0.627 | 0.000 | 0.003 |
| SLC28A3 | 1.009 | 1.557 | 0.626 | 0.000 | 0.000 |
| FAM71F1 | 0.023 | 0.035 | 0.624 | 0.000 | 0.000 |
| CCNB3 | 0.107 | 0.164 | 0.624 | 0.000 | 0.002 |
| ABCA12 | 1.186 | 1.827 | 0.624 | 0.000 | 0.000 |
| FBXO39 | 0.081 | 0.124 | 0.623 | 0.000 | 0.001 |
| MTCL1 | 0.703 | 1.082 | 0.622 | 0.000 | 0.000 |
| MYO3A | 0.095 | 0.146 | 0.622 | 0.017 | 0.044 |
| HS3ST3A1 | 0.451 | 0.693 | 0.621 | 0.000 | 0.000 |
| RHBDL2 | 3.199 | 4.919 | 0.621 | 0.000 | 0.000 |
| AC010980.1 | 0.200 | 0.308 | 0.621 | 0.000 | 0.000 |
| MYEOV | 4.548 | 6.986 | 0.619 | 0.000 | 0.001 |
| HMGA1 | 97.916 | 150.333 | 0.619 | 0.000 | 0.000 |
| KCNN4 | 14.433 | 22.153 | 0.618 | 0.000 | 0.000 |
| VEGFC | 3.435 | 5.267 | 0.617 | 0.010 | 0.028 |
| HIGD2B | 0.020 | 0.031 | 0.616 | 0.000 | 0.002 |
| RASSF6 | 1.932 | 2.960 | 0.616 | 0.000 | 0.000 |
| GPR1 | 0.250 | 0.382 | 0.615 | 0.000 | 0.000 |
| CALB2 | 2.010 | 3.079 | 0.615 | 0.000 | 0.000 |
| MMP14 | 62.851 | 96.257 | 0.615 | 0.000 | 0.000 |
| KRT23 | 2.223 | 3.402 | 0.614 | 0.000 | 0.000 |
| LIPM | 0.764 | 1.169 | 0.613 | 0.000 | 0.000 |
| MET | 35.198 | 53.833 | 0.613 | 0.000 | 0.000 |
| MTMR11 | 5.136 | 7.854 | 0.613 | 0.000 | 0.000 |
| BCL2L15 | 2.816 | 4.303 | 0.612 | 0.000 | 0.000 |
| SPRR2D | 2.937 | 4.487 | 0.612 | 0.000 | 0.000 |
| GBP3 | 9.843 | 15.038 | 0.611 | 0.000 | 0.000 |
| NPSR1 | 0.150 | 0.229 | 0.610 | 0.000 | 0.000 |
| SOX9 | 8.073 | 12.317 | 0.609 | 0.000 | 0.000 |
| CCDC103 | 0.005 | 0.007 | 0.609 | 0.000 | 0.000 |
| TPRG1 | 0.412 | 0.628 | 0.609 | 0.000 | 0.000 |
| ENPP1 | 0.717 | 1.093 | 0.608 | 0.000 | 0.000 |
| IDO1 | 12.305 | 18.741 | 0.607 | 0.001 | 0.005 |
| ABCA13 | 0.627 | 0.954 | 0.607 | 0.001 | 0.003 |
| POTEF | 0.032 | 0.049 | 0.606 | 0.000 | 0.001 |
| A1CF | 0.074 | 0.112 | 0.604 | 0.001 | 0.003 |
| TDRD5 | 0.760 | 1.155 | 0.603 | 0.013 | 0.035 |
| DRC1 | 1.024 | 1.556 | 0.603 | 0.012 | 0.034 |
| EFNB1 | 8.115 | 12.321 | 0.602 | 0.000 | 0.000 |
| NUP62CL | 1.571 | 2.385 | 0.602 | 0.000 | 0.000 |
| LOXL2 | 8.753 | 13.284 | 0.602 | 0.001 | 0.005 |
| PCDHGC5 | 0.085 | 0.129 | 0.602 | 0.001 | 0.003 |
| AIFM3 | 0.522 | 0.792 | 0.602 | 0.001 | 0.004 |
| FHAD1 | 0.411 | 0.624 | 0.602 | 0.000 | 0.000 |
| PKIB | 3.777 | 5.729 | 0.601 | 0.000 | 0.000 |
| IL23A | 2.016 | 3.056 | 0.600 | 0.000 | 0.000 |
| CAVIN3 | 8.522 | 12.910 | 0.599 | 0.000 | 0.000 |
| TWIST1 | 1.216 | 1.842 | 0.599 | 0.000 | 0.000 |
| SYT1 | 1.342 | 2.032 | 0.599 | 0.001 | 0.005 |
| KCNG1 | 0.146 | 0.221 | 0.598 | 0.000 | 0.000 |
| GSDMB | 4.153 | 6.283 | 0.597 | 0.000 | 0.000 |
| C16orf90 | 0.022 | 0.034 | 0.596 | 0.003 | 0.011 |
| TEX35 | 0.018 | 0.027 | 0.595 | 0.004 | 0.013 |
| TUBB6 | 10.131 | 15.302 | 0.595 | 0.000 | 0.000 |
| PLEKHG6 | 2.835 | 4.276 | 0.593 | 0.000 | 0.000 |
| UGT8 | 2.743 | 4.136 | 0.593 | 0.000 | 0.000 |
| PCED1B | 6.000 | 9.044 | 0.592 | 0.000 | 0.000 |
| ANLN | 6.806 | 10.256 | 0.591 | 0.000 | 0.000 |
| QPRT | 12.065 | 18.175 | 0.591 | 0.000 | 0.000 |
| CCL8 | 3.175 | 4.779 | 0.590 | 0.000 | 0.000 |
| CLIP2 | 2.597 | 3.905 | 0.588 | 0.000 | 0.000 |
| ISL2 | 0.163 | 0.245 | 0.588 | 0.002 | 0.007 |
| CCL17 | 4.816 | 7.239 | 0.588 | 0.010 | 0.030 |
| ACY3 | 2.696 | 4.052 | 0.588 | 0.000 | 0.000 |
| PIP | 3.308 | 4.971 | 0.588 | 0.000 | 0.000 |
| FAP | 2.781 | 4.174 | 0.586 | 0.000 | 0.000 |
| LHX5 | 0.141 | 0.211 | 0.585 | 0.000 | 0.000 |
| SLAMF9 | 0.838 | 1.257 | 0.584 | 0.000 | 0.000 |
| FHL2 | 8.350 | 12.511 | 0.583 | 0.000 | 0.000 |
| INSL3 | 0.406 | 0.608 | 0.583 | 0.000 | 0.000 |
| S100A10 | 252.630 | 378.127 | 0.582 | 0.000 | 0.000 |
| SPHK1 | 3.119 | 4.668 | 0.582 | 0.000 | 0.000 |
| KIF5A | 0.271 | 0.405 | 0.581 | 0.000 | 0.000 |
| AHNAK2 | 4.995 | 7.472 | 0.581 | 0.000 | 0.000 |
| INPP4B | 1.851 | 2.768 | 0.580 | 0.000 | 0.000 |
| CGB7 | 0.113 | 0.169 | 0.580 | 0.000 | 0.000 |
| DGAT2L6 | 0.031 | 0.046 | 0.580 | 0.004 | 0.015 |
| HOXA2 | 0.384 | 0.574 | 0.579 | 0.000 | 0.000 |
| RHBG | 0.289 | 0.432 | 0.578 | 0.000 | 0.002 |
| COL12A1 | 10.210 | 15.243 | 0.578 | 0.020 | 0.050 |
| TGFA | 7.101 | 10.597 | 0.578 | 0.000 | 0.000 |
| SMC1B | 0.482 | 0.719 | 0.577 | 0.000 | 0.001 |
| TMPRSS4 | 10.561 | 15.752 | 0.577 | 0.000 | 0.000 |
| MYO1E | 6.654 | 9.922 | 0.576 | 0.000 | 0.000 |
| COL10A1 | 14.240 | 21.227 | 0.576 | 0.007 | 0.023 |
| SLC28A1 | 0.060 | 0.089 | 0.575 | 0.008 | 0.025 |
| AMIGO2 | 15.766 | 23.472 | 0.574 | 0.000 | 0.000 |
| ROPN1B | 0.132 | 0.197 | 0.574 | 0.001 | 0.004 |
| LYZ | 262.431 | 390.228 | 0.572 | 0.003 | 0.012 |
| TMIE | 0.391 | 0.581 | 0.572 | 0.000 | 0.000 |
| FRMD5 | 1.035 | 1.539 | 0.572 | 0.000 | 0.000 |
| IGFL2 | 0.945 | 1.403 | 0.571 | 0.003 | 0.013 |
| STRC | 0.022 | 0.033 | 0.570 | 0.008 | 0.024 |
| CALML4 | 0.830 | 1.231 | 0.568 | 0.000 | 0.002 |
| HOGA1 | 0.156 | 0.231 | 0.568 | 0.002 | 0.008 |
| KIRREL3 | 0.105 | 0.155 | 0.568 | 0.000 | 0.001 |
| EPHA2 | 15.705 | 23.273 | 0.567 | 0.000 | 0.000 |
| SERPINE1 | 30.580 | 45.316 | 0.567 | 0.000 | 0.000 |
| PCDHAC1 | 0.478 | 0.708 | 0.566 | 0.000 | 0.001 |
| NR2E1 | 0.081 | 0.119 | 0.565 | 0.000 | 0.001 |
| HHIPL1 | 0.486 | 0.718 | 0.563 | 0.000 | 0.000 |
| COL6A1 | 50.946 | 75.274 | 0.563 | 0.000 | 0.000 |
| KRT19 | 281.209 | 415.332 | 0.563 | 0.000 | 0.000 |
| BATF2 | 4.856 | 7.168 | 0.562 | 0.000 | 0.000 |
| E2F7 | 0.634 | 0.936 | 0.562 | 0.000 | 0.000 |
| GALNT14 | 4.022 | 5.935 | 0.561 | 0.000 | 0.000 |
| NLRP2 | 3.836 | 5.659 | 0.561 | 0.005 | 0.018 |
| EFHD2 | 39.378 | 58.091 | 0.561 | 0.000 | 0.000 |
| RTP4 | 4.226 | 6.234 | 0.561 | 0.000 | 0.000 |
| MMP8 | 0.143 | 0.211 | 0.561 | 0.018 | 0.046 |
| SLC2A3 | 9.233 | 13.617 | 0.561 | 0.000 | 0.000 |
| SPRR1B | 12.070 | 17.792 | 0.560 | 0.000 | 0.000 |
| SAMD9 | 4.056 | 5.979 | 0.560 | 0.000 | 0.000 |
| HNF4G | 1.594 | 2.349 | 0.560 | 0.000 | 0.000 |
| INAVA | 6.957 | 10.253 | 0.559 | 0.000 | 0.000 |
| IVL | 3.627 | 5.340 | 0.558 | 0.000 | 0.001 |
| TMEM132B | 0.041 | 0.060 | 0.558 | 0.002 | 0.009 |
| KBTBD12 | 0.612 | 0.900 | 0.558 | 0.001 | 0.004 |
| B3GALNT1 | 2.967 | 4.365 | 0.557 | 0.000 | 0.000 |
| ADAMTS3 | 0.436 | 0.641 | 0.556 | 0.006 | 0.019 |
| SPATS2L | 11.368 | 16.715 | 0.556 | 0.000 | 0.000 |
| FUT4 | 1.760 | 2.587 | 0.556 | 0.000 | 0.000 |
| TFR2 | 0.647 | 0.951 | 0.555 | 0.001 | 0.005 |
| CKLF-CMTM1 | 0.262 | 0.384 | 0.555 | 0.000 | 0.000 |
| RTL3 | 0.025 | 0.037 | 0.554 | 0.011 | 0.032 |
| MSX2 | 0.876 | 1.285 | 0.554 | 0.000 | 0.000 |
| MFSD4A | 7.921 | 11.622 | 0.553 | 0.002 | 0.007 |
| DNAAF3 | 0.905 | 1.328 | 0.553 | 0.000 | 0.000 |
| FXYD5 | 30.709 | 45.024 | 0.552 | 0.000 | 0.000 |
| TUBA4A | 20.290 | 29.737 | 0.551 | 0.000 | 0.000 |
| TSKU | 16.065 | 23.541 | 0.551 | 0.000 | 0.000 |
| PRLR | 0.734 | 1.074 | 0.550 | 0.001 | 0.004 |
| CCIN | 0.096 | 0.140 | 0.550 | 0.000 | 0.002 |
| C11orf91 | 0.117 | 0.171 | 0.550 | 0.000 | 0.000 |
| ZNF185 | 8.341 | 12.208 | 0.550 | 0.000 | 0.000 |
| EXOC3L4 | 0.392 | 0.574 | 0.549 | 0.008 | 0.024 |
| KAAG1 | 0.053 | 0.078 | 0.547 | 0.004 | 0.015 |
| PLAUR | 17.413 | 25.418 | 0.546 | 0.000 | 0.000 |
| PSMB9 | 19.147 | 27.936 | 0.545 | 0.000 | 0.000 |
| LPAR5 | 1.692 | 2.466 | 0.544 | 0.000 | 0.000 |
| SLC5A5 | 1.267 | 1.846 | 0.543 | 0.001 | 0.006 |
| NIPAL1 | 0.816 | 1.189 | 0.543 | 0.000 | 0.002 |
| AP1S3 | 2.531 | 3.685 | 0.542 | 0.000 | 0.000 |
| NEDD4 | 1.528 | 2.225 | 0.542 | 0.000 | 0.000 |
| TNFRSF12A | 41.090 | 59.810 | 0.542 | 0.000 | 0.000 |
| PMEPA1 | 19.971 | 29.068 | 0.542 | 0.000 | 0.000 |
| CMTM1 | 0.743 | 1.081 | 0.541 | 0.000 | 0.000 |
| SLC9A7 | 2.664 | 3.873 | 0.540 | 0.000 | 0.000 |
| CCDC74B | 0.298 | 0.434 | 0.540 | 0.000 | 0.000 |
| S100A16 | 106.916 | 155.389 | 0.539 | 0.000 | 0.000 |
| GDPD4 | 0.051 | 0.074 | 0.539 | 0.001 | 0.004 |
| CYS1 | 2.107 | 3.060 | 0.538 | 0.005 | 0.017 |
| ILDR2 | 0.158 | 0.230 | 0.538 | 0.004 | 0.016 |
| DRAXIN | 0.108 | 0.157 | 0.538 | 0.000 | 0.000 |
| SLC16A3 | 18.657 | 27.089 | 0.538 | 0.000 | 0.000 |
| CHRNB4 | 0.129 | 0.187 | 0.538 | 0.000 | 0.000 |
| SLC45A3 | 3.390 | 4.921 | 0.538 | 0.000 | 0.000 |
| TMEM40 | 0.517 | 0.751 | 0.537 | 0.000 | 0.000 |
| RAG1 | 0.215 | 0.313 | 0.537 | 0.000 | 0.000 |
| LAMA1 | 0.413 | 0.600 | 0.537 | 0.004 | 0.013 |
| PRRX2 | 3.793 | 5.504 | 0.537 | 0.000 | 0.000 |
| LPCAT4 | 6.633 | 9.623 | 0.537 | 0.009 | 0.027 |
| LRRC43 | 0.713 | 1.034 | 0.535 | 0.000 | 0.001 |
| MMP3 | 0.956 | 1.384 | 0.535 | 0.000 | 0.000 |
| SYTL4 | 1.723 | 2.495 | 0.534 | 0.000 | 0.000 |
| ENKUR | 0.807 | 1.168 | 0.533 | 0.000 | 0.002 |
| UBE2C | 26.866 | 38.869 | 0.533 | 0.000 | 0.001 |
| NT5E | 14.956 | 21.636 | 0.533 | 0.000 | 0.000 |
| MT1E | 30.318 | 43.842 | 0.532 | 0.002 | 0.009 |
| ANXA4 | 13.311 | 19.239 | 0.531 | 0.000 | 0.000 |
| NPAS2 | 1.849 | 2.670 | 0.530 | 0.000 | 0.000 |
| RGS8 | 0.014 | 0.020 | 0.529 | 0.001 | 0.006 |
| ANKRD22 | 9.426 | 13.601 | 0.529 | 0.000 | 0.000 |
| IFI35 | 20.948 | 30.224 | 0.529 | 0.000 | 0.000 |
| BHLHE41 | 4.845 | 6.986 | 0.528 | 0.000 | 0.000 |
| PTGER2 | 2.349 | 3.387 | 0.528 | 0.000 | 0.002 |
| STX1B | 0.210 | 0.302 | 0.528 | 0.000 | 0.000 |
| B4GALT4 | 4.153 | 5.987 | 0.527 | 0.000 | 0.000 |
| SPRY3 | 0.370 | 0.533 | 0.526 | 0.005 | 0.018 |
| OAS3 | 13.779 | 19.836 | 0.526 | 0.000 | 0.000 |
| DDX60L | 2.315 | 3.330 | 0.524 | 0.000 | 0.000 |
| RIMS2 | 0.187 | 0.268 | 0.523 | 0.000 | 0.000 |
| PLSCR1 | 15.288 | 21.961 | 0.523 | 0.000 | 0.000 |
| ACAN | 0.310 | 0.446 | 0.522 | 0.003 | 0.012 |
| TIMP2 | 48.778 | 70.048 | 0.522 | 0.000 | 0.000 |
| PYCARD | 11.299 | 16.219 | 0.521 | 0.000 | 0.000 |
| CHRFAM7A | 0.029 | 0.041 | 0.521 | 0.000 | 0.000 |
| CXCR3 | 3.171 | 4.550 | 0.521 | 0.000 | 0.001 |
| EPHX4 | 1.485 | 2.130 | 0.521 | 0.000 | 0.000 |
| BCAR3 | 4.465 | 6.406 | 0.521 | 0.000 | 0.000 |
| ADAM8 | 12.610 | 18.085 | 0.520 | 0.000 | 0.000 |
| CNTNAP4 | 0.049 | 0.070 | 0.520 | 0.016 | 0.043 |
| STX1A | 4.285 | 6.142 | 0.519 | 0.000 | 0.000 |
| ANXA8 | 0.519 | 0.744 | 0.518 | 0.000 | 0.000 |
| TAP1 | 35.005 | 50.133 | 0.518 | 0.000 | 0.000 |
| S100A5 | 1.056 | 1.512 | 0.518 | 0.000 | 0.000 |
| SYTL2 | 3.574 | 5.116 | 0.517 | 0.000 | 0.000 |
| DAPP1 | 3.569 | 5.107 | 0.517 | 0.000 | 0.000 |
| HSPA2 | 2.205 | 3.155 | 0.517 | 0.000 | 0.002 |
| RAET1E | 0.430 | 0.615 | 0.517 | 0.000 | 0.000 |
| NEU4 | 0.081 | 0.115 | 0.517 | 0.001 | 0.005 |
| LMO1 | 0.097 | 0.139 | 0.516 | 0.001 | 0.006 |
| AMZ1 | 0.190 | 0.271 | 0.516 | 0.002 | 0.008 |
| LINC00634 | 0.138 | 0.197 | 0.515 | 0.005 | 0.016 |
| EGR4 | 0.195 | 0.279 | 0.515 | 0.004 | 0.013 |
| C4orf19 | 2.378 | 3.398 | 0.515 | 0.001 | 0.006 |
| TMEM158 | 2.036 | 2.908 | 0.515 | 0.000 | 0.000 |
| TMC7 | 1.015 | 1.450 | 0.514 | 0.000 | 0.001 |
| CD163L1 | 1.078 | 1.539 | 0.514 | 0.000 | 0.000 |
| AANAT | 0.123 | 0.176 | 0.513 | 0.001 | 0.005 |
| CCDC74A | 1.268 | 1.808 | 0.511 | 0.000 | 0.000 |
| RIN1 | 1.694 | 2.415 | 0.511 | 0.000 | 0.000 |
| KRT81 | 11.810 | 16.834 | 0.511 | 0.003 | 0.011 |
| TMEM200A | 1.423 | 2.028 | 0.511 | 0.002 | 0.009 |
| ZBED2 | 1.708 | 2.434 | 0.511 | 0.000 | 0.001 |
| CKAP2L | 1.837 | 2.617 | 0.511 | 0.000 | 0.000 |
| EPSTI1 | 5.090 | 7.250 | 0.510 | 0.000 | 0.000 |
| PLEKHA6 | 6.580 | 9.370 | 0.510 | 0.000 | 0.000 |
| TK1 | 28.957 | 41.228 | 0.510 | 0.000 | 0.000 |
| DDO | 1.166 | 1.660 | 0.509 | 0.000 | 0.000 |
| ZNF486 | 4.807 | 6.838 | 0.508 | 0.001 | 0.003 |
| TLDC2 | 1.047 | 1.489 | 0.508 | 0.000 | 0.000 |
| UBASH3B | 1.342 | 1.908 | 0.508 | 0.000 | 0.000 |
| KLHL30 | 0.429 | 0.610 | 0.508 | 0.002 | 0.008 |
| GLIS1 | 0.112 | 0.159 | 0.508 | 0.017 | 0.044 |
| COL6A2 | 72.661 | 103.241 | 0.507 | 0.000 | 0.000 |
| CLCF1 | 8.683 | 12.334 | 0.506 | 0.000 | 0.000 |
| PDZK1IP1 | 49.031 | 69.642 | 0.506 | 0.000 | 0.000 |
| EPS8L1 | 6.842 | 9.716 | 0.506 | 0.000 | 0.000 |
| ETV3L | 0.011 | 0.016 | 0.506 | 0.008 | 0.024 |
| C19orf33 | 76.322 | 108.340 | 0.505 | 0.000 | 0.000 |
| CSF2RA | 4.093 | 5.810 | 0.505 | 0.000 | 0.000 |
| HLA-F | 23.533 | 33.385 | 0.505 | 0.000 | 0.000 |
| S100A3 | 1.571 | 2.229 | 0.504 | 0.000 | 0.003 |
| ZFR2 | 0.257 | 0.364 | 0.504 | 0.006 | 0.020 |
| OAS2 | 11.499 | 16.299 | 0.503 | 0.000 | 0.000 |
| MORN3 | 0.660 | 0.936 | 0.503 | 0.001 | 0.006 |
| DOCK3 | 0.468 | 0.663 | 0.503 | 0.000 | 0.002 |
| APOL1 | 48.839 | 69.209 | 0.503 | 0.000 | 0.000 |
| CACNA1I | 0.177 | 0.250 | 0.503 | 0.000 | 0.001 |
| SMAGP | 5.065 | 7.170 | 0.501 | 0.000 | 0.000 |
| SEC14L2 | 1.576 | 2.230 | 0.501 | 0.000 | 0.000 |
| GPR19 | 0.402 | 0.568 | 0.501 | 0.000 | 0.000 |
| MT1F | 4.233 | 5.990 | 0.501 | 0.000 | 0.000 |
| FOXQ1 | 11.458 | 16.208 | 0.500 | 0.001 | 0.005 |
| TMEM156 | 2.405 | 3.402 | 0.500 | 0.001 | 0.003 |
| SLC35G5 | 0.080 | 0.057 | -0.500 | 0.000 | 0.002 |
| SOX14 | 1.043 | 0.737 | -0.501 | 0.000 | 0.000 |
| PLXNA2 | 6.575 | 4.644 | -0.501 | 0.000 | 0.000 |
| PPARGC1A | 1.146 | 0.810 | -0.502 | 0.000 | 0.000 |
| BCAM | 81.648 | 57.609 | -0.503 | 0.000 | 0.000 |
| CMA1 | 0.281 | 0.198 | -0.505 | 0.013 | 0.036 |
| SFTA2 | 264.897 | 186.566 | -0.506 | 0.001 | 0.004 |
| FZD3 | 1.287 | 0.905 | -0.507 | 0.000 | 0.000 |
| PMM1 | 10.990 | 7.728 | -0.508 | 0.000 | 0.000 |
| MTURN | 7.334 | 5.156 | -0.508 | 0.000 | 0.000 |
| PNMA2 | 6.761 | 4.753 | -0.508 | 0.000 | 0.000 |
| SLC35G1 | 1.307 | 0.918 | -0.509 | 0.001 | 0.005 |
| PHACTR1 | 1.665 | 1.169 | -0.511 | 0.001 | 0.005 |
| BPIFA2 | 5.763 | 4.037 | -0.514 | 0.001 | 0.005 |
| BHLHA15 | 5.110 | 3.576 | -0.515 | 0.000 | 0.001 |
| GSTA4 | 15.082 | 10.554 | -0.515 | 0.000 | 0.000 |
| PPP1R13B | 7.827 | 5.473 | -0.516 | 0.000 | 0.000 |
| CAVIN2 | 15.650 | 10.941 | -0.516 | 0.000 | 0.001 |
| AC012651.1 | 0.722 | 0.504 | -0.517 | 0.004 | 0.014 |
| PHACTR3 | 3.247 | 2.263 | -0.520 | 0.001 | 0.003 |
| SLC16A11 | 1.713 | 1.194 | -0.521 | 0.018 | 0.046 |
| MTSS1 | 7.994 | 5.569 | -0.522 | 0.000 | 0.000 |
| CAPN9 | 5.077 | 3.535 | -0.522 | 0.000 | 0.000 |
| LMO3 | 14.281 | 9.941 | -0.523 | 0.000 | 0.000 |
| LCN6 | 0.086 | 0.060 | -0.524 | 0.001 | 0.004 |
| GRAMD2A | 5.396 | 3.752 | -0.524 | 0.000 | 0.000 |
| PDZD2 | 2.029 | 1.408 | -0.528 | 0.000 | 0.000 |
| AQP7 | 1.298 | 0.900 | -0.529 | 0.002 | 0.009 |
| PANK1 | 1.367 | 0.947 | -0.530 | 0.000 | 0.000 |
| SNX30 | 8.060 | 5.583 | -0.530 | 0.000 | 0.000 |
| CMTM5 | 0.032 | 0.022 | -0.531 | 0.002 | 0.007 |
| SNX22 | 1.340 | 0.925 | -0.535 | 0.000 | 0.000 |
| FGG | 168.197 | 116.070 | -0.535 | 0.000 | 0.002 |
| GNMT | 0.927 | 0.639 | -0.538 | 0.000 | 0.000 |
| NEDD9 | 17.791 | 12.253 | -0.538 | 0.000 | 0.001 |
| ADHFE1 | 1.581 | 1.087 | -0.540 | 0.005 | 0.017 |
| FHL5 | 1.306 | 0.897 | -0.543 | 0.000 | 0.001 |
| PCDH20 | 0.113 | 0.078 | -0.543 | 0.000 | 0.001 |
| PTP4A3 | 36.723 | 25.167 | -0.545 | 0.000 | 0.000 |
| RASL11B | 2.483 | 1.701 | -0.546 | 0.004 | 0.013 |
| CCDC85A | 0.374 | 0.256 | -0.546 | 0.005 | 0.017 |
| TC2N | 23.853 | 16.322 | -0.547 | 0.000 | 0.000 |
| OIT3 | 0.640 | 0.438 | -0.548 | 0.001 | 0.004 |
| SLC5A2 | 0.443 | 0.303 | -0.548 | 0.000 | 0.000 |
| FAM167A | 3.285 | 2.239 | -0.553 | 0.000 | 0.002 |
| RPL17-C18orf32 | 0.016 | 0.011 | -0.554 | 0.000 | 0.002 |
| DACH1 | 0.909 | 0.618 | -0.556 | 0.001 | 0.003 |
| CACNB4 | 0.308 | 0.209 | -0.557 | 0.018 | 0.046 |
| ABLIM2 | 1.179 | 0.800 | -0.559 | 0.000 | 0.000 |
| PNMT | 0.699 | 0.474 | -0.560 | 0.013 | 0.036 |
| PTCHD4 | 0.499 | 0.338 | -0.561 | 0.000 | 0.002 |
| ALKAL2 | 0.469 | 0.318 | -0.562 | 0.007 | 0.023 |
| DACT2 | 3.169 | 2.146 | -0.562 | 0.000 | 0.001 |
| EDNRB | 4.015 | 2.718 | -0.563 | 0.000 | 0.001 |
| WNT9B | 0.051 | 0.034 | -0.566 | 0.009 | 0.027 |
| IGSF9B | 0.876 | 0.589 | -0.571 | 0.001 | 0.005 |
| CLU | 80.193 | 53.918 | -0.573 | 0.013 | 0.035 |
| NPNT | 16.211 | 10.878 | -0.576 | 0.000 | 0.001 |
| RXFP1 | 0.271 | 0.182 | -0.576 | 0.004 | 0.014 |
| CLIC3 | 20.054 | 13.448 | -0.577 | 0.018 | 0.047 |
| ST6GAL2 | 0.398 | 0.266 | -0.579 | 0.017 | 0.045 |
| MTNR1A | 0.208 | 0.139 | -0.580 | 0.007 | 0.022 |
| AL355987.3 | 0.118 | 0.079 | -0.582 | 0.001 | 0.004 |
| WFIKKN1 | 0.357 | 0.237 | -0.588 | 0.000 | 0.002 |
| SCNN1A | 78.326 | 52.117 | -0.588 | 0.000 | 0.000 |
| FGFR3 | 10.543 | 7.002 | -0.590 | 0.002 | 0.007 |
| F11 | 0.506 | 0.336 | -0.592 | 0.006 | 0.020 |
| CDON | 1.438 | 0.954 | -0.592 | 0.000 | 0.000 |
| SDR16C5 | 23.400 | 15.498 | -0.594 | 0.000 | 0.001 |
| SMTNL2 | 0.665 | 0.440 | -0.595 | 0.003 | 0.012 |
| SEMA3E | 2.123 | 1.403 | -0.598 | 0.015 | 0.041 |
| NRXN3 | 0.684 | 0.452 | -0.598 | 0.000 | 0.000 |
| FASN | 53.592 | 35.321 | -0.601 | 0.000 | 0.000 |
| NAPSA | 546.621 | 360.010 | -0.603 | 0.000 | 0.000 |
| FOXA2 | 14.331 | 9.434 | -0.603 | 0.000 | 0.001 |
| HIGD1B | 3.927 | 2.585 | -0.603 | 0.000 | 0.001 |
| RPH3AL | 6.067 | 3.991 | -0.604 | 0.000 | 0.000 |
| LCN10 | 0.049 | 0.032 | -0.605 | 0.005 | 0.017 |
| CARMIL3 | 0.470 | 0.309 | -0.605 | 0.001 | 0.005 |
| DPP10 | 0.548 | 0.360 | -0.606 | 0.004 | 0.014 |
| AASS | 3.757 | 2.466 | -0.608 | 0.000 | 0.001 |
| ACADL | 0.996 | 0.652 | -0.611 | 0.011 | 0.032 |
| FREM2 | 1.460 | 0.956 | -0.611 | 0.004 | 0.014 |
| RELN | 0.563 | 0.368 | -0.612 | 0.014 | 0.039 |
| JPH1 | 3.848 | 2.517 | -0.612 | 0.000 | 0.000 |
| PLEKHG4B | 1.828 | 1.191 | -0.619 | 0.000 | 0.001 |
| TSNARE1 | 5.510 | 3.577 | -0.623 | 0.000 | 0.000 |
| ADGRF5 | 46.821 | 30.358 | -0.625 | 0.000 | 0.000 |
| TMEM108 | 1.896 | 1.229 | -0.625 | 0.000 | 0.000 |
| KLRG2 | 1.681 | 1.089 | -0.626 | 0.006 | 0.018 |
| VIPR1 | 2.794 | 1.810 | -0.626 | 0.000 | 0.000 |
| TCIM | 213.812 | 138.357 | -0.628 | 0.000 | 0.000 |
| GPC5 | 0.649 | 0.420 | -0.628 | 0.009 | 0.028 |
| OTUD7A | 0.125 | 0.080 | -0.631 | 0.000 | 0.000 |
| SORCS2 | 4.778 | 3.085 | -0.631 | 0.000 | 0.000 |
| TMEM163 | 7.069 | 4.561 | -0.632 | 0.000 | 0.000 |
| DAPK2 | 1.906 | 1.229 | -0.632 | 0.000 | 0.000 |
| CIT | 14.102 | 9.096 | -0.633 | 0.015 | 0.041 |
| SELENBP1 | 73.310 | 47.266 | -0.633 | 0.000 | 0.000 |
| C5 | 8.752 | 5.633 | -0.636 | 0.000 | 0.001 |
| UNC13B | 24.591 | 15.817 | -0.637 | 0.000 | 0.000 |
| PDK4 | 13.560 | 8.717 | -0.638 | 0.000 | 0.002 |
| SLC7A8 | 11.203 | 7.160 | -0.646 | 0.000 | 0.001 |
| RFTN1 | 19.210 | 12.270 | -0.647 | 0.000 | 0.000 |
| ANKRD65 | 16.242 | 10.372 | -0.647 | 0.000 | 0.000 |
| CATSPERB | 1.095 | 0.699 | -0.648 | 0.000 | 0.002 |
| TUBB1 | 0.196 | 0.125 | -0.648 | 0.014 | 0.038 |
| FBN3 | 0.643 | 0.409 | -0.653 | 0.008 | 0.025 |
| PRDM16 | 2.103 | 1.337 | -0.653 | 0.000 | 0.000 |
| ETNPPL | 0.700 | 0.445 | -0.654 | 0.000 | 0.002 |
| RND1 | 16.573 | 10.528 | -0.655 | 0.012 | 0.034 |
| ADARB2 | 0.123 | 0.078 | -0.658 | 0.003 | 0.011 |
| PDE4D | 4.997 | 3.164 | -0.659 | 0.000 | 0.000 |
| RAB3C | 0.153 | 0.097 | -0.660 | 0.012 | 0.033 |
| ZNF710 | 14.669 | 9.270 | -0.662 | 0.000 | 0.001 |
| EPHA3 | 1.514 | 0.955 | -0.664 | 0.004 | 0.015 |
| HMGB3 | 98.233 | 61.953 | -0.665 | 0.000 | 0.000 |
| PCDH15 | 0.051 | 0.032 | -0.668 | 0.010 | 0.030 |
| RXRG | 0.674 | 0.423 | -0.672 | 0.017 | 0.044 |
| DLC1 | 9.390 | 5.891 | -0.672 | 0.000 | 0.000 |
| PLA2G12B | 3.757 | 2.357 | -0.673 | 0.001 | 0.006 |
| ESYT3 | 2.573 | 1.610 | -0.676 | 0.000 | 0.000 |
| RHOBTB2 | 23.441 | 14.662 | -0.677 | 0.000 | 0.000 |
| PARM1 | 50.160 | 31.341 | -0.678 | 0.000 | 0.000 |
| C1orf127 | 0.536 | 0.334 | -0.680 | 0.000 | 0.001 |
| ALPL | 51.077 | 31.803 | -0.683 | 0.001 | 0.004 |
| ST6GALNAC4 | 21.508 | 13.369 | -0.686 | 0.000 | 0.000 |
| SYT7 | 15.480 | 9.613 | -0.687 | 0.000 | 0.000 |
| ATP8A1 | 6.462 | 4.008 | -0.689 | 0.000 | 0.000 |
| OTC | 0.067 | 0.042 | -0.691 | 0.007 | 0.022 |
| PDE3B | 1.787 | 1.101 | -0.699 | 0.000 | 0.000 |
| CHRD | 2.800 | 1.720 | -0.703 | 0.002 | 0.007 |
| PLCXD3 | 0.590 | 0.362 | -0.705 | 0.000 | 0.000 |
| BCL2 | 2.914 | 1.788 | -0.705 | 0.002 | 0.010 |
| KCNJ8 | 7.358 | 4.510 | -0.706 | 0.000 | 0.002 |
| ABCA8 | 1.048 | 0.640 | -0.711 | 0.001 | 0.004 |
| DAAM2 | 4.962 | 3.028 | -0.713 | 0.000 | 0.000 |
| CYB5A | 23.526 | 14.354 | -0.713 | 0.000 | 0.000 |
| CHRDL2 | 2.909 | 1.774 | -0.713 | 0.000 | 0.001 |
| POLR3H | 11.276 | 6.874 | -0.714 | 0.000 | 0.000 |
| SRARP | 0.187 | 0.114 | -0.717 | 0.000 | 0.000 |
| PLA2G4F | 3.148 | 1.914 | -0.718 | 0.000 | 0.000 |
| NEDD4L | 9.376 | 5.690 | -0.720 | 0.000 | 0.000 |
| RGMA | 1.088 | 0.658 | -0.725 | 0.003 | 0.012 |
| ACADSB | 8.598 | 5.194 | -0.727 | 0.000 | 0.000 |
| PLA2G4A | 26.797 | 16.163 | -0.729 | 0.001 | 0.004 |
| C12orf42 | 0.164 | 0.099 | -0.731 | 0.007 | 0.022 |
| SETBP1 | 1.664 | 1.002 | -0.732 | 0.000 | 0.000 |
| SHE | 4.865 | 2.928 | -0.732 | 0.000 | 0.000 |
| SCARA5 | 1.882 | 1.132 | -0.734 | 0.002 | 0.009 |
| FAM184B | 0.164 | 0.098 | -0.736 | 0.000 | 0.000 |
| ALDH3A2 | 38.710 | 23.232 | -0.737 | 0.000 | 0.000 |
| FGA | 130.257 | 78.109 | -0.738 | 0.018 | 0.047 |
| CNTN6 | 0.383 | 0.230 | -0.739 | 0.005 | 0.017 |
| CRHR2 | 0.386 | 0.231 | -0.739 | 0.000 | 0.001 |
| ADRB1 | 1.762 | 1.053 | -0.743 | 0.000 | 0.000 |
| TMED6 | 3.203 | 1.912 | -0.744 | 0.000 | 0.000 |
| KCNJ11 | 2.642 | 1.575 | -0.746 | 0.000 | 0.000 |
| SPINK14 | 0.472 | 0.281 | -0.746 | 0.002 | 0.008 |
| HUNK | 1.488 | 0.886 | -0.749 | 0.000 | 0.000 |
| SEMA6D | 0.933 | 0.555 | -0.749 | 0.000 | 0.001 |
| CACNA1D | 1.687 | 1.003 | -0.750 | 0.000 | 0.000 |
| RS1 | 0.146 | 0.087 | -0.752 | 0.001 | 0.006 |
| LGI3 | 1.472 | 0.874 | -0.752 | 0.013 | 0.035 |
| TFCP2L1 | 10.756 | 6.381 | -0.753 | 0.000 | 0.000 |
| LRRC52 | 0.376 | 0.223 | -0.755 | 0.005 | 0.017 |
| INSYN1 | 0.479 | 0.284 | -0.757 | 0.007 | 0.021 |
| CADM1 | 12.578 | 7.439 | -0.758 | 0.000 | 0.000 |
| CXXC4 | 1.961 | 1.149 | -0.771 | 0.000 | 0.001 |
| MAOA | 33.786 | 19.794 | -0.771 | 0.000 | 0.000 |
| ADH1B | 9.573 | 5.592 | -0.776 | 0.002 | 0.007 |
| CA8 | 1.653 | 0.965 | -0.777 | 0.002 | 0.007 |
| THPO | 1.769 | 1.032 | -0.778 | 0.016 | 0.043 |
| GADD45G | 19.776 | 11.517 | -0.780 | 0.000 | 0.000 |
| INMT | 10.673 | 6.211 | -0.781 | 0.000 | 0.000 |
| SFTA3 | 42.200 | 24.471 | -0.786 | 0.000 | 0.000 |
| KHDRBS3 | 2.227 | 1.287 | -0.791 | 0.000 | 0.000 |
| SLC22A31 | 60.709 | 34.998 | -0.795 | 0.000 | 0.000 |
| TCF7L1 | 8.525 | 4.912 | -0.796 | 0.000 | 0.000 |
| SLC16A14 | 10.331 | 5.924 | -0.802 | 0.009 | 0.027 |
| SCUBE2 | 3.951 | 2.261 | -0.805 | 0.000 | 0.001 |
| BTNL9 | 1.578 | 0.903 | -0.806 | 0.000 | 0.001 |
| AARD | 1.231 | 0.702 | -0.809 | 0.002 | 0.007 |
| ATP4B | 0.336 | 0.191 | -0.813 | 0.000 | 0.000 |
| TSPAN7 | 11.091 | 6.304 | -0.815 | 0.000 | 0.000 |
| ATOH8 | 7.217 | 4.101 | -0.815 | 0.000 | 0.000 |
| PNPLA7 | 1.778 | 1.009 | -0.816 | 0.000 | 0.001 |
| ACOXL | 1.221 | 0.690 | -0.822 | 0.000 | 0.000 |
| TRPC3 | 0.166 | 0.094 | -0.823 | 0.006 | 0.019 |
| ANKRD29 | 3.690 | 2.085 | -0.823 | 0.000 | 0.000 |
| RNF150 | 0.892 | 0.503 | -0.829 | 0.008 | 0.025 |
| MACROD2 | 2.825 | 1.585 | -0.834 | 0.003 | 0.012 |
| SH2D6 | 0.320 | 0.179 | -0.836 | 0.019 | 0.048 |
| C16orf89 | 155.073 | 86.530 | -0.842 | 0.000 | 0.000 |
| WIF1 | 21.437 | 11.943 | -0.844 | 0.000 | 0.000 |
| MSLN | 251.863 | 140.241 | -0.845 | 0.005 | 0.018 |
| OPRD1 | 0.324 | 0.179 | -0.852 | 0.017 | 0.044 |
| KLF15 | 3.853 | 2.134 | -0.852 | 0.000 | 0.000 |
| IRX2 | 13.811 | 7.621 | -0.858 | 0.000 | 0.000 |
| RORB | 0.395 | 0.217 | -0.863 | 0.000 | 0.000 |
| CPB2 | 5.323 | 2.920 | -0.866 | 0.006 | 0.020 |
| GFRA3 | 17.015 | 9.309 | -0.870 | 0.001 | 0.003 |
| GFRA1 | 0.779 | 0.426 | -0.871 | 0.001 | 0.004 |
| VEGFD | 3.113 | 1.698 | -0.874 | 0.000 | 0.003 |
| HES5 | 0.163 | 0.089 | -0.876 | 0.000 | 0.000 |
| IRX5 | 8.220 | 4.476 | -0.877 | 0.000 | 0.000 |
| SCNN1G | 6.244 | 3.399 | -0.877 | 0.000 | 0.000 |
| SFTPD | 215.943 | 116.883 | -0.886 | 0.000 | 0.001 |
| PAPPA | 0.817 | 0.442 | -0.886 | 0.000 | 0.000 |
| AQP4 | 19.450 | 10.520 | -0.887 | 0.000 | 0.000 |
| CNGA3 | 2.278 | 1.231 | -0.888 | 0.001 | 0.006 |
| NT5C1A | 0.357 | 0.192 | -0.894 | 0.001 | 0.005 |
| GDF10 | 1.861 | 0.998 | -0.899 | 0.005 | 0.016 |
| NCKAP5 | 0.837 | 0.448 | -0.902 | 0.001 | 0.006 |
| KCNK3 | 3.149 | 1.684 | -0.903 | 0.000 | 0.002 |
| KHDRBS2 | 1.353 | 0.720 | -0.909 | 0.000 | 0.000 |
| IRX3 | 25.475 | 13.547 | -0.911 | 0.000 | 0.000 |
| SOSTDC1 | 1.580 | 0.840 | -0.913 | 0.001 | 0.003 |
| NKD1 | 1.591 | 0.845 | -0.913 | 0.004 | 0.014 |
| ID4 | 10.497 | 5.571 | -0.914 | 0.000 | 0.000 |
| HOPX | 63.708 | 33.712 | -0.918 | 0.000 | 0.000 |
| AKR1C1 | 45.984 | 24.324 | -0.919 | 0.010 | 0.029 |
| SFTPA2 | 1746.192 | 922.904 | -0.920 | 0.000 | 0.000 |
| PROX1 | 1.456 | 0.766 | -0.926 | 0.000 | 0.001 |
| IGFBP2 | 59.022 | 30.949 | -0.931 | 0.000 | 0.000 |
| AFF3 | 0.827 | 0.433 | -0.932 | 0.000 | 0.000 |
| ATP6V1B1 | 2.954 | 1.544 | -0.936 | 0.003 | 0.012 |
| IRX1 | 1.771 | 0.923 | -0.941 | 0.001 | 0.004 |
| ZNF385B | 4.100 | 2.132 | -0.944 | 0.000 | 0.002 |
| ADGRB3 | 0.231 | 0.120 | -0.944 | 0.000 | 0.002 |
| EYS | 0.327 | 0.170 | -0.948 | 0.000 | 0.001 |
| SFTPA1 | 1453.499 | 752.770 | -0.949 | 0.000 | 0.000 |
| FGF9 | 0.870 | 0.449 | -0.952 | 0.001 | 0.005 |
| SFTPC | 455.760 | 234.781 | -0.957 | 0.010 | 0.029 |
| ZBTB16 | 1.262 | 0.649 | -0.958 | 0.000 | 0.000 |
| CLEC3B | 11.661 | 5.960 | -0.968 | 0.000 | 0.000 |
| ADGRV1 | 1.412 | 0.720 | -0.972 | 0.000 | 0.000 |
| EPHX1 | 220.246 | 111.994 | -0.976 | 0.000 | 0.000 |
| DDC | 5.566 | 2.829 | -0.976 | 0.016 | 0.043 |
| PAPPA2 | 0.197 | 0.100 | -0.977 | 0.007 | 0.021 |
| TAC4 | 2.542 | 1.278 | -0.992 | 0.001 | 0.004 |
| SEC11C | 33.652 | 16.865 | -0.997 | 0.000 | 0.000 |
| GRIA1 | 0.323 | 0.162 | -0.998 | 0.001 | 0.005 |
| PTN | 10.440 | 5.155 | -1.018 | 0.004 | 0.013 |
| PTCHD1 | 0.153 | 0.075 | -1.019 | 0.011 | 0.031 |
| CA5A | 0.051 | 0.025 | -1.022 | 0.018 | 0.046 |
| SLC7A2 | 21.722 | 10.637 | -1.030 | 0.000 | 0.000 |
| LRRC3B | 0.041 | 0.020 | -1.030 | 0.001 | 0.004 |
| PCP4 | 21.553 | 10.526 | -1.034 | 0.015 | 0.040 |
| KLB | 1.028 | 0.500 | -1.040 | 0.001 | 0.003 |
| ORM2 | 10.857 | 5.278 | -1.041 | 0.004 | 0.014 |
| EPHA7 | 0.440 | 0.213 | -1.046 | 0.012 | 0.033 |
| TSPAN11 | 6.448 | 3.111 | -1.052 | 0.001 | 0.003 |
| CDH15 | 1.500 | 0.722 | -1.056 | 0.000 | 0.000 |
| SYBU | 6.190 | 2.971 | -1.059 | 0.000 | 0.001 |
| CA4 | 1.394 | 0.669 | -1.060 | 0.003 | 0.012 |
| KIT | 19.328 | 9.223 | -1.067 | 0.000 | 0.000 |
| KL | 1.121 | 0.535 | -1.068 | 0.000 | 0.000 |
| ABCC8 | 0.249 | 0.118 | -1.072 | 0.000 | 0.003 |
| SYP | 1.322 | 0.628 | -1.074 | 0.000 | 0.000 |
| SLC47A2 | 0.161 | 0.076 | -1.085 | 0.000 | 0.000 |
| SUSD2 | 50.740 | 23.894 | -1.086 | 0.000 | 0.000 |
| CNTN4 | 0.623 | 0.291 | -1.099 | 0.000 | 0.000 |
| CYP4B1 | 42.615 | 19.838 | -1.103 | 0.000 | 0.001 |
| CD300LG | 0.209 | 0.097 | -1.104 | 0.003 | 0.011 |
| ADGRD1 | 3.679 | 1.700 | -1.114 | 0.000 | 0.000 |
| ERO1B | 7.898 | 3.623 | -1.124 | 0.000 | 0.000 |
| KCNK16 | 0.129 | 0.058 | -1.141 | 0.000 | 0.002 |
| LPL | 18.368 | 8.325 | -1.142 | 0.000 | 0.000 |
| ODC1 | 152.126 | 68.835 | -1.144 | 0.000 | 0.000 |
| GREB1 | 1.311 | 0.590 | -1.153 | 0.002 | 0.007 |
| OTULINL | 14.321 | 6.333 | -1.177 | 0.000 | 0.000 |
| TMEM132D | 0.595 | 0.262 | -1.185 | 0.000 | 0.000 |
| SLC46A2 | 3.527 | 1.533 | -1.202 | 0.000 | 0.000 |
| HSD17B13 | 1.915 | 0.828 | -1.211 | 0.001 | 0.006 |
| GRK1 | 0.220 | 0.093 | -1.246 | 0.001 | 0.004 |
| SFTPB | 3152.258 | 1329.054 | -1.246 | 0.000 | 0.000 |
| UMODL1 | 1.544 | 0.643 | -1.264 | 0.000 | 0.001 |
| P2RX2 | 0.321 | 0.133 | -1.266 | 0.017 | 0.045 |
| TAF1L | 0.045 | 0.019 | -1.276 | 0.004 | 0.013 |
| LRRC26 | 1.092 | 0.444 | -1.299 | 0.000 | 0.000 |
| PENK | 1.515 | 0.609 | -1.314 | 0.002 | 0.008 |
| ORM1 | 21.506 | 8.639 | -1.316 | 0.000 | 0.002 |
| BAALC | 4.271 | 1.690 | -1.338 | 0.000 | 0.000 |
| CTNND2 | 2.171 | 0.859 | -1.339 | 0.002 | 0.007 |
| ADCYAP1 | 0.672 | 0.259 | -1.374 | 0.001 | 0.004 |
| DSCAM | 0.101 | 0.039 | -1.387 | 0.001 | 0.006 |
| BMP6 | 8.037 | 3.050 | -1.398 | 0.000 | 0.000 |
| WNT4 | 2.583 | 0.973 | -1.409 | 0.000 | 0.000 |
| CHRM2 | 0.038 | 0.014 | -1.429 | 0.004 | 0.013 |
| CACNA2D2 | 15.255 | 5.658 | -1.431 | 0.000 | 0.000 |
| SLC16A12 | 1.283 | 0.470 | -1.448 | 0.003 | 0.013 |
| SALL3 | 0.205 | 0.074 | -1.468 | 0.006 | 0.020 |
| OCA2 | 0.558 | 0.200 | -1.478 | 0.000 | 0.000 |
| PEBP4 | 26.641 | 9.533 | -1.483 | 0.000 | 0.000 |
| FOXI3 | 1.794 | 0.637 | -1.494 | 0.010 | 0.029 |
| ENO3 | 5.368 | 1.903 | -1.496 | 0.000 | 0.000 |
| PAK3 | 0.924 | 0.320 | -1.527 | 0.000 | 0.000 |
| AGTR2 | 4.002 | 1.382 | -1.534 | 0.001 | 0.003 |
| GABRG3 | 0.114 | 0.039 | -1.543 | 0.006 | 0.020 |
| CHRNA2 | 0.028 | 0.009 | -1.611 | 0.017 | 0.045 |
| TMEM59L | 15.334 | 5.003 | -1.616 | 0.000 | 0.000 |
| TRIM71 | 0.300 | 0.096 | -1.640 | 0.014 | 0.038 |
| COL25A1 | 2.117 | 0.678 | -1.644 | 0.000 | 0.000 |
| BLOC1S5-TXNDC5 | 0.614 | 0.191 | -1.687 | 0.000 | 0.000 |
| CBR1 | 121.332 | 37.246 | -1.704 | 0.006 | 0.021 |
| SCGB3A2 | 552.671 | 165.828 | -1.737 | 0.000 | 0.000 |
| SLC38A3 | 0.926 | 0.270 | -1.776 | 0.015 | 0.040 |
| CRLF1 | 75.215 | 21.876 | -1.782 | 0.000 | 0.000 |
| STUM | 2.002 | 0.579 | -1.789 | 0.000 | 0.000 |
| TENM1 | 1.346 | 0.386 | -1.802 | 0.000 | 0.001 |
| PAX7 | 3.076 | 0.873 | -1.817 | 0.000 | 0.000 |
| SCGB3A1 | 847.080 | 238.313 | -1.830 | 0.017 | 0.044 |
| KCNA4 | 0.110 | 0.031 | -1.841 | 0.000 | 0.001 |
| MTMR7 | 2.302 | 0.601 | -1.937 | 0.000 | 0.002 |
| MYT1 | 0.683 | 0.175 | -1.962 | 0.000 | 0.000 |
| PKHD1L1 | 0.310 | 0.079 | -1.965 | 0.005 | 0.017 |
| C10orf71 | 0.138 | 0.035 | -1.974 | 0.000 | 0.000 |
| NPAS3 | 0.913 | 0.230 | -1.989 | 0.004 | 0.015 |
| FGL1 | 30.116 | 7.408 | -2.023 | 0.000 | 0.000 |
| BPIFB2 | 23.636 | 5.772 | -2.034 | 0.006 | 0.019 |
| NHLH2 | 0.186 | 0.045 | -2.034 | 0.001 | 0.003 |
| TMEM229A | 1.500 | 0.361 | -2.055 | 0.000 | 0.000 |
| PCSK2 | 19.320 | 4.614 | -2.066 | 0.000 | 0.000 |
| ASCL1 | 26.798 | 6.187 | -2.115 | 0.003 | 0.010 |
| RET | 3.545 | 0.782 | -2.181 | 0.001 | 0.003 |
| GKN2 | 15.974 | 3.188 | -2.325 | 0.000 | 0.000 |
| SYT4 | 1.286 | 0.248 | -2.372 | 0.001 | 0.004 |
| PGC | 1347.461 | 226.896 | -2.570 | 0.000 | 0.000 |
| CA6 | 0.516 | 0.068 | -2.919 | 0.000 | 0.001 |
| SLC14A2 | 2.539 | 0.335 | -2.923 | 0.000 | 0.000 |
| GNRH2 | 1.312 | 0.139 | -3.235 | 0.001 | 0.003 |
| CALCA | 233.805 | 7.315 | -4.998 | 0.002 | 0.007 |

Table S2. The DEGs between the high-NGEF expression group and the low-NGEF expression group in GSE31210

| gene | lowMean | highMean | logFC | abs(logFC) | pValue | fdr |
| --- | --- | --- | --- | --- | --- | --- |
| CKM | 6.447 | 29.821 | 2.210 | 2.210 | 0.000 | 0.001 |
| TMPRSS11E | 59.645 | 250.318 | 2.069 | 2.069 | 0.001 | 0.005 |
| PRAME | 36.135 | 141.176 | 1.966 | 1.966 | 0.001 | 0.008 |
| ARL14 | 59.364 | 229.646 | 1.952 | 1.952 | 0.001 | 0.007 |
| POU6F2-AS2 | 30.277 | 115.400 | 1.930 | 1.930 | 0.000 | 0.000 |
| CEACAM7 | 98.759 | 374.293 | 1.922 | 1.922 | 0.004 | 0.018 |
| MUC13 | 101.298 | 373.762 | 1.884 | 1.884 | 0.000 | 0.001 |
| MUC5AC | 67.318 | 242.220 | 1.847 | 1.847 | 0.002 | 0.011 |
| ERVH48-1 | 5.441 | 19.569 | 1.847 | 1.847 | 0.002 | 0.012 |
| CHGB | 143.130 | 514.450 | 1.846 | 1.846 | 0.005 | 0.021 |
| VTCN1 | 150.517 | 519.837 | 1.788 | 1.788 | 0.000 | 0.000 |
| NGEF | 48.236 | 166.308 | 1.786 | 1.786 | 0.000 | 0.000 |
| AOC1 | 120.180 | 414.282 | 1.785 | 1.785 | 0.000 | 0.000 |
| KRT20 | 68.405 | 233.784 | 1.773 | 1.773 | 0.005 | 0.021 |
| AKR1B10 | 375.882 | 1263.722 | 1.749 | 1.749 | 0.000 | 0.003 |
| FLJ13744 | 154.287 | 515.564 | 1.741 | 1.741 | 0.000 | 0.000 |
| TRPM8 | 74.097 | 238.372 | 1.686 | 1.686 | 0.000 | 0.001 |
| MUC16 | 96.482 | 307.735 | 1.673 | 1.673 | 0.000 | 0.000 |
| IBSP | 47.750 | 149.956 | 1.651 | 1.651 | 0.000 | 0.000 |
| CYP26A1 | 78.020 | 242.475 | 1.636 | 1.636 | 0.011 | 0.038 |
| KLK6 | 52.621 | 162.357 | 1.625 | 1.625 | 0.003 | 0.016 |
| GP2 | 15.586 | 47.007 | 1.593 | 1.593 | 0.003 | 0.014 |
| SYT13 | 116.765 | 349.580 | 1.582 | 1.582 | 0.001 | 0.008 |
| TRIM31 | 23.086 | 68.338 | 1.566 | 1.566 | 0.014 | 0.047 |
| PTPRH | 60.690 | 173.342 | 1.514 | 1.514 | 0.000 | 0.000 |
| GPR87 | 322.528 | 896.492 | 1.475 | 1.475 | 0.000 | 0.000 |
| CEACAM5 | 1803.809 | 4986.757 | 1.467 | 1.467 | 0.000 | 0.000 |
| MUC17 | 43.063 | 117.121 | 1.443 | 1.443 | 0.004 | 0.019 |
| ZIC2 | 64.893 | 173.016 | 1.415 | 1.415 | 0.000 | 0.003 |
| HTR3A | 53.989 | 143.436 | 1.410 | 1.410 | 0.000 | 0.000 |
| GALNT14 | 163.960 | 428.884 | 1.387 | 1.387 | 0.000 | 0.000 |
| MAP7D2 | 324.336 | 834.277 | 1.363 | 1.363 | 0.000 | 0.000 |
| ANXA10 | 285.460 | 732.744 | 1.360 | 1.360 | 0.002 | 0.010 |
| TCN1 | 407.522 | 1039.325 | 1.351 | 1.351 | 0.000 | 0.000 |
| GCNT3 | 959.057 | 2441.579 | 1.348 | 1.348 | 0.000 | 0.000 |
| LECT1 | 120.639 | 302.854 | 1.328 | 1.328 | 0.000 | 0.004 |
| TFAP2D | 43.753 | 108.525 | 1.311 | 1.311 | 0.002 | 0.011 |
| SLC1A6 | 6.997 | 17.246 | 1.301 | 1.301 | 0.000 | 0.001 |
| DNER | 185.443 | 453.419 | 1.290 | 1.290 | 0.006 | 0.023 |
| ANGPTL4 | 123.594 | 299.533 | 1.277 | 1.277 | 0.000 | 0.001 |
| MEGF10 | 40.840 | 98.892 | 1.276 | 1.276 | 0.002 | 0.012 |
| LY6K | 79.059 | 191.159 | 1.274 | 1.274 | 0.000 | 0.001 |
| PLAC1 | 20.100 | 48.313 | 1.265 | 1.265 | 0.000 | 0.000 |
| ITPKA | 48.965 | 117.301 | 1.260 | 1.260 | 0.000 | 0.000 |
| IL37 | 90.985 | 217.910 | 1.260 | 1.260 | 0.000 | 0.003 |
| PITX1 | 12.850 | 30.658 | 1.255 | 1.255 | 0.000 | 0.001 |
| S100P | 1059.076 | 2518.806 | 1.250 | 1.250 | 0.000 | 0.004 |
| TMPRSS4 | 288.506 | 684.699 | 1.247 | 1.247 | 0.000 | 0.000 |
| PRR15 | 114.028 | 269.425 | 1.240 | 1.240 | 0.000 | 0.000 |
| VIL1 | 62.659 | 145.965 | 1.220 | 1.220 | 0.008 | 0.030 |
| MB | 222.617 | 517.304 | 1.216 | 1.216 | 0.000 | 0.000 |
| TFF1 | 663.656 | 1541.233 | 1.216 | 1.216 | 0.012 | 0.041 |
| BRINP1 | 57.226 | 132.020 | 1.206 | 1.206 | 0.010 | 0.035 |
| PPP1R14D | 127.886 | 294.282 | 1.202 | 1.202 | 0.000 | 0.000 |
| LY6D | 57.897 | 132.931 | 1.199 | 1.199 | 0.002 | 0.012 |
| S100A2 | 720.278 | 1634.401 | 1.182 | 1.182 | 0.000 | 0.002 |
| COL11A1 | 1480.597 | 3329.981 | 1.169 | 1.169 | 0.000 | 0.000 |
| TFF3 | 611.077 | 1370.048 | 1.165 | 1.165 | 0.011 | 0.038 |
| ATP10B | 171.745 | 383.270 | 1.158 | 1.158 | 0.000 | 0.001 |
| LINC01207 | 184.158 | 409.484 | 1.153 | 1.153 | 0.000 | 0.000 |
| GALNT6 | 152.896 | 338.602 | 1.147 | 1.147 | 0.000 | 0.000 |
| C15orf48 | 2113.229 | 4505.070 | 1.092 | 1.092 | 0.000 | 0.000 |
| ALDH3B2 | 39.583 | 83.829 | 1.083 | 1.083 | 0.000 | 0.000 |
| CLDN10-AS1 | 27.651 | 58.476 | 1.081 | 1.081 | 0.000 | 0.003 |
| ABCA12 | 42.926 | 90.466 | 1.076 | 1.076 | 0.000 | 0.000 |
| QPCT | 527.161 | 1106.900 | 1.070 | 1.070 | 0.004 | 0.017 |
| PKIB | 283.705 | 594.717 | 1.068 | 1.068 | 0.000 | 0.000 |
| AC024560.2 | 144.558 | 300.901 | 1.058 | 1.058 | 0.000 | 0.000 |
| LYPD1 | 75.202 | 156.149 | 1.054 | 1.054 | 0.000 | 0.000 |
| KRT15 | 544.226 | 1128.547 | 1.052 | 1.052 | 0.000 | 0.000 |
| MMP13 | 322.441 | 666.912 | 1.048 | 1.048 | 0.000 | 0.000 |
| SPP2 | 51.390 | 106.265 | 1.048 | 1.048 | 0.000 | 0.003 |
| KRTAP4-1 | 44.268 | 91.055 | 1.040 | 1.040 | 0.000 | 0.002 |
| SERPINA5 | 36.681 | 74.685 | 1.026 | 1.026 | 0.004 | 0.018 |
| SRPX2 | 289.046 | 587.905 | 1.024 | 1.024 | 0.000 | 0.000 |
| SPRR1B | 188.683 | 383.012 | 1.021 | 1.021 | 0.014 | 0.046 |
| SOX11 | 74.620 | 150.297 | 1.010 | 1.010 | 0.014 | 0.045 |
| MMP11 | 250.234 | 503.581 | 1.009 | 1.009 | 0.000 | 0.000 |
| KRT75 | 23.983 | 48.182 | 1.006 | 1.006 | 0.001 | 0.007 |
| SPINK1 | 1479.013 | 2949.189 | 0.996 | 0.996 | 0.000 | 0.000 |
| BEAN1 | 106.688 | 212.627 | 0.995 | 0.995 | 0.000 | 0.000 |
| CST6 | 266.394 | 528.796 | 0.989 | 0.989 | 0.000 | 0.003 |
| MUC20 | 243.374 | 481.726 | 0.985 | 0.985 | 0.000 | 0.000 |
| LYPD3 | 74.799 | 147.916 | 0.984 | 0.984 | 0.000 | 0.002 |
| EGLN3 | 432.274 | 853.870 | 0.982 | 0.982 | 0.000 | 0.000 |
| NKX3-2 | 63.322 | 123.800 | 0.967 | 0.967 | 0.000 | 0.001 |
| CHST6 | 80.339 | 156.582 | 0.963 | 0.963 | 0.000 | 0.000 |
| SLC6A8 | 164.003 | 319.200 | 0.961 | 0.961 | 0.000 | 0.000 |
| GPR37 | 67.577 | 130.910 | 0.954 | 0.954 | 0.000 | 0.000 |
| MYBL2 | 112.252 | 215.373 | 0.940 | 0.940 | 0.000 | 0.000 |
| FAM83A | 190.959 | 365.939 | 0.938 | 0.938 | 0.000 | 0.000 |
| CXCL6 | 381.307 | 728.034 | 0.933 | 0.933 | 0.006 | 0.024 |
| IGSF9 | 82.806 | 157.989 | 0.932 | 0.932 | 0.000 | 0.000 |
| SLC4A11 | 107.075 | 204.069 | 0.930 | 0.930 | 0.000 | 0.000 |
| DIO1 | 137.456 | 261.836 | 0.930 | 0.930 | 0.003 | 0.014 |
| B3GNT3 | 128.688 | 242.235 | 0.913 | 0.913 | 0.000 | 0.000 |
| FGFBP1 | 157.744 | 296.812 | 0.912 | 0.912 | 0.000 | 0.003 |
| LINC00460 | 58.237 | 109.538 | 0.911 | 0.911 | 0.000 | 0.002 |
| HNF4G | 72.712 | 136.626 | 0.910 | 0.910 | 0.000 | 0.000 |
| HMGA2 | 48.535 | 91.125 | 0.909 | 0.909 | 0.004 | 0.018 |
| CDC45 | 56.527 | 105.903 | 0.906 | 0.906 | 0.000 | 0.001 |
| ZYG11A | 30.873 | 57.668 | 0.901 | 0.901 | 0.000 | 0.001 |
| TSPAN1 | 458.959 | 855.322 | 0.898 | 0.898 | 0.000 | 0.001 |
| NMU | 218.351 | 406.528 | 0.897 | 0.897 | 0.000 | 0.001 |
| LOC283112 | 8.031 | 14.950 | 0.896 | 0.896 | 0.007 | 0.028 |
| HS3ST1 | 239.033 | 444.551 | 0.895 | 0.895 | 0.000 | 0.000 |
| NETO1 | 29.871 | 55.529 | 0.895 | 0.895 | 0.000 | 0.000 |
| ANLN | 437.942 | 810.218 | 0.888 | 0.888 | 0.000 | 0.000 |
| SEMA3A | 83.457 | 154.395 | 0.888 | 0.888 | 0.012 | 0.041 |
| COMP | 869.207 | 1606.815 | 0.886 | 0.886 | 0.000 | 0.001 |
| STEAP1 | 1123.090 | 2073.320 | 0.884 | 0.884 | 0.000 | 0.000 |
| KISS1R | 209.775 | 387.093 | 0.884 | 0.884 | 0.000 | 0.003 |
| RHPN2 | 805.839 | 1484.726 | 0.882 | 0.882 | 0.000 | 0.000 |
| RGS17 | 269.929 | 495.153 | 0.875 | 0.875 | 0.000 | 0.001 |
| POF1B | 54.890 | 100.584 | 0.874 | 0.874 | 0.000 | 0.001 |
| KCNN4 | 188.326 | 344.616 | 0.872 | 0.872 | 0.000 | 0.000 |
| TRIP13 | 368.436 | 673.368 | 0.870 | 0.870 | 0.000 | 0.000 |
| KRT8 | 1643.020 | 3002.391 | 0.870 | 0.870 | 0.000 | 0.000 |
| CXCL14 | 1440.616 | 2629.383 | 0.868 | 0.868 | 0.000 | 0.001 |
| IYD | 141.121 | 256.728 | 0.863 | 0.863 | 0.001 | 0.005 |
| ECM1 | 397.642 | 722.331 | 0.861 | 0.861 | 0.002 | 0.010 |
| PPP2R2C | 33.402 | 60.594 | 0.859 | 0.859 | 0.000 | 0.001 |
| UHRF1 | 315.942 | 571.629 | 0.855 | 0.855 | 0.000 | 0.000 |
| TFAP2A | 179.284 | 324.251 | 0.855 | 0.855 | 0.000 | 0.000 |
| GRB7 | 165.640 | 299.542 | 0.855 | 0.855 | 0.000 | 0.001 |
| MFI2 | 169.918 | 306.994 | 0.853 | 0.853 | 0.000 | 0.000 |
| DOCK3 | 28.948 | 52.271 | 0.853 | 0.853 | 0.004 | 0.018 |
| LINC01133 | 41.579 | 75.066 | 0.852 | 0.852 | 0.002 | 0.011 |
| WFDC3 | 208.255 | 375.917 | 0.852 | 0.852 | 0.000 | 0.000 |
| GJB2 | 656.172 | 1184.046 | 0.852 | 0.852 | 0.000 | 0.002 |
| LOC285556 | 31.560 | 56.767 | 0.847 | 0.847 | 0.010 | 0.035 |
| ANKS4B | 9.610 | 17.275 | 0.846 | 0.846 | 0.008 | 0.029 |
| CA9 | 155.141 | 278.594 | 0.845 | 0.845 | 0.002 | 0.011 |
| OGDHL | 11.326 | 20.286 | 0.841 | 0.841 | 0.011 | 0.038 |
| CLDN3 | 330.911 | 591.551 | 0.838 | 0.838 | 0.000 | 0.000 |
| MUC4 | 237.060 | 422.805 | 0.835 | 0.835 | 0.006 | 0.023 |
| CP | 2255.808 | 4019.507 | 0.833 | 0.833 | 0.000 | 0.000 |
| AHNAK2 | 488.285 | 865.934 | 0.827 | 0.827 | 0.001 | 0.004 |
| ENPP3 | 396.308 | 702.638 | 0.826 | 0.826 | 0.009 | 0.034 |
| RP11-1L12.3 | 53.425 | 94.679 | 0.826 | 0.826 | 0.000 | 0.001 |
| SPINK13 | 85.022 | 150.661 | 0.825 | 0.825 | 0.002 | 0.010 |
| EXO1 | 44.587 | 78.967 | 0.825 | 0.825 | 0.000 | 0.002 |
| KIF4A | 214.679 | 379.659 | 0.823 | 0.823 | 0.000 | 0.000 |
| RP11-486A14.1 | 18.289 | 32.317 | 0.821 | 0.821 | 0.000 | 0.002 |
| SLC24A2 | 43.405 | 76.670 | 0.821 | 0.821 | 0.000 | 0.003 |
| C11orf86 | 24.243 | 42.748 | 0.818 | 0.818 | 0.000 | 0.000 |
| CST4 | 295.673 | 520.672 | 0.816 | 0.816 | 0.000 | 0.000 |
| SLC7A11 | 362.330 | 637.760 | 0.816 | 0.816 | 0.004 | 0.018 |
| PPP1R1B | 295.533 | 519.504 | 0.814 | 0.814 | 0.007 | 0.027 |
| MELK | 841.809 | 1479.496 | 0.814 | 0.814 | 0.000 | 0.000 |
| RHOV | 62.743 | 110.028 | 0.810 | 0.810 | 0.000 | 0.000 |
| RNF186 | 38.421 | 67.168 | 0.806 | 0.806 | 0.002 | 0.010 |
| NEK2 | 136.448 | 237.844 | 0.802 | 0.802 | 0.000 | 0.000 |
| NPTX2 | 141.093 | 245.340 | 0.798 | 0.798 | 0.005 | 0.021 |
| IGFBPL1 | 178.931 | 310.963 | 0.797 | 0.797 | 0.000 | 0.000 |
| CST1 | 113.951 | 197.995 | 0.797 | 0.797 | 0.000 | 0.000 |
| PBK | 243.748 | 423.410 | 0.797 | 0.797 | 0.000 | 0.000 |
| CEP55 | 380.150 | 659.842 | 0.796 | 0.796 | 0.000 | 0.000 |
| CPNE4 | 77.038 | 133.646 | 0.795 | 0.795 | 0.000 | 0.001 |
| TRIM29 | 141.290 | 244.687 | 0.792 | 0.792 | 0.014 | 0.047 |
| FRMD5 | 136.271 | 235.978 | 0.792 | 0.792 | 0.000 | 0.000 |
| ITGB8 | 471.428 | 815.288 | 0.790 | 0.790 | 0.000 | 0.000 |
| IL36RN | 27.946 | 48.303 | 0.790 | 0.790 | 0.000 | 0.002 |
| HOXC13 | 15.449 | 26.702 | 0.789 | 0.789 | 0.000 | 0.002 |
| RP1-35C21.2 | 15.722 | 27.161 | 0.789 | 0.789 | 0.001 | 0.005 |
| VSTM2L | 182.391 | 314.871 | 0.788 | 0.788 | 0.000 | 0.001 |
| EPHX4 | 104.482 | 180.290 | 0.787 | 0.787 | 0.009 | 0.032 |
| TMEM61 | 84.308 | 145.300 | 0.785 | 0.785 | 0.000 | 0.001 |
| IGFL2 | 77.579 | 133.475 | 0.783 | 0.783 | 0.000 | 0.000 |
| FUT3 | 96.879 | 166.551 | 0.782 | 0.782 | 0.000 | 0.000 |
| OIP5 | 102.571 | 176.290 | 0.781 | 0.781 | 0.000 | 0.001 |
| KRT19 | 2005.076 | 3442.983 | 0.780 | 0.780 | 0.000 | 0.000 |
| HOXB13 | 7.190 | 12.317 | 0.777 | 0.777 | 0.001 | 0.008 |
| MNX1-AS1 | 3.819 | 6.540 | 0.776 | 0.776 | 0.000 | 0.003 |
| RAB3B | 21.795 | 37.254 | 0.773 | 0.773 | 0.001 | 0.007 |
| PKP2 | 87.967 | 150.086 | 0.771 | 0.771 | 0.011 | 0.038 |
| LOC101927934 | 22.364 | 38.146 | 0.770 | 0.770 | 0.000 | 0.001 |
| PPAPDC1A | 136.055 | 231.974 | 0.770 | 0.770 | 0.000 | 0.000 |
| SULT1C2 | 426.797 | 727.564 | 0.770 | 0.770 | 0.002 | 0.011 |
| BIRC5 | 111.664 | 190.155 | 0.768 | 0.768 | 0.000 | 0.000 |
| GAD1 | 73.246 | 124.458 | 0.765 | 0.765 | 0.000 | 0.000 |
| CDCA3 | 166.006 | 281.411 | 0.761 | 0.761 | 0.000 | 0.000 |
| ERCC6L | 74.809 | 126.799 | 0.761 | 0.761 | 0.000 | 0.000 |
| LOC100505938 | 114.437 | 193.795 | 0.760 | 0.760 | 0.000 | 0.000 |
| DIO2 | 242.726 | 410.489 | 0.758 | 0.758 | 0.000 | 0.001 |
| ENPP1 | 67.536 | 114.132 | 0.757 | 0.757 | 0.000 | 0.001 |
| FA2H | 181.430 | 306.552 | 0.757 | 0.757 | 0.000 | 0.000 |
| FNDC1 | 761.821 | 1286.807 | 0.756 | 0.756 | 0.001 | 0.007 |
| SRD5A3 | 659.004 | 1112.741 | 0.756 | 0.756 | 0.000 | 0.000 |
| SOWAHA | 44.085 | 74.327 | 0.754 | 0.754 | 0.005 | 0.020 |
| NUP62CL | 45.503 | 76.513 | 0.750 | 0.750 | 0.000 | 0.000 |
| SEMA4B | 1512.828 | 2539.487 | 0.747 | 0.747 | 0.000 | 0.000 |
| LRRC15 | 426.651 | 715.947 | 0.747 | 0.747 | 0.001 | 0.006 |
| DEPDC1B | 148.814 | 249.589 | 0.746 | 0.746 | 0.000 | 0.000 |
| BUB1B | 518.863 | 870.043 | 0.746 | 0.746 | 0.000 | 0.000 |
| HS6ST2 | 1919.237 | 3215.775 | 0.745 | 0.745 | 0.000 | 0.000 |
| TMEM45B | 897.028 | 1502.351 | 0.744 | 0.744 | 0.000 | 0.000 |
| TNFSF11 | 41.545 | 69.431 | 0.741 | 0.741 | 0.001 | 0.006 |
| SLC2A1 | 285.217 | 476.341 | 0.740 | 0.740 | 0.000 | 0.000 |
| ST14 | 674.835 | 1126.962 | 0.740 | 0.740 | 0.000 | 0.000 |
| BIK | 184.429 | 307.965 | 0.740 | 0.740 | 0.000 | 0.001 |
| GPR115 | 32.229 | 53.809 | 0.739 | 0.739 | 0.000 | 0.000 |
| ANXA9 | 41.786 | 69.699 | 0.738 | 0.738 | 0.001 | 0.005 |
| TPX2 | 279.507 | 466.063 | 0.738 | 0.738 | 0.000 | 0.000 |
| SPC25 | 66.996 | 111.609 | 0.736 | 0.736 | 0.000 | 0.001 |
| KRT80 | 373.655 | 622.412 | 0.736 | 0.736 | 0.000 | 0.000 |
| EPYC | 124.962 | 208.104 | 0.736 | 0.736 | 0.015 | 0.049 |
| HHLA2 | 61.819 | 102.864 | 0.735 | 0.735 | 0.000 | 0.001 |
| ONECUT1 | 17.228 | 28.651 | 0.734 | 0.734 | 0.000 | 0.000 |
| TWIST1 | 239.867 | 398.588 | 0.733 | 0.733 | 0.010 | 0.037 |
| HIST1H2AE | 44.116 | 73.245 | 0.731 | 0.731 | 0.000 | 0.004 |
| CDH3 | 969.917 | 1608.437 | 0.730 | 0.730 | 0.000 | 0.000 |
| SLC51A | 31.833 | 52.728 | 0.728 | 0.728 | 0.000 | 0.002 |
| SLC44A5 | 106.134 | 175.605 | 0.726 | 0.726 | 0.000 | 0.001 |
| CCDC160 | 43.634 | 72.108 | 0.725 | 0.725 | 0.004 | 0.018 |
| PRC1 | 485.130 | 801.459 | 0.724 | 0.724 | 0.000 | 0.000 |
| MCM10 | 91.409 | 150.823 | 0.722 | 0.722 | 0.000 | 0.000 |
| FUT9 | 38.323 | 63.122 | 0.720 | 0.720 | 0.004 | 0.017 |
| CDIPT-AS1 | 22.511 | 37.068 | 0.720 | 0.720 | 0.000 | 0.002 |
| TTK | 200.139 | 329.459 | 0.719 | 0.719 | 0.000 | 0.000 |
| FOXM1 | 149.354 | 245.314 | 0.716 | 0.716 | 0.000 | 0.000 |
| AFAP1-AS1 | 842.749 | 1382.733 | 0.714 | 0.714 | 0.000 | 0.000 |
| GOLM1 | 1490.580 | 2444.608 | 0.714 | 0.714 | 0.000 | 0.000 |
| C20orf197 | 86.889 | 142.395 | 0.713 | 0.713 | 0.000 | 0.002 |
| ALOX12P2 | 25.982 | 42.571 | 0.712 | 0.712 | 0.001 | 0.004 |
| UBE2T | 188.717 | 308.758 | 0.710 | 0.710 | 0.000 | 0.000 |
| F12 | 58.566 | 95.778 | 0.710 | 0.710 | 0.000 | 0.000 |
| KIF14 | 165.774 | 270.924 | 0.709 | 0.709 | 0.000 | 0.000 |
| PLA2G4A | 974.034 | 1591.566 | 0.708 | 0.708 | 0.003 | 0.013 |
| IL36G | 32.810 | 53.594 | 0.708 | 0.708 | 0.001 | 0.005 |
| STYK1 | 260.729 | 425.452 | 0.706 | 0.706 | 0.000 | 0.000 |
| UFSP1 | 30.019 | 48.883 | 0.703 | 0.703 | 0.000 | 0.001 |
| IGF2BP3 | 421.547 | 685.507 | 0.701 | 0.701 | 0.000 | 0.003 |
| MIOX | 26.678 | 43.335 | 0.700 | 0.700 | 0.010 | 0.036 |
| COL10A1 | 2557.062 | 4150.233 | 0.699 | 0.699 | 0.000 | 0.000 |
| PROC | 42.053 | 68.163 | 0.697 | 0.697 | 0.000 | 0.000 |
| LINC00326 | 47.814 | 77.394 | 0.695 | 0.695 | 0.000 | 0.004 |
| LINC00857 | 59.224 | 95.838 | 0.694 | 0.694 | 0.000 | 0.001 |
| TOP2A | 531.831 | 860.093 | 0.694 | 0.694 | 0.000 | 0.000 |
| PRRX2 | 44.643 | 72.151 | 0.693 | 0.693 | 0.005 | 0.022 |
| TGFA | 309.219 | 499.727 | 0.693 | 0.693 | 0.000 | 0.000 |
| P2RY6 | 163.770 | 264.604 | 0.692 | 0.692 | 0.000 | 0.000 |
| FBXL19 | 16.339 | 26.353 | 0.690 | 0.690 | 0.000 | 0.000 |
| CRABP2 | 743.366 | 1195.632 | 0.686 | 0.686 | 0.000 | 0.001 |
| EPHX3 | 178.194 | 286.388 | 0.685 | 0.685 | 0.000 | 0.000 |
| SLC8A2 | 7.336 | 11.791 | 0.684 | 0.684 | 0.005 | 0.021 |
| SPP1 | 3029.987 | 4868.768 | 0.684 | 0.684 | 0.000 | 0.000 |
| FOXA3 | 248.293 | 398.852 | 0.684 | 0.684 | 0.000 | 0.002 |
| UBE2C | 406.855 | 653.484 | 0.684 | 0.684 | 0.000 | 0.000 |
| DNAJC12 | 337.921 | 542.163 | 0.682 | 0.682 | 0.000 | 0.002 |
| MMP12 | 1776.551 | 2849.224 | 0.681 | 0.681 | 0.001 | 0.007 |
| DSP | 4384.858 | 7023.049 | 0.680 | 0.680 | 0.000 | 0.000 |
| HJURP | 130.087 | 208.204 | 0.679 | 0.679 | 0.000 | 0.000 |
| HLA-DPB2 | 221.702 | 354.810 | 0.678 | 0.678 | 0.000 | 0.001 |
| ANKRD36C | 96.105 | 153.468 | 0.675 | 0.675 | 0.001 | 0.004 |
| SIX1 | 350.048 | 558.894 | 0.675 | 0.675 | 0.000 | 0.001 |
| RRM2 | 1552.477 | 2476.080 | 0.673 | 0.673 | 0.000 | 0.000 |
| GRIK2 | 27.210 | 43.387 | 0.673 | 0.673 | 0.000 | 0.001 |
| GRHL3 | 41.247 | 65.760 | 0.673 | 0.673 | 0.001 | 0.005 |
| KIAA0101 | 995.346 | 1586.880 | 0.673 | 0.673 | 0.000 | 0.000 |
| MIR646HG | 128.345 | 204.328 | 0.671 | 0.671 | 0.006 | 0.023 |
| GINS1 | 565.235 | 899.310 | 0.670 | 0.670 | 0.000 | 0.000 |
| CCDC37-AS1 | 16.902 | 26.872 | 0.669 | 0.669 | 0.015 | 0.049 |
| C9orf84 | 63.192 | 100.346 | 0.667 | 0.667 | 0.001 | 0.006 |
| CREG2 | 14.702 | 23.345 | 0.667 | 0.667 | 0.001 | 0.007 |
| CDCA8 | 202.074 | 320.803 | 0.667 | 0.667 | 0.000 | 0.000 |
| NUF2 | 283.811 | 450.447 | 0.666 | 0.666 | 0.000 | 0.000 |
| CENPF | 225.342 | 357.378 | 0.665 | 0.665 | 0.000 | 0.000 |
| LCN2 | 1207.331 | 1910.653 | 0.662 | 0.662 | 0.014 | 0.046 |
| PPAP2C | 535.581 | 847.413 | 0.662 | 0.662 | 0.000 | 0.000 |
| ASPM | 201.834 | 319.268 | 0.662 | 0.662 | 0.000 | 0.000 |
| NDC80 | 310.658 | 491.400 | 0.662 | 0.662 | 0.000 | 0.001 |
| KLHDC8A | 67.276 | 106.398 | 0.661 | 0.661 | 0.002 | 0.009 |
| HOXB7 | 374.947 | 592.516 | 0.660 | 0.660 | 0.000 | 0.001 |
| CNFN | 25.428 | 40.109 | 0.658 | 0.658 | 0.000 | 0.001 |
| NEIL3 | 110.893 | 174.887 | 0.657 | 0.657 | 0.000 | 0.000 |
| C16orf59 | 51.501 | 81.203 | 0.657 | 0.657 | 0.000 | 0.000 |
| C1orf200 | 13.625 | 21.480 | 0.657 | 0.657 | 0.000 | 0.000 |
| BLACAT1 | 146.554 | 231.019 | 0.657 | 0.657 | 0.000 | 0.000 |
| IDO1 | 1060.986 | 1671.585 | 0.656 | 0.656 | 0.012 | 0.042 |
| IQGAP3 | 90.841 | 143.033 | 0.655 | 0.655 | 0.000 | 0.000 |
| SLC22A18 | 819.799 | 1290.080 | 0.654 | 0.654 | 0.000 | 0.000 |
| SMIM22 | 120.370 | 189.204 | 0.652 | 0.652 | 0.000 | 0.000 |
| PCSK1N | 143.499 | 225.240 | 0.650 | 0.650 | 0.000 | 0.002 |
| BCL2L15 | 222.927 | 349.641 | 0.649 | 0.649 | 0.000 | 0.000 |
| BEST4 | 41.604 | 65.242 | 0.649 | 0.649 | 0.000 | 0.002 |
| SIGLEC15 | 91.453 | 143.410 | 0.649 | 0.649 | 0.000 | 0.001 |
| SMIM5 | 103.927 | 162.938 | 0.649 | 0.649 | 0.000 | 0.000 |
| HOXC9 | 51.145 | 80.151 | 0.648 | 0.648 | 0.007 | 0.029 |
| TK1 | 272.062 | 426.326 | 0.648 | 0.648 | 0.000 | 0.000 |
| SPOCK1 | 442.563 | 693.298 | 0.648 | 0.648 | 0.000 | 0.003 |
| TMEM238 | 11.744 | 18.393 | 0.647 | 0.647 | 0.000 | 0.003 |
| NCAPG | 200.007 | 312.826 | 0.645 | 0.645 | 0.000 | 0.000 |
| FAM64A | 96.358 | 150.641 | 0.645 | 0.645 | 0.000 | 0.000 |
| OCIAD2 | 4200.032 | 6563.759 | 0.644 | 0.644 | 0.000 | 0.000 |
| HES6 | 110.224 | 172.255 | 0.644 | 0.644 | 0.000 | 0.000 |
| EPN3 | 86.459 | 135.053 | 0.643 | 0.643 | 0.000 | 0.000 |
| SULF1 | 2688.680 | 4199.614 | 0.643 | 0.643 | 0.000 | 0.001 |
| SEZ6L2 | 182.985 | 285.445 | 0.641 | 0.641 | 0.000 | 0.000 |
| TUBAL3 | 37.546 | 58.515 | 0.640 | 0.640 | 0.001 | 0.008 |
| BCMO1 | 89.225 | 138.993 | 0.639 | 0.639 | 0.000 | 0.001 |
| AIM2 | 528.735 | 822.654 | 0.638 | 0.638 | 0.016 | 0.050 |
| ASPHD1 | 74.207 | 115.418 | 0.637 | 0.637 | 0.000 | 0.000 |
| FGB | 472.587 | 733.116 | 0.633 | 0.633 | 0.000 | 0.001 |
| DNAH2 | 83.400 | 129.338 | 0.633 | 0.633 | 0.000 | 0.001 |
| KIFC1 | 57.372 | 88.959 | 0.633 | 0.633 | 0.000 | 0.002 |
| LOC101929475 | 22.363 | 34.674 | 0.633 | 0.633 | 0.006 | 0.025 |
| EFNA2 | 28.783 | 44.578 | 0.631 | 0.631 | 0.011 | 0.039 |
| YBX2 | 27.255 | 42.188 | 0.630 | 0.630 | 0.004 | 0.018 |
| LOC100505920 | 49.519 | 76.606 | 0.629 | 0.629 | 0.000 | 0.001 |
| PHLDA2 | 852.501 | 1318.669 | 0.629 | 0.629 | 0.000 | 0.000 |
| CFB | 781.154 | 1206.112 | 0.627 | 0.627 | 0.000 | 0.000 |
| IL31RA | 35.279 | 54.461 | 0.626 | 0.626 | 0.013 | 0.043 |
| LOC400655 | 23.990 | 36.974 | 0.624 | 0.624 | 0.000 | 0.001 |
| CDK1 | 474.668 | 731.273 | 0.623 | 0.623 | 0.000 | 0.000 |
| HMGB3 | 2030.745 | 3127.987 | 0.623 | 0.623 | 0.000 | 0.000 |
| SLC6A3 | 124.146 | 191.216 | 0.623 | 0.623 | 0.002 | 0.009 |
| CCNB1 | 526.462 | 809.612 | 0.621 | 0.621 | 0.000 | 0.000 |
| GLRA3 | 16.333 | 25.091 | 0.619 | 0.619 | 0.001 | 0.008 |
| LOC100128993 | 2.400 | 3.684 | 0.619 | 0.619 | 0.001 | 0.006 |
| ZWINT | 1617.567 | 2481.156 | 0.617 | 0.617 | 0.000 | 0.000 |
| CCDC129 | 36.312 | 55.673 | 0.617 | 0.617 | 0.000 | 0.004 |
| CLDN4 | 379.536 | 581.813 | 0.616 | 0.616 | 0.000 | 0.000 |
| HMMR | 538.771 | 825.780 | 0.616 | 0.616 | 0.000 | 0.000 |
| CENPM | 178.663 | 273.805 | 0.616 | 0.616 | 0.000 | 0.000 |
| LOC101928710 | 32.836 | 50.317 | 0.616 | 0.616 | 0.001 | 0.006 |
| FHL2 | 1772.344 | 2714.760 | 0.615 | 0.615 | 0.000 | 0.000 |
| TRIM9 | 61.407 | 93.994 | 0.614 | 0.614 | 0.005 | 0.020 |
| AGR2 | 2602.978 | 3983.608 | 0.614 | 0.614 | 0.000 | 0.000 |
| MUC5B | 190.376 | 291.208 | 0.613 | 0.613 | 0.009 | 0.032 |
| POU3F2 | 32.317 | 49.404 | 0.612 | 0.612 | 0.001 | 0.006 |
| DNAJC22 | 112.386 | 171.795 | 0.612 | 0.612 | 0.000 | 0.000 |
| HOXA1 | 77.714 | 118.751 | 0.612 | 0.612 | 0.004 | 0.018 |
| LOC101928076 | 44.863 | 68.542 | 0.611 | 0.611 | 0.001 | 0.006 |
| WBSCR28 | 7.541 | 11.502 | 0.609 | 0.609 | 0.000 | 0.001 |
| ESPL1 | 237.590 | 362.384 | 0.609 | 0.609 | 0.000 | 0.000 |
| FAM83F | 70.712 | 107.774 | 0.608 | 0.608 | 0.000 | 0.000 |
| HSD3B7 | 201.800 | 307.339 | 0.607 | 0.607 | 0.000 | 0.000 |
| ECT2 | 492.292 | 749.678 | 0.607 | 0.607 | 0.000 | 0.000 |
| SULT4A1 | 39.060 | 59.471 | 0.607 | 0.607 | 0.014 | 0.045 |
| DLGAP5 | 325.447 | 495.466 | 0.606 | 0.606 | 0.000 | 0.000 |
| LINC00518 | 10.209 | 15.538 | 0.606 | 0.606 | 0.000 | 0.004 |
| FAM83D | 869.600 | 1323.259 | 0.606 | 0.606 | 0.000 | 0.001 |
| GSDMB | 292.343 | 444.669 | 0.605 | 0.605 | 0.012 | 0.041 |
| STX1A | 113.802 | 172.990 | 0.604 | 0.604 | 0.000 | 0.000 |
| LAD1 | 227.381 | 345.604 | 0.604 | 0.604 | 0.000 | 0.000 |
| HRASLS | 117.236 | 178.031 | 0.603 | 0.603 | 0.008 | 0.031 |
| LHFPL5 | 16.279 | 24.695 | 0.601 | 0.601 | 0.003 | 0.016 |
| SUGCT | 128.668 | 195.073 | 0.600 | 0.600 | 0.005 | 0.021 |
| MND1 | 75.544 | 114.513 | 0.600 | 0.600 | 0.000 | 0.000 |
| FLJ22184 | 17.067 | 25.858 | 0.599 | 0.599 | 0.000 | 0.002 |
| KRT16 | 31.503 | 47.723 | 0.599 | 0.599 | 0.014 | 0.047 |
| LGSN | 496.911 | 752.196 | 0.598 | 0.598 | 0.001 | 0.005 |
| SERPINB5 | 84.569 | 128.001 | 0.598 | 0.598 | 0.002 | 0.011 |
| CACNA1B | 13.372 | 20.233 | 0.598 | 0.598 | 0.004 | 0.019 |
| TMEM158 | 272.719 | 412.563 | 0.597 | 0.597 | 0.001 | 0.004 |
| WFDC10B | 30.251 | 45.759 | 0.597 | 0.597 | 0.002 | 0.012 |
| ARNTL2 | 167.726 | 253.605 | 0.596 | 0.596 | 0.001 | 0.006 |
| FAM169A | 166.366 | 251.529 | 0.596 | 0.596 | 0.000 | 0.000 |
| CCNB2 | 180.251 | 272.340 | 0.595 | 0.595 | 0.000 | 0.000 |
| UNC5CL | 450.625 | 680.778 | 0.595 | 0.595 | 0.000 | 0.000 |
| CPD | 1326.384 | 2003.349 | 0.595 | 0.595 | 0.000 | 0.000 |
| XKRX | 220.967 | 333.600 | 0.594 | 0.594 | 0.000 | 0.003 |
| RNF39 | 130.141 | 196.325 | 0.593 | 0.593 | 0.000 | 0.003 |
| LOC101929122 | 70.171 | 105.844 | 0.593 | 0.593 | 0.002 | 0.010 |
| UCHL1 | 152.268 | 229.633 | 0.593 | 0.593 | 0.008 | 0.029 |
| CBLC | 19.630 | 29.596 | 0.592 | 0.592 | 0.000 | 0.001 |
| MNX1 | 165.534 | 249.532 | 0.592 | 0.592 | 0.000 | 0.001 |
| ITGA11 | 99.888 | 150.454 | 0.591 | 0.591 | 0.001 | 0.005 |
| C10orf99 | 26.101 | 39.313 | 0.591 | 0.591 | 0.001 | 0.007 |
| TRIM59 | 458.352 | 690.329 | 0.591 | 0.591 | 0.000 | 0.000 |
| SOX9 | 472.925 | 712.127 | 0.591 | 0.591 | 0.000 | 0.000 |
| C1QTNF6 | 32.005 | 48.187 | 0.590 | 0.590 | 0.000 | 0.000 |
| HKDC1 | 114.339 | 172.148 | 0.590 | 0.590 | 0.000 | 0.002 |
| SLC15A1 | 40.295 | 60.660 | 0.590 | 0.590 | 0.007 | 0.028 |
| HOXB2 | 766.714 | 1153.384 | 0.589 | 0.589 | 0.010 | 0.036 |
| LRRC31 | 529.027 | 795.423 | 0.588 | 0.588 | 0.009 | 0.033 |
| CDCP1 | 284.127 | 427.131 | 0.588 | 0.588 | 0.000 | 0.000 |
| MTFR2 | 96.933 | 145.707 | 0.588 | 0.588 | 0.000 | 0.001 |
| ORC6 | 561.510 | 842.935 | 0.586 | 0.586 | 0.000 | 0.000 |
| RP11-353N14.2 | 33.621 | 50.417 | 0.585 | 0.585 | 0.000 | 0.002 |
| PLAU | 1871.578 | 2805.355 | 0.584 | 0.584 | 0.000 | 0.001 |
| PLEK2 | 587.458 | 880.234 | 0.583 | 0.583 | 0.000 | 0.000 |
| SLC41A2 | 429.670 | 643.687 | 0.583 | 0.583 | 0.000 | 0.000 |
| SPINT1 | 370.155 | 554.518 | 0.583 | 0.583 | 0.000 | 0.000 |
| KIF23 | 113.640 | 169.879 | 0.580 | 0.580 | 0.001 | 0.008 |
| MAD2L1 | 313.397 | 468.258 | 0.579 | 0.579 | 0.000 | 0.000 |
| STS | 422.629 | 631.441 | 0.579 | 0.579 | 0.000 | 0.001 |
| HOXB8 | 21.571 | 32.220 | 0.579 | 0.579 | 0.001 | 0.007 |
| HMGB3P1 | 157.983 | 235.883 | 0.578 | 0.578 | 0.000 | 0.000 |
| TUBB2A | 2047.169 | 3051.306 | 0.576 | 0.576 | 0.000 | 0.000 |
| TPBG | 1415.250 | 2109.184 | 0.576 | 0.576 | 0.000 | 0.000 |
| DCHS2 | 12.393 | 18.465 | 0.575 | 0.575 | 0.005 | 0.021 |
| KIF20A | 281.282 | 419.035 | 0.575 | 0.575 | 0.000 | 0.000 |
| BCAS1 | 198.354 | 295.467 | 0.575 | 0.575 | 0.001 | 0.004 |
| TIMP1 | 5490.609 | 8177.340 | 0.575 | 0.575 | 0.000 | 0.000 |
| PTGES | 127.941 | 190.511 | 0.574 | 0.574 | 0.000 | 0.000 |
| ERICH5 | 125.287 | 186.494 | 0.574 | 0.574 | 0.004 | 0.016 |
| ERO1L | 2211.348 | 3291.515 | 0.574 | 0.574 | 0.000 | 0.000 |
| ORC1 | 30.468 | 45.349 | 0.574 | 0.574 | 0.000 | 0.001 |
| HIST1H2AM | 47.227 | 70.267 | 0.573 | 0.573 | 0.001 | 0.004 |
| RP13-238F13.5 | 24.932 | 37.090 | 0.573 | 0.573 | 0.015 | 0.048 |
| KRTAP3-1 | 9.448 | 14.053 | 0.573 | 0.573 | 0.014 | 0.045 |
| REEP6 | 132.613 | 197.153 | 0.572 | 0.572 | 0.000 | 0.001 |
| GFAP | 7.604 | 11.284 | 0.569 | 0.569 | 0.000 | 0.001 |
| MFSD6L | 69.375 | 102.923 | 0.569 | 0.569 | 0.013 | 0.042 |
| FANCI | 227.127 | 336.777 | 0.568 | 0.568 | 0.000 | 0.000 |
| SLC27A2 | 424.874 | 629.931 | 0.568 | 0.568 | 0.000 | 0.001 |
| SGPP2 | 939.367 | 1392.391 | 0.568 | 0.568 | 0.000 | 0.000 |
| GPR56 | 948.666 | 1404.683 | 0.566 | 0.566 | 0.000 | 0.000 |
| LOC100505851 | 32.042 | 47.427 | 0.566 | 0.566 | 0.000 | 0.001 |
| QPRT | 548.889 | 811.093 | 0.563 | 0.563 | 0.002 | 0.010 |
| CENPN | 126.669 | 187.173 | 0.563 | 0.563 | 0.000 | 0.000 |
| TEX11 | 19.192 | 28.333 | 0.562 | 0.562 | 0.000 | 0.000 |
| OR7E14P | 178.562 | 263.466 | 0.561 | 0.561 | 0.000 | 0.000 |
| WDR86 | 49.679 | 73.265 | 0.560 | 0.560 | 0.002 | 0.012 |
| GLOD5 | 37.514 | 55.323 | 0.560 | 0.560 | 0.000 | 0.000 |
| POLQ | 123.422 | 182.007 | 0.560 | 0.560 | 0.000 | 0.003 |
| PRSS3P2 | 115.681 | 170.541 | 0.560 | 0.560 | 0.001 | 0.006 |
| CTSV | 239.364 | 352.841 | 0.560 | 0.560 | 0.000 | 0.000 |
| BAIAP2L1 | 934.455 | 1377.116 | 0.559 | 0.559 | 0.000 | 0.000 |
| WISP3 | 22.358 | 32.918 | 0.558 | 0.558 | 0.007 | 0.028 |
| FNDC4 | 67.441 | 99.165 | 0.556 | 0.556 | 0.000 | 0.002 |
| FCGBP | 1175.602 | 1728.336 | 0.556 | 0.556 | 0.003 | 0.014 |
| CDX2 | 6.285 | 9.235 | 0.555 | 0.555 | 0.009 | 0.034 |
| NUSAP1 | 573.631 | 842.760 | 0.555 | 0.555 | 0.000 | 0.000 |
| DEPDC1 | 89.841 | 131.962 | 0.555 | 0.555 | 0.000 | 0.000 |
| RNF212 | 44.510 | 65.362 | 0.554 | 0.554 | 0.003 | 0.013 |
| GINS2 | 104.915 | 153.900 | 0.553 | 0.553 | 0.000 | 0.000 |
| MMP9 | 2246.737 | 3293.544 | 0.552 | 0.552 | 0.002 | 0.009 |
| NCAPG2 | 283.235 | 415.092 | 0.551 | 0.551 | 0.000 | 0.000 |
| GMDS | 429.879 | 629.889 | 0.551 | 0.551 | 0.000 | 0.002 |
| CLDN1 | 1510.308 | 2212.739 | 0.551 | 0.551 | 0.000 | 0.000 |
| STK31 | 273.329 | 400.387 | 0.551 | 0.551 | 0.000 | 0.000 |
| LOC102724362 | 65.185 | 95.453 | 0.550 | 0.550 | 0.000 | 0.001 |
| IL23A | 47.747 | 69.908 | 0.550 | 0.550 | 0.001 | 0.007 |
| CDHR2 | 59.296 | 86.810 | 0.550 | 0.550 | 0.001 | 0.005 |
| PCAT6 | 172.734 | 252.685 | 0.549 | 0.549 | 0.000 | 0.000 |
| UGT2B28 | 117.351 | 171.628 | 0.548 | 0.548 | 0.015 | 0.048 |
| MARVELD3 | 172.861 | 252.787 | 0.548 | 0.548 | 0.000 | 0.000 |
| FAM83E | 17.711 | 25.896 | 0.548 | 0.548 | 0.001 | 0.004 |
| PLXNB3 | 15.990 | 23.365 | 0.547 | 0.547 | 0.000 | 0.002 |
| ADM2 | 36.423 | 53.214 | 0.547 | 0.547 | 0.002 | 0.010 |
| BC042029 | 11.483 | 16.759 | 0.545 | 0.545 | 0.011 | 0.037 |
| KIF2C | 216.409 | 315.810 | 0.545 | 0.545 | 0.000 | 0.000 |
| GBP6 | 41.797 | 60.969 | 0.545 | 0.545 | 0.001 | 0.007 |
| PKM | 449.593 | 655.376 | 0.544 | 0.544 | 0.000 | 0.000 |
| CTA-246H3.12 | 27.121 | 39.487 | 0.542 | 0.542 | 0.000 | 0.002 |
| SMKR1 | 141.145 | 205.498 | 0.542 | 0.542 | 0.000 | 0.002 |
| KIF18B | 245.245 | 356.600 | 0.540 | 0.540 | 0.000 | 0.000 |
| CENPI | 48.382 | 70.324 | 0.540 | 0.540 | 0.000 | 0.000 |
| ASPHD2 | 298.304 | 433.484 | 0.539 | 0.539 | 0.000 | 0.001 |
| KRT18 | 6653.612 | 9667.852 | 0.539 | 0.539 | 0.000 | 0.000 |
| APOBEC3B | 385.143 | 559.607 | 0.539 | 0.539 | 0.014 | 0.046 |
| AQP11 | 98.824 | 143.558 | 0.539 | 0.539 | 0.000 | 0.000 |
| EPS8L1 | 223.660 | 324.800 | 0.538 | 0.538 | 0.000 | 0.000 |
| HOXC11 | 41.015 | 59.535 | 0.538 | 0.538 | 0.009 | 0.032 |
| HMGA1 | 385.637 | 559.517 | 0.537 | 0.537 | 0.000 | 0.000 |
| FUT2 | 257.705 | 373.830 | 0.537 | 0.537 | 0.000 | 0.000 |
| DOK5 | 374.962 | 543.796 | 0.536 | 0.536 | 0.002 | 0.010 |
| CCDC24 | 79.720 | 115.599 | 0.536 | 0.536 | 0.000 | 0.000 |
| FAM90A1 | 40.568 | 58.799 | 0.535 | 0.535 | 0.003 | 0.013 |
| TMEM184A | 170.661 | 247.186 | 0.534 | 0.534 | 0.000 | 0.000 |
| RDM1 | 31.219 | 45.208 | 0.534 | 0.534 | 0.000 | 0.001 |
| HN1 | 1166.047 | 1688.535 | 0.534 | 0.534 | 0.000 | 0.000 |
| CILP | 1341.080 | 1941.879 | 0.534 | 0.534 | 0.009 | 0.033 |
| SLC39A11 | 737.095 | 1066.645 | 0.533 | 0.533 | 0.000 | 0.000 |
| STIL | 596.279 | 862.471 | 0.532 | 0.532 | 0.000 | 0.000 |
| NME1 | 1730.525 | 2501.780 | 0.532 | 0.532 | 0.000 | 0.000 |
| SERINC2 | 120.105 | 173.597 | 0.531 | 0.531 | 0.000 | 0.000 |
| VSIG1 | 519.397 | 750.116 | 0.530 | 0.530 | 0.000 | 0.001 |
| AURKA | 439.630 | 634.391 | 0.529 | 0.529 | 0.000 | 0.000 |
| CLDN12 | 2316.911 | 3342.760 | 0.529 | 0.529 | 0.000 | 0.000 |
| PHKA1 | 265.142 | 382.456 | 0.529 | 0.529 | 0.000 | 0.000 |
| CRLF2 | 32.493 | 46.870 | 0.529 | 0.529 | 0.005 | 0.022 |
| HOXA11 | 14.723 | 21.229 | 0.528 | 0.528 | 0.015 | 0.047 |
| CENPE | 129.474 | 186.649 | 0.528 | 0.528 | 0.000 | 0.000 |
| MDK | 403.811 | 582.011 | 0.527 | 0.527 | 0.000 | 0.000 |
| RP11-44F21.5 | 226.811 | 326.866 | 0.527 | 0.527 | 0.000 | 0.001 |
| TENM4 | 217.515 | 313.240 | 0.526 | 0.526 | 0.000 | 0.000 |
| LOC100506098 | 616.758 | 888.151 | 0.526 | 0.526 | 0.000 | 0.001 |
| DSG2 | 1923.860 | 2768.253 | 0.525 | 0.525 | 0.000 | 0.000 |
| TNFSF4 | 342.991 | 493.466 | 0.525 | 0.525 | 0.000 | 0.003 |
| BMP8A | 32.880 | 47.230 | 0.522 | 0.522 | 0.002 | 0.011 |
| TRPM4 | 164.752 | 236.618 | 0.522 | 0.522 | 0.000 | 0.003 |
| APLP1 | 33.092 | 47.503 | 0.522 | 0.522 | 0.011 | 0.039 |
| CKAP2L | 82.489 | 118.366 | 0.521 | 0.521 | 0.000 | 0.000 |
| TTC9 | 222.094 | 318.564 | 0.520 | 0.520 | 0.000 | 0.000 |
| CDC6 | 73.861 | 105.933 | 0.520 | 0.520 | 0.000 | 0.000 |
| MACC1 | 531.993 | 762.740 | 0.520 | 0.520 | 0.000 | 0.000 |
| COL8A2 | 436.390 | 625.627 | 0.520 | 0.520 | 0.010 | 0.035 |
| RNF183 | 205.201 | 294.122 | 0.519 | 0.519 | 0.005 | 0.020 |
| OVOL1 | 106.995 | 153.352 | 0.519 | 0.519 | 0.000 | 0.000 |
| SYNGR3 | 132.811 | 190.305 | 0.519 | 0.519 | 0.002 | 0.011 |
| C5orf46 | 26.537 | 38.006 | 0.518 | 0.518 | 0.005 | 0.022 |
| ZNF663P | 7.394 | 10.584 | 0.517 | 0.517 | 0.004 | 0.018 |
| XDH | 504.365 | 721.933 | 0.517 | 0.517 | 0.000 | 0.001 |
| RP11-524D16__A.3 | 60.299 | 86.246 | 0.516 | 0.516 | 0.000 | 0.000 |
| MKI67 | 173.342 | 247.915 | 0.516 | 0.516 | 0.000 | 0.000 |
| PDLIM4 | 162.486 | 232.377 | 0.516 | 0.516 | 0.000 | 0.000 |
| LINGO1 | 59.288 | 84.755 | 0.516 | 0.516 | 0.007 | 0.027 |
| CST2 | 73.871 | 105.529 | 0.515 | 0.515 | 0.000 | 0.000 |
| COL22A1 | 76.179 | 108.717 | 0.513 | 0.513 | 0.004 | 0.018 |
| CARD14 | 31.301 | 44.669 | 0.513 | 0.513 | 0.002 | 0.011 |
| BRIP1 | 111.145 | 158.453 | 0.512 | 0.512 | 0.000 | 0.000 |
| SRD5A1 | 973.617 | 1387.652 | 0.511 | 0.511 | 0.000 | 0.000 |
| DBNDD1 | 97.245 | 138.574 | 0.511 | 0.511 | 0.000 | 0.000 |
| CRYBA2 | 5.179 | 7.380 | 0.511 | 0.511 | 0.010 | 0.035 |
| LOC100287525 | 123.246 | 175.575 | 0.511 | 0.511 | 0.000 | 0.000 |
| EEF1A2 | 83.043 | 118.263 | 0.510 | 0.510 | 0.000 | 0.001 |
| SLC9A3R1 | 891.127 | 1269.073 | 0.510 | 0.510 | 0.005 | 0.021 |
| PSMD3 | 326.230 | 464.377 | 0.509 | 0.509 | 0.000 | 0.001 |
| S100A11 | 1827.534 | 2600.971 | 0.509 | 0.509 | 0.000 | 0.000 |
| ZNF280B | 143.086 | 203.579 | 0.509 | 0.509 | 0.000 | 0.001 |
| SLC6A17 | 37.167 | 52.853 | 0.508 | 0.508 | 0.002 | 0.010 |
| ARSE | 324.473 | 460.888 | 0.506 | 0.506 | 0.001 | 0.007 |
| PGM2L1 | 655.080 | 930.487 | 0.506 | 0.506 | 0.000 | 0.000 |
| THBS2 | 4414.258 | 6266.587 | 0.506 | 0.506 | 0.000 | 0.001 |
| C1orf106 | 1728.636 | 2453.582 | 0.505 | 0.505 | 0.000 | 0.002 |
| UBXN10-AS1 | 216.425 | 307.032 | 0.505 | 0.505 | 0.000 | 0.000 |
| CHEK1 | 146.578 | 207.890 | 0.504 | 0.504 | 0.000 | 0.000 |
| IL32 | 168.564 | 239.049 | 0.504 | 0.504 | 0.015 | 0.049 |
| SFN | 6742.750 | 9559.349 | 0.504 | 0.504 | 0.000 | 0.000 |
| LINC00925 | 12.959 | 18.364 | 0.503 | 0.503 | 0.012 | 0.042 |
| AGMAT | 77.237 | 109.445 | 0.503 | 0.503 | 0.000 | 0.001 |
| CDC25C | 24.671 | 34.958 | 0.503 | 0.503 | 0.000 | 0.001 |
| SGOL2 | 252.207 | 357.120 | 0.502 | 0.502 | 0.000 | 0.000 |
| ARL9 | 57.036 | 80.673 | 0.500 | 0.500 | 0.000 | 0.000 |
| PCDHGC5 | 3.513 | 4.968 | 0.500 | 0.500 | 0.011 | 0.038 |
| LMOD1 | 432.421 | 305.734 | -0.500 | 0.500 | 0.009 | 0.033 |
| SEMA6D | 190.809 | 134.887 | -0.500 | 0.500 | 0.002 | 0.010 |
| FRY | 886.847 | 626.649 | -0.501 | 0.501 | 0.000 | 0.000 |
| COLGALT2 | 137.591 | 97.219 | -0.501 | 0.501 | 0.003 | 0.015 |
| PDE8B | 175.034 | 123.673 | -0.501 | 0.501 | 0.000 | 0.002 |
| TBX5-AS1 | 360.861 | 254.778 | -0.502 | 0.502 | 0.000 | 0.000 |
| SCAI | 553.152 | 390.541 | -0.502 | 0.502 | 0.000 | 0.000 |
| STARD8 | 367.522 | 259.473 | -0.502 | 0.502 | 0.004 | 0.019 |
| ABCA6 | 326.316 | 230.358 | -0.502 | 0.502 | 0.000 | 0.000 |
| DENND3 | 418.755 | 295.584 | -0.503 | 0.503 | 0.000 | 0.000 |
| GPR85 | 140.083 | 98.865 | -0.503 | 0.503 | 0.009 | 0.032 |
| MYH2 | 53.616 | 37.794 | -0.504 | 0.504 | 0.000 | 0.002 |
| PPAP2B | 2690.369 | 1896.360 | -0.505 | 0.505 | 0.000 | 0.001 |
| TBX5 | 414.440 | 292.087 | -0.505 | 0.505 | 0.000 | 0.000 |
| PHACTR1 | 386.703 | 272.481 | -0.505 | 0.505 | 0.000 | 0.000 |
| ZFP36 | 5916.378 | 4168.399 | -0.505 | 0.505 | 0.001 | 0.007 |
| NEXN | 1355.425 | 953.981 | -0.507 | 0.507 | 0.000 | 0.003 |
| OR52K3P | 62.546 | 44.018 | -0.507 | 0.507 | 0.000 | 0.001 |
| SBSPON | 156.452 | 110.088 | -0.507 | 0.507 | 0.001 | 0.006 |
| SMCO3 | 222.721 | 156.695 | -0.507 | 0.507 | 0.000 | 0.001 |
| CCDC69 | 324.661 | 228.251 | -0.508 | 0.508 | 0.000 | 0.000 |
| WWC2 | 55.779 | 39.212 | -0.508 | 0.508 | 0.001 | 0.007 |
| SYDE2 | 162.097 | 113.926 | -0.509 | 0.509 | 0.002 | 0.012 |
| ESAM | 467.314 | 328.369 | -0.509 | 0.509 | 0.002 | 0.010 |
| PECAM1 | 2700.034 | 1896.304 | -0.510 | 0.510 | 0.000 | 0.001 |
| SLCO4C1 | 562.973 | 395.372 | -0.510 | 0.510 | 0.003 | 0.013 |
| RUNX1T1 | 446.458 | 313.496 | -0.510 | 0.510 | 0.000 | 0.001 |
| AK021977 | 666.793 | 467.719 | -0.512 | 0.512 | 0.001 | 0.004 |
| NPR1 | 260.846 | 182.832 | -0.513 | 0.513 | 0.000 | 0.001 |
| TRHDE-AS1 | 37.690 | 26.417 | -0.513 | 0.513 | 0.005 | 0.020 |
| CD160 | 102.895 | 72.000 | -0.515 | 0.515 | 0.014 | 0.045 |
| ID4 | 1185.466 | 828.223 | -0.517 | 0.517 | 0.001 | 0.005 |
| RYR2 | 146.165 | 101.934 | -0.520 | 0.520 | 0.000 | 0.001 |
| SELP | 823.675 | 574.095 | -0.521 | 0.521 | 0.000 | 0.003 |
| PAQR5 | 352.559 | 245.675 | -0.521 | 0.521 | 0.003 | 0.015 |
| ARHGAP28 | 183.447 | 127.744 | -0.522 | 0.522 | 0.000 | 0.001 |
| SPIDR | 441.239 | 307.142 | -0.523 | 0.523 | 0.000 | 0.000 |
| CTSG | 224.658 | 156.376 | -0.523 | 0.523 | 0.004 | 0.017 |
| LAMC3 | 138.648 | 96.495 | -0.523 | 0.523 | 0.002 | 0.010 |
| LOC100506699 | 73.237 | 50.925 | -0.524 | 0.524 | 0.002 | 0.009 |
| USP44 | 143.105 | 99.501 | -0.524 | 0.524 | 0.002 | 0.010 |
| RP11-155O18.6 | 39.898 | 27.725 | -0.525 | 0.525 | 0.000 | 0.000 |
| MEOX2 | 452.180 | 314.122 | -0.526 | 0.526 | 0.001 | 0.005 |
| KIAA1462 | 512.159 | 355.705 | -0.526 | 0.526 | 0.002 | 0.011 |
| PCDH12 | 410.499 | 284.531 | -0.529 | 0.529 | 0.011 | 0.037 |
| LHFP | 1663.379 | 1152.777 | -0.529 | 0.529 | 0.000 | 0.000 |
| CD69 | 3657.026 | 2533.206 | -0.530 | 0.530 | 0.000 | 0.001 |
| CACNA2D2 | 2180.810 | 1510.418 | -0.530 | 0.530 | 0.000 | 0.003 |
| PPP1R14A | 638.940 | 442.153 | -0.531 | 0.531 | 0.001 | 0.008 |
| LIFR | 1639.837 | 1134.125 | -0.532 | 0.532 | 0.000 | 0.001 |
| ITM2A | 1318.305 | 911.642 | -0.532 | 0.532 | 0.000 | 0.002 |
| TUBB1 | 49.608 | 34.266 | -0.534 | 0.534 | 0.000 | 0.000 |
| TSPAN18 | 236.133 | 163.001 | -0.535 | 0.535 | 0.000 | 0.002 |
| GIMAP7 | 1658.973 | 1144.747 | -0.535 | 0.535 | 0.000 | 0.001 |
| PEG3-AS1 | 185.447 | 127.830 | -0.537 | 0.537 | 0.000 | 0.001 |
| EFEMP1 | 4533.725 | 3123.787 | -0.537 | 0.537 | 0.000 | 0.000 |
| CYP2B7P | 2474.693 | 1704.130 | -0.538 | 0.538 | 0.015 | 0.047 |
| ITGA1 | 1014.036 | 697.536 | -0.540 | 0.540 | 0.000 | 0.000 |
| KCNJ8 | 942.965 | 648.468 | -0.540 | 0.540 | 0.004 | 0.019 |
| GATA2 | 432.634 | 297.361 | -0.541 | 0.541 | 0.000 | 0.001 |
| LOC286189 | 612.467 | 420.823 | -0.541 | 0.541 | 0.000 | 0.003 |
| ATP1A2 | 250.151 | 171.856 | -0.542 | 0.542 | 0.001 | 0.007 |
| AF070581 | 367.986 | 252.762 | -0.542 | 0.542 | 0.013 | 0.044 |
| PABPC5 | 67.339 | 46.216 | -0.543 | 0.543 | 0.001 | 0.004 |
| ERBB4 | 174.030 | 119.295 | -0.545 | 0.545 | 0.001 | 0.007 |
| ABCA9 | 85.857 | 58.833 | -0.545 | 0.545 | 0.000 | 0.001 |
| CACHD1 | 1409.971 | 965.855 | -0.546 | 0.546 | 0.003 | 0.014 |
| CASS4 | 75.731 | 51.860 | -0.546 | 0.546 | 0.000 | 0.000 |
| RBM24 | 245.574 | 168.098 | -0.547 | 0.547 | 0.005 | 0.021 |
| PTGDS | 2694.040 | 1844.086 | -0.547 | 0.547 | 0.000 | 0.003 |
| LTBP4 | 296.367 | 202.858 | -0.547 | 0.547 | 0.000 | 0.003 |
| REEP1 | 379.367 | 259.620 | -0.547 | 0.547 | 0.001 | 0.004 |
| C1QTNF2 | 146.082 | 99.786 | -0.550 | 0.550 | 0.001 | 0.005 |
| TGM1 | 24.833 | 16.950 | -0.551 | 0.551 | 0.003 | 0.015 |
| ST6GALNAC3 | 167.732 | 114.466 | -0.551 | 0.551 | 0.000 | 0.000 |
| BAI3 | 130.717 | 89.194 | -0.551 | 0.551 | 0.002 | 0.009 |
| CAV2 | 3277.147 | 2233.705 | -0.553 | 0.553 | 0.000 | 0.001 |
| AK094644 | 35.548 | 24.210 | -0.554 | 0.554 | 0.011 | 0.037 |
| SH2D3C | 260.829 | 177.488 | -0.555 | 0.555 | 0.002 | 0.012 |
| LIN7A | 136.373 | 92.774 | -0.556 | 0.556 | 0.001 | 0.008 |
| LDB3 | 89.900 | 61.108 | -0.557 | 0.557 | 0.004 | 0.017 |
| PALMD | 365.444 | 248.376 | -0.557 | 0.557 | 0.001 | 0.004 |
| FXYD6 | 1471.018 | 999.669 | -0.557 | 0.557 | 0.000 | 0.001 |
| FZD4 | 452.828 | 307.687 | -0.557 | 0.557 | 0.003 | 0.014 |
| HOXA5 | 1031.227 | 700.525 | -0.558 | 0.558 | 0.009 | 0.033 |
| KLF15 | 275.953 | 187.279 | -0.559 | 0.559 | 0.000 | 0.000 |
| SRL | 41.201 | 27.956 | -0.560 | 0.560 | 0.015 | 0.048 |
| CYP4B1 | 4595.668 | 3112.724 | -0.562 | 0.562 | 0.000 | 0.001 |
| ADAMTS9 | 250.362 | 169.560 | -0.562 | 0.562 | 0.013 | 0.042 |
| NPNT | 1897.586 | 1284.928 | -0.562 | 0.562 | 0.000 | 0.000 |
| TNFRSF10D | 330.055 | 223.400 | -0.563 | 0.563 | 0.001 | 0.005 |
| LOC153577 | 97.991 | 66.323 | -0.563 | 0.563 | 0.000 | 0.000 |
| LOC101928612 | 95.619 | 64.708 | -0.563 | 0.563 | 0.000 | 0.001 |
| ECM2 | 1481.310 | 1001.745 | -0.564 | 0.564 | 0.000 | 0.000 |
| PRX | 92.362 | 62.416 | -0.565 | 0.565 | 0.002 | 0.012 |
| FLI1 | 642.141 | 433.733 | -0.566 | 0.566 | 0.000 | 0.001 |
| FUT1 | 332.667 | 224.657 | -0.566 | 0.566 | 0.000 | 0.003 |
| CAV1 | 9720.868 | 6561.561 | -0.567 | 0.567 | 0.000 | 0.000 |
| SSTR1 | 141.276 | 95.329 | -0.568 | 0.568 | 0.016 | 0.050 |
| CCL23 | 93.630 | 63.154 | -0.568 | 0.568 | 0.014 | 0.047 |
| FAM189A2 | 881.356 | 594.423 | -0.568 | 0.568 | 0.002 | 0.011 |
| ITGA8 | 792.859 | 533.042 | -0.573 | 0.573 | 0.000 | 0.004 |
| RP11-295M18.6 | 155.391 | 104.378 | -0.574 | 0.574 | 0.000 | 0.001 |
| EBF1 | 589.090 | 394.959 | -0.577 | 0.577 | 0.004 | 0.018 |
| CXCL3 | 527.860 | 353.862 | -0.577 | 0.577 | 0.002 | 0.010 |
| MASP1 | 61.854 | 41.451 | -0.577 | 0.577 | 0.005 | 0.022 |
| CFP | 74.378 | 49.831 | -0.578 | 0.578 | 0.003 | 0.015 |
| SCN1A | 188.347 | 126.078 | -0.579 | 0.579 | 0.005 | 0.020 |
| HMGCS2 | 102.624 | 68.695 | -0.579 | 0.579 | 0.005 | 0.020 |
| SFTPC | 11208.785 | 7495.510 | -0.581 | 0.581 | 0.001 | 0.004 |
| KLF9 | 673.403 | 450.243 | -0.581 | 0.581 | 0.000 | 0.002 |
| PRTG | 211.765 | 141.538 | -0.581 | 0.581 | 0.000 | 0.001 |
| PGR | 304.320 | 203.379 | -0.581 | 0.581 | 0.001 | 0.008 |
| ABLIM3 | 186.225 | 124.383 | -0.582 | 0.582 | 0.000 | 0.001 |
| GPC3 | 251.793 | 168.109 | -0.583 | 0.583 | 0.001 | 0.006 |
| CAV3 | 98.638 | 65.840 | -0.583 | 0.583 | 0.001 | 0.006 |
| TRIM71 | 179.379 | 119.731 | -0.583 | 0.583 | 0.001 | 0.006 |
| CXorf36 | 80.224 | 53.519 | -0.584 | 0.584 | 0.000 | 0.002 |
| HIGD1B | 317.153 | 211.547 | -0.584 | 0.584 | 0.003 | 0.014 |
| SMAD9 | 588.720 | 392.661 | -0.584 | 0.584 | 0.000 | 0.000 |
| TBX3 | 242.873 | 161.946 | -0.585 | 0.585 | 0.001 | 0.007 |
| ADAMTS9-AS2 | 123.274 | 82.164 | -0.585 | 0.585 | 0.000 | 0.001 |
| AFAP1L1 | 272.488 | 181.532 | -0.586 | 0.586 | 0.000 | 0.001 |
| CASP12 | 64.290 | 42.828 | -0.586 | 0.586 | 0.000 | 0.000 |
| ERG | 390.149 | 259.849 | -0.586 | 0.586 | 0.000 | 0.001 |
| ARHGEF26 | 544.458 | 362.109 | -0.588 | 0.588 | 0.003 | 0.015 |
| C3orf70 | 371.243 | 246.885 | -0.589 | 0.589 | 0.000 | 0.000 |
| HSPB2 | 150.386 | 99.989 | -0.589 | 0.589 | 0.000 | 0.004 |
| CHI3L2 | 456.231 | 303.315 | -0.589 | 0.589 | 0.002 | 0.010 |
| AOX1 | 384.413 | 255.518 | -0.589 | 0.589 | 0.003 | 0.013 |
| CLIC5 | 1316.448 | 874.839 | -0.590 | 0.590 | 0.007 | 0.028 |
| COL4A6 | 113.068 | 75.120 | -0.590 | 0.590 | 0.007 | 0.027 |
| SHANK3 | 324.386 | 215.375 | -0.591 | 0.591 | 0.003 | 0.015 |
| GIMAP6 | 1822.145 | 1209.778 | -0.591 | 0.591 | 0.000 | 0.001 |
| WASF3 | 971.586 | 644.636 | -0.592 | 0.592 | 0.000 | 0.000 |
| OLFML1 | 1068.415 | 708.735 | -0.592 | 0.592 | 0.000 | 0.003 |
| TTLL7 | 244.436 | 162.088 | -0.593 | 0.593 | 0.001 | 0.007 |
| RAMP2 | 98.691 | 65.363 | -0.594 | 0.594 | 0.000 | 0.000 |
| HBD | 70.805 | 46.877 | -0.595 | 0.595 | 0.003 | 0.016 |
| TIE1 | 122.730 | 81.228 | -0.595 | 0.595 | 0.000 | 0.002 |
| LOC100128751 | 36.259 | 23.993 | -0.596 | 0.596 | 0.001 | 0.005 |
| PCAT19 | 529.815 | 350.480 | -0.596 | 0.596 | 0.000 | 0.003 |
| TNFAIP8L3 | 51.652 | 34.128 | -0.598 | 0.598 | 0.002 | 0.009 |
| BC043227 | 13.458 | 8.884 | -0.599 | 0.599 | 0.002 | 0.012 |
| LOC101927943 | 143.407 | 94.626 | -0.600 | 0.600 | 0.000 | 0.001 |
| RPL13AP17 | 401.431 | 264.769 | -0.600 | 0.600 | 0.000 | 0.001 |
| PAPPA | 247.343 | 163.069 | -0.601 | 0.601 | 0.002 | 0.012 |
| PDE2A | 218.820 | 144.257 | -0.601 | 0.601 | 0.000 | 0.001 |
| CRTAC1 | 319.519 | 210.632 | -0.601 | 0.601 | 0.002 | 0.011 |
| PRIMA1 | 130.231 | 85.809 | -0.602 | 0.602 | 0.002 | 0.011 |
| INSC | 60.232 | 39.624 | -0.604 | 0.604 | 0.000 | 0.004 |
| NRN1 | 470.910 | 309.439 | -0.606 | 0.606 | 0.001 | 0.005 |
| ADARB1 | 442.258 | 290.443 | -0.607 | 0.607 | 0.000 | 0.000 |
| FHL1 | 3225.916 | 2118.116 | -0.607 | 0.607 | 0.000 | 0.000 |
| PLEKHH2 | 1129.541 | 741.541 | -0.607 | 0.607 | 0.000 | 0.000 |
| SOCS2 | 1154.812 | 758.003 | -0.607 | 0.607 | 0.000 | 0.002 |
| NFASC | 133.243 | 87.234 | -0.611 | 0.611 | 0.000 | 0.001 |
| RGCC | 3732.234 | 2443.047 | -0.611 | 0.611 | 0.000 | 0.000 |
| DPT | 779.507 | 510.248 | -0.611 | 0.611 | 0.000 | 0.002 |
| PEG3 | 597.366 | 390.765 | -0.612 | 0.612 | 0.000 | 0.001 |
| DUOX1 | 244.065 | 159.640 | -0.612 | 0.612 | 0.001 | 0.004 |
| GHR | 587.353 | 383.984 | -0.613 | 0.613 | 0.000 | 0.002 |
| DNASE2B | 95.529 | 62.413 | -0.614 | 0.614 | 0.010 | 0.037 |
| FMO2 | 4129.994 | 2692.182 | -0.617 | 0.617 | 0.003 | 0.014 |
| C7 | 4366.765 | 2845.934 | -0.618 | 0.618 | 0.000 | 0.001 |
| FXYD1 | 178.263 | 116.066 | -0.619 | 0.619 | 0.000 | 0.003 |
| PLAC9 | 138.530 | 90.172 | -0.619 | 0.619 | 0.000 | 0.003 |
| VGLL3 | 1571.659 | 1021.796 | -0.621 | 0.621 | 0.000 | 0.001 |
| WISP2 | 239.138 | 155.184 | -0.624 | 0.624 | 0.011 | 0.039 |
| PEAR1 | 371.149 | 240.447 | -0.626 | 0.626 | 0.000 | 0.000 |
| RP11-401P9.4 | 597.953 | 387.006 | -0.628 | 0.628 | 0.000 | 0.001 |
| GIMAP8 | 1028.567 | 665.469 | -0.628 | 0.628 | 0.000 | 0.004 |
| SASH1 | 2570.195 | 1661.063 | -0.630 | 0.630 | 0.000 | 0.000 |
| PRG4 | 244.657 | 158.028 | -0.631 | 0.631 | 0.011 | 0.037 |
| MICU3 | 928.839 | 599.932 | -0.631 | 0.631 | 0.000 | 0.000 |
| ATOH8 | 280.975 | 181.463 | -0.631 | 0.631 | 0.000 | 0.001 |
| KANK4 | 559.132 | 361.031 | -0.631 | 0.631 | 0.001 | 0.004 |
| LOC100507311 | 391.278 | 252.617 | -0.631 | 0.631 | 0.000 | 0.003 |
| CDH13 | 322.171 | 207.619 | -0.634 | 0.634 | 0.006 | 0.026 |
| LOC285812 | 817.741 | 525.521 | -0.638 | 0.638 | 0.000 | 0.000 |
| HSPA12B | 252.020 | 161.940 | -0.638 | 0.638 | 0.004 | 0.017 |
| PCDH9 | 200.706 | 128.966 | -0.638 | 0.638 | 0.002 | 0.010 |
| VIP | 66.359 | 42.599 | -0.639 | 0.639 | 0.010 | 0.035 |
| ACTN2 | 46.670 | 29.920 | -0.641 | 0.641 | 0.000 | 0.001 |
| ADAMTS15 | 135.324 | 86.710 | -0.642 | 0.642 | 0.001 | 0.008 |
| FBLN5 | 1141.276 | 731.272 | -0.642 | 0.642 | 0.000 | 0.000 |
| RP11-305O6.3 | 384.365 | 246.271 | -0.642 | 0.642 | 0.001 | 0.004 |
| WIF1 | 5226.058 | 3348.322 | -0.642 | 0.642 | 0.003 | 0.013 |
| ZNF331 | 1279.226 | 819.179 | -0.643 | 0.643 | 0.000 | 0.001 |
| PKNOX2 | 219.151 | 140.311 | -0.643 | 0.643 | 0.000 | 0.000 |
| NTNG1 | 117.712 | 75.362 | -0.643 | 0.643 | 0.001 | 0.008 |
| ACKR1 | 1127.329 | 721.685 | -0.643 | 0.643 | 0.003 | 0.013 |
| FEZ1 | 649.639 | 415.868 | -0.644 | 0.644 | 0.000 | 0.002 |
| SFTA1P | 733.250 | 469.341 | -0.644 | 0.644 | 0.001 | 0.007 |
| CACNB4 | 90.290 | 57.707 | -0.646 | 0.646 | 0.004 | 0.019 |
| SFRP1 | 120.019 | 76.654 | -0.647 | 0.647 | 0.011 | 0.039 |
| CLEC1A | 343.948 | 219.545 | -0.648 | 0.648 | 0.000 | 0.001 |
| RP11-674P19.2 | 44.698 | 28.527 | -0.648 | 0.648 | 0.000 | 0.001 |
| TMTC1 | 354.587 | 226.198 | -0.649 | 0.649 | 0.004 | 0.017 |
| SHC3 | 311.682 | 198.689 | -0.650 | 0.650 | 0.000 | 0.000 |
| KLF4 | 4672.739 | 2972.882 | -0.652 | 0.652 | 0.001 | 0.004 |
| RYR3 | 99.377 | 63.198 | -0.653 | 0.653 | 0.000 | 0.002 |
| RHOJ | 392.841 | 249.817 | -0.653 | 0.653 | 0.000 | 0.001 |
| DENND2A | 235.835 | 149.838 | -0.654 | 0.654 | 0.000 | 0.000 |
| HDC | 375.656 | 238.657 | -0.654 | 0.654 | 0.001 | 0.004 |
| RP11-38P22.2 | 916.835 | 582.042 | -0.656 | 0.656 | 0.010 | 0.036 |
| CSRNP1 | 1348.162 | 855.724 | -0.656 | 0.656 | 0.000 | 0.004 |
| PDK4 | 2054.620 | 1303.062 | -0.657 | 0.657 | 0.004 | 0.019 |
| GFRA1 | 249.262 | 158.074 | -0.657 | 0.657 | 0.000 | 0.002 |
| AMPD1 | 229.618 | 145.614 | -0.657 | 0.657 | 0.001 | 0.006 |
| CYYR1 | 1556.562 | 986.921 | -0.657 | 0.657 | 0.000 | 0.001 |
| MAL | 1152.478 | 730.375 | -0.658 | 0.658 | 0.000 | 0.000 |
| MYOC | 162.835 | 103.174 | -0.658 | 0.658 | 0.000 | 0.001 |
| RAPGEF4 | 270.734 | 171.453 | -0.659 | 0.659 | 0.000 | 0.001 |
| CCM2L | 334.356 | 211.471 | -0.661 | 0.661 | 0.001 | 0.006 |
| CPB2 | 2099.327 | 1326.879 | -0.662 | 0.662 | 0.005 | 0.021 |
| SOX17 | 284.688 | 179.886 | -0.662 | 0.662 | 0.001 | 0.008 |
| CCDC102B | 911.897 | 576.175 | -0.662 | 0.662 | 0.000 | 0.000 |
| GPR133 | 1196.979 | 755.267 | -0.664 | 0.664 | 0.000 | 0.001 |
| VWF | 2189.605 | 1381.242 | -0.665 | 0.665 | 0.001 | 0.006 |
| COL4A3 | 480.503 | 302.976 | -0.665 | 0.665 | 0.000 | 0.003 |
| SYNPO2L | 31.737 | 20.010 | -0.665 | 0.665 | 0.001 | 0.008 |
| SCN7A | 1655.169 | 1043.502 | -0.666 | 0.666 | 0.000 | 0.000 |
| ADAMTS8 | 850.106 | 535.466 | -0.667 | 0.667 | 0.003 | 0.013 |
| MYZAP | 683.167 | 430.272 | -0.667 | 0.667 | 0.006 | 0.023 |
| IL18R1 | 425.972 | 268.025 | -0.668 | 0.668 | 0.000 | 0.002 |
| MS4A2 | 195.996 | 123.197 | -0.670 | 0.670 | 0.000 | 0.000 |
| GPM6B | 1126.985 | 708.342 | -0.670 | 0.670 | 0.000 | 0.001 |
| LOC101928161 | 13.435 | 8.437 | -0.671 | 0.671 | 0.007 | 0.027 |
| TMEM88 | 139.845 | 87.565 | -0.675 | 0.675 | 0.000 | 0.001 |
| EP300-AS1 | 1099.665 | 688.429 | -0.676 | 0.676 | 0.003 | 0.015 |
| PDE5A | 602.072 | 376.305 | -0.678 | 0.678 | 0.000 | 0.001 |
| BCHE | 1315.384 | 820.390 | -0.681 | 0.681 | 0.001 | 0.004 |
| AQP4 | 1989.226 | 1240.489 | -0.681 | 0.681 | 0.000 | 0.001 |
| CCDC141 | 72.586 | 45.260 | -0.681 | 0.681 | 0.002 | 0.009 |
| NOSTRIN | 1030.349 | 641.240 | -0.684 | 0.684 | 0.000 | 0.003 |
| SYNPO2 | 1234.945 | 768.482 | -0.684 | 0.684 | 0.000 | 0.003 |
| TRIM63 | 64.185 | 39.897 | -0.686 | 0.686 | 0.001 | 0.007 |
| SCN3B | 55.102 | 34.246 | -0.686 | 0.686 | 0.010 | 0.036 |
| SMAD6 | 357.685 | 222.304 | -0.686 | 0.686 | 0.002 | 0.009 |
| MMRN2 | 513.606 | 319.064 | -0.687 | 0.687 | 0.000 | 0.002 |
| MAP3K8 | 1103.995 | 685.706 | -0.687 | 0.687 | 0.000 | 0.000 |
| GNG11 | 1300.093 | 806.118 | -0.690 | 0.690 | 0.000 | 0.002 |
| PGM5 | 292.257 | 181.187 | -0.690 | 0.690 | 0.001 | 0.004 |
| ZNF385B | 1390.278 | 861.706 | -0.690 | 0.690 | 0.002 | 0.010 |
| PREX2 | 434.831 | 269.269 | -0.691 | 0.691 | 0.000 | 0.000 |
| SLIT2 | 3338.391 | 2066.181 | -0.692 | 0.692 | 0.000 | 0.002 |
| RUNDC3B | 281.401 | 174.003 | -0.694 | 0.694 | 0.008 | 0.029 |
| GRK5 | 733.296 | 452.651 | -0.696 | 0.696 | 0.000 | 0.002 |
| ABI3BP | 1654.527 | 1020.401 | -0.697 | 0.697 | 0.000 | 0.000 |
| AGTR1 | 548.197 | 337.849 | -0.698 | 0.698 | 0.000 | 0.000 |
| SLC5A9 | 74.212 | 45.724 | -0.699 | 0.699 | 0.001 | 0.007 |
| LOC101927841 | 135.415 | 83.335 | -0.700 | 0.700 | 0.000 | 0.000 |
| SGCA | 78.549 | 48.316 | -0.701 | 0.701 | 0.000 | 0.001 |
| P2RX1 | 119.500 | 73.379 | -0.704 | 0.704 | 0.000 | 0.000 |
| NECAB1 | 26.967 | 16.548 | -0.705 | 0.705 | 0.001 | 0.005 |
| ARHGAP6 | 644.043 | 394.897 | -0.706 | 0.706 | 0.000 | 0.001 |
| JAM2 | 1274.042 | 780.041 | -0.708 | 0.708 | 0.001 | 0.004 |
| SLC46A2 | 315.503 | 192.510 | -0.713 | 0.713 | 0.009 | 0.032 |
| LOC100506725 | 106.613 | 64.951 | -0.715 | 0.715 | 0.000 | 0.000 |
| NPY1R | 167.223 | 101.563 | -0.719 | 0.719 | 0.000 | 0.002 |
| GUCY1A2 | 720.817 | 437.703 | -0.720 | 0.720 | 0.000 | 0.000 |
| RASSF9 | 364.571 | 221.358 | -0.720 | 0.720 | 0.000 | 0.000 |
| CCDC85A | 177.918 | 108.012 | -0.720 | 0.720 | 0.004 | 0.017 |
| RGS13 | 185.845 | 112.807 | -0.720 | 0.720 | 0.000 | 0.000 |
| ART4 | 219.642 | 133.235 | -0.721 | 0.721 | 0.000 | 0.000 |
| RP5-855D21.1 | 57.673 | 34.924 | -0.724 | 0.724 | 0.003 | 0.013 |
| TGFBR3 | 3692.098 | 2234.232 | -0.725 | 0.725 | 0.000 | 0.003 |
| GSTM5 | 468.772 | 283.548 | -0.725 | 0.725 | 0.000 | 0.000 |
| CLEC14A | 727.870 | 439.700 | -0.727 | 0.727 | 0.002 | 0.012 |
| FILIP1 | 615.907 | 371.535 | -0.729 | 0.729 | 0.000 | 0.000 |
| C8orf12 | 11.758 | 7.089 | -0.730 | 0.730 | 0.001 | 0.005 |
| CD5L | 38.331 | 23.110 | -0.730 | 0.730 | 0.000 | 0.002 |
| ITIH5 | 594.903 | 357.389 | -0.735 | 0.735 | 0.000 | 0.001 |
| LPL | 3143.382 | 1887.815 | -0.736 | 0.736 | 0.000 | 0.002 |
| CHRDL1 | 4547.974 | 2728.736 | -0.737 | 0.737 | 0.000 | 0.000 |
| PDZD2 | 769.319 | 460.826 | -0.739 | 0.739 | 0.000 | 0.000 |
| MACROD2 | 1098.842 | 657.437 | -0.741 | 0.741 | 0.001 | 0.008 |
| LAMP3 | 8723.155 | 5206.384 | -0.745 | 0.745 | 0.000 | 0.000 |
| ACOXL | 239.532 | 142.859 | -0.746 | 0.746 | 0.000 | 0.000 |
| ANGPT1 | 1130.978 | 672.714 | -0.750 | 0.750 | 0.000 | 0.000 |
| LRRK2 | 3554.218 | 2114.014 | -0.750 | 0.750 | 0.000 | 0.003 |
| EGR3 | 1398.450 | 831.179 | -0.751 | 0.751 | 0.015 | 0.049 |
| LINC00312 | 491.360 | 291.277 | -0.754 | 0.754 | 0.000 | 0.000 |
| STX11 | 318.323 | 188.618 | -0.755 | 0.755 | 0.000 | 0.001 |
| C2orf40 | 1543.460 | 914.234 | -0.756 | 0.756 | 0.002 | 0.011 |
| KIAA1324L | 854.155 | 505.656 | -0.756 | 0.756 | 0.000 | 0.000 |
| PTX3 | 620.044 | 366.919 | -0.757 | 0.757 | 0.002 | 0.011 |
| SLIT3 | 443.302 | 262.114 | -0.758 | 0.758 | 0.000 | 0.000 |
| FENDRR | 135.125 | 79.867 | -0.759 | 0.759 | 0.002 | 0.012 |
| ECSCR | 727.079 | 428.300 | -0.763 | 0.763 | 0.000 | 0.002 |
| LDB2 | 1546.334 | 910.593 | -0.764 | 0.764 | 0.000 | 0.002 |
| AFF3 | 121.441 | 71.512 | -0.764 | 0.764 | 0.000 | 0.000 |
| FAM13C | 865.932 | 509.133 | -0.766 | 0.766 | 0.000 | 0.000 |
| SLC51B | 65.561 | 38.450 | -0.770 | 0.770 | 0.011 | 0.037 |
| NR4A2 | 3409.237 | 1999.416 | -0.770 | 0.770 | 0.000 | 0.003 |
| TAL1 | 160.907 | 94.254 | -0.772 | 0.772 | 0.000 | 0.000 |
| FAM162B | 1280.962 | 750.202 | -0.772 | 0.772 | 0.000 | 0.001 |
| MYRIP | 266.909 | 155.454 | -0.780 | 0.780 | 0.000 | 0.002 |
| LINC00472 | 190.942 | 111.132 | -0.781 | 0.781 | 0.000 | 0.000 |
| CALCRL | 483.551 | 280.466 | -0.786 | 0.786 | 0.000 | 0.002 |
| RASIP1 | 218.169 | 126.432 | -0.787 | 0.787 | 0.001 | 0.007 |
| VEPH1 | 656.010 | 379.885 | -0.788 | 0.788 | 0.000 | 0.001 |
| TRHDE | 218.441 | 126.195 | -0.792 | 0.792 | 0.001 | 0.006 |
| LOC285043 | 433.916 | 250.667 | -0.792 | 0.792 | 0.000 | 0.001 |
| MYCT1 | 355.548 | 205.349 | -0.792 | 0.792 | 0.000 | 0.001 |
| MFAP4 | 2246.572 | 1296.692 | -0.793 | 0.793 | 0.000 | 0.001 |
| EMCN | 1182.749 | 682.561 | -0.793 | 0.793 | 0.000 | 0.003 |
| GPR146 | 248.614 | 143.432 | -0.794 | 0.794 | 0.000 | 0.002 |
| SCARA5 | 612.502 | 353.171 | -0.794 | 0.794 | 0.001 | 0.005 |
| DNASE1L3 | 223.622 | 128.788 | -0.796 | 0.796 | 0.000 | 0.002 |
| FGFR4 | 151.346 | 87.051 | -0.798 | 0.798 | 0.001 | 0.007 |
| LINC00622 | 289.058 | 166.023 | -0.800 | 0.800 | 0.000 | 0.002 |
| CDH19 | 85.897 | 49.207 | -0.804 | 0.804 | 0.010 | 0.035 |
| LINC01354 | 38.188 | 21.869 | -0.804 | 0.804 | 0.000 | 0.001 |
| CFD | 3309.218 | 1893.572 | -0.805 | 0.805 | 0.000 | 0.003 |
| ANGPTL7 | 51.669 | 29.557 | -0.806 | 0.806 | 0.005 | 0.021 |
| HBB | 5629.126 | 3215.477 | -0.808 | 0.808 | 0.001 | 0.004 |
| GDF10 | 228.871 | 130.258 | -0.813 | 0.813 | 0.000 | 0.003 |
| ADH1B | 4127.112 | 2346.117 | -0.815 | 0.815 | 0.000 | 0.000 |
| PTPRB | 939.263 | 533.517 | -0.816 | 0.816 | 0.000 | 0.001 |
| SLC19A3 | 112.550 | 63.748 | -0.820 | 0.820 | 0.000 | 0.001 |
| SEMA3G | 357.807 | 201.861 | -0.826 | 0.826 | 0.010 | 0.035 |
| CDH5 | 1434.791 | 808.512 | -0.828 | 0.828 | 0.001 | 0.008 |
| KIAA0408 | 77.904 | 43.887 | -0.828 | 0.828 | 0.000 | 0.001 |
| CSMD3 | 45.210 | 25.463 | -0.828 | 0.828 | 0.014 | 0.047 |
| LOC101929335 | 32.964 | 18.542 | -0.830 | 0.830 | 0.000 | 0.004 |
| CLDN5 | 2024.434 | 1138.054 | -0.831 | 0.831 | 0.008 | 0.029 |
| AX748273 | 263.534 | 147.870 | -0.834 | 0.834 | 0.000 | 0.000 |
| GPIHBP1 | 258.842 | 144.868 | -0.837 | 0.837 | 0.010 | 0.036 |
| SEMA6A | 199.216 | 111.494 | -0.837 | 0.837 | 0.000 | 0.002 |
| ABCA8 | 1173.918 | 656.799 | -0.838 | 0.838 | 0.000 | 0.001 |
| SVEP1 | 257.311 | 143.887 | -0.839 | 0.839 | 0.000 | 0.000 |
| DQ592442 | 580.923 | 324.650 | -0.839 | 0.839 | 0.001 | 0.005 |
| IL33 | 1727.839 | 965.110 | -0.840 | 0.840 | 0.000 | 0.000 |
| BMP6 | 140.961 | 78.641 | -0.842 | 0.842 | 0.000 | 0.000 |
| S1PR1 | 1586.877 | 884.619 | -0.843 | 0.843 | 0.000 | 0.002 |
| FGFBP2 | 162.585 | 90.632 | -0.843 | 0.843 | 0.005 | 0.020 |
| CXCL2 | 1600.765 | 891.326 | -0.845 | 0.845 | 0.000 | 0.000 |
| TMEM132C | 148.620 | 82.636 | -0.847 | 0.847 | 0.000 | 0.003 |
| CLEC4M | 40.840 | 22.680 | -0.849 | 0.849 | 0.009 | 0.034 |
| FOXF1 | 1170.613 | 646.153 | -0.857 | 0.857 | 0.003 | 0.013 |
| ADRB1 | 328.331 | 180.872 | -0.860 | 0.860 | 0.002 | 0.009 |
| NR4A1 | 668.862 | 367.572 | -0.864 | 0.864 | 0.000 | 0.001 |
| SMTNL2 | 95.589 | 52.514 | -0.864 | 0.864 | 0.002 | 0.011 |
| KCNK3 | 307.891 | 168.504 | -0.870 | 0.870 | 0.000 | 0.001 |
| LRRTM4 | 75.591 | 41.324 | -0.871 | 0.871 | 0.002 | 0.011 |
| ACADL | 488.333 | 266.071 | -0.876 | 0.876 | 0.000 | 0.001 |
| THBD | 1441.576 | 781.320 | -0.884 | 0.884 | 0.000 | 0.000 |
| SOX7 | 1742.372 | 942.865 | -0.886 | 0.886 | 0.000 | 0.002 |
| ADAMTS1 | 2643.096 | 1430.082 | -0.886 | 0.886 | 0.000 | 0.003 |
| ADAMTSL3 | 530.466 | 286.619 | -0.888 | 0.888 | 0.000 | 0.001 |
| CDO1 | 442.157 | 238.215 | -0.892 | 0.892 | 0.000 | 0.001 |
| PGM5-AS1 | 167.061 | 89.320 | -0.903 | 0.903 | 0.000 | 0.001 |
| ROBO4 | 731.989 | 390.767 | -0.906 | 0.906 | 0.000 | 0.002 |
| MAMDC2 | 2630.479 | 1403.274 | -0.907 | 0.907 | 0.000 | 0.000 |
| ASPA | 180.078 | 95.955 | -0.908 | 0.908 | 0.000 | 0.001 |
| CD300LG | 116.365 | 61.841 | -0.912 | 0.912 | 0.000 | 0.001 |
| TEX14 | 84.334 | 44.727 | -0.915 | 0.915 | 0.001 | 0.008 |
| HSPB3 | 65.522 | 34.699 | -0.917 | 0.917 | 0.000 | 0.002 |
| CCDC178 | 128.513 | 67.976 | -0.919 | 0.919 | 0.001 | 0.006 |
| KL | 274.060 | 144.332 | -0.925 | 0.925 | 0.000 | 0.000 |
| KANK3 | 419.832 | 220.752 | -0.927 | 0.927 | 0.000 | 0.000 |
| RNF182 | 354.865 | 186.539 | -0.928 | 0.928 | 0.000 | 0.000 |
| TEK | 836.398 | 438.379 | -0.932 | 0.932 | 0.000 | 0.001 |
| PEBP4 | 1002.393 | 525.168 | -0.933 | 0.933 | 0.000 | 0.002 |
| FOSB | 4563.872 | 2388.356 | -0.934 | 0.934 | 0.001 | 0.006 |
| LINC00551 | 129.634 | 67.642 | -0.938 | 0.938 | 0.009 | 0.034 |
| FAT3 | 276.175 | 144.044 | -0.939 | 0.939 | 0.001 | 0.004 |
| INMT | 2050.450 | 1066.323 | -0.943 | 0.943 | 0.000 | 0.000 |
| NCKAP5 | 474.999 | 246.375 | -0.947 | 0.947 | 0.006 | 0.023 |
| RSPO1 | 61.331 | 31.698 | -0.952 | 0.952 | 0.007 | 0.026 |
| PLCXD3 | 118.730 | 60.762 | -0.966 | 0.966 | 0.000 | 0.001 |
| ADCY8 | 24.392 | 12.359 | -0.981 | 0.981 | 0.000 | 0.003 |
| RP1-78O14.1 | 316.113 | 159.892 | -0.983 | 0.983 | 0.002 | 0.009 |
| DACH1 | 495.586 | 250.456 | -0.985 | 0.985 | 0.000 | 0.001 |
| GKN2 | 607.008 | 306.509 | -0.986 | 0.986 | 0.015 | 0.049 |
| PTPN5 | 43.198 | 21.796 | -0.987 | 0.987 | 0.001 | 0.006 |
| RSPO2 | 88.313 | 44.470 | -0.990 | 0.990 | 0.000 | 0.000 |
| AOC3 | 5106.252 | 2570.678 | -0.990 | 0.990 | 0.000 | 0.000 |
| ANGPTL1 | 319.383 | 160.583 | -0.992 | 0.992 | 0.000 | 0.002 |
| TCF21 | 944.565 | 474.261 | -0.994 | 0.994 | 0.000 | 0.001 |
| SLC10A4 | 169.481 | 85.062 | -0.995 | 0.995 | 0.006 | 0.023 |
| FHL5 | 215.849 | 108.090 | -0.998 | 0.998 | 0.000 | 0.000 |
| TSPAN7 | 1999.101 | 991.756 | -1.011 | 1.011 | 0.000 | 0.000 |
| TMEM100 | 3291.763 | 1632.218 | -1.012 | 1.012 | 0.005 | 0.021 |
| VIPR1 | 429.156 | 212.502 | -1.014 | 1.014 | 0.001 | 0.005 |
| TNNC1 | 206.895 | 102.393 | -1.015 | 1.015 | 0.001 | 0.005 |
| KCTD16 | 75.257 | 37.019 | -1.024 | 1.024 | 0.000 | 0.000 |
| BRE-AS1 | 233.106 | 114.382 | -1.027 | 1.027 | 0.000 | 0.003 |
| SDPR | 2234.264 | 1093.858 | -1.030 | 1.030 | 0.000 | 0.000 |
| LINC01314 | 85.433 | 41.545 | -1.040 | 1.040 | 0.000 | 0.002 |
| EDNRB | 1580.621 | 765.557 | -1.046 | 1.046 | 0.000 | 0.001 |
| LHFPL3-AS2 | 710.213 | 343.198 | -1.049 | 1.049 | 0.001 | 0.004 |
| UGT2B4 | 83.843 | 40.265 | -1.058 | 1.058 | 0.000 | 0.001 |
| NR4A3 | 711.680 | 340.448 | -1.064 | 1.064 | 0.000 | 0.002 |
| RP11-476D10.1 | 395.822 | 189.137 | -1.065 | 1.065 | 0.007 | 0.027 |
| PKHD1L1 | 90.229 | 43.058 | -1.067 | 1.067 | 0.000 | 0.000 |
| CHIAP2 | 35.580 | 16.924 | -1.072 | 1.072 | 0.015 | 0.048 |
| SCN4B | 655.504 | 311.774 | -1.072 | 1.072 | 0.000 | 0.000 |
| STXBP6 | 350.144 | 166.534 | -1.072 | 1.072 | 0.004 | 0.016 |
| ZBTB16 | 981.935 | 464.152 | -1.081 | 1.081 | 0.000 | 0.000 |
| LINC00968 | 440.888 | 208.357 | -1.081 | 1.081 | 0.000 | 0.000 |
| RAMP3 | 435.481 | 204.529 | -1.090 | 1.090 | 0.008 | 0.029 |
| USHBP1 | 41.183 | 19.287 | -1.094 | 1.094 | 0.001 | 0.005 |
| CCBE1 | 345.955 | 159.848 | -1.114 | 1.114 | 0.000 | 0.000 |
| MMRN1 | 465.603 | 214.490 | -1.118 | 1.118 | 0.000 | 0.001 |
| LRRN3 | 774.751 | 355.265 | -1.125 | 1.125 | 0.000 | 0.000 |
| RXFP1 | 136.279 | 62.460 | -1.126 | 1.126 | 0.000 | 0.001 |
| ANKRD29 | 1138.401 | 518.347 | -1.135 | 1.135 | 0.000 | 0.000 |
| FAM107A | 705.183 | 318.540 | -1.147 | 1.147 | 0.000 | 0.002 |
| RELN | 703.469 | 315.943 | -1.155 | 1.155 | 0.003 | 0.013 |
| CD36 | 992.965 | 438.336 | -1.180 | 1.180 | 0.000 | 0.000 |
| BTNL9 | 532.435 | 231.624 | -1.201 | 1.201 | 0.001 | 0.005 |
| IGSF10 | 758.441 | 329.726 | -1.202 | 1.202 | 0.000 | 0.002 |
| BMX | 185.147 | 80.166 | -1.208 | 1.208 | 0.000 | 0.001 |
| COL6A6 | 891.640 | 375.498 | -1.248 | 1.248 | 0.000 | 0.000 |
| FAM150B | 503.168 | 207.609 | -1.277 | 1.277 | 0.000 | 0.001 |
| GRIA1 | 168.233 | 68.769 | -1.291 | 1.291 | 0.000 | 0.000 |
| LYVE1 | 256.224 | 103.605 | -1.306 | 1.306 | 0.000 | 0.001 |
| LOC400568 | 43.559 | 17.566 | -1.310 | 1.310 | 0.001 | 0.006 |
| TENM2 | 329.946 | 131.725 | -1.325 | 1.325 | 0.015 | 0.048 |
| FLJ35700 | 254.412 | 101.006 | -1.333 | 1.333 | 0.000 | 0.001 |
| SELE | 1003.394 | 389.639 | -1.365 | 1.365 | 0.003 | 0.013 |
| GPM6A | 647.168 | 246.277 | -1.394 | 1.394 | 0.000 | 0.001 |
| SOSTDC1 | 521.226 | 192.939 | -1.434 | 1.434 | 0.007 | 0.026 |
| CA4 | 244.978 | 88.220 | -1.473 | 1.473 | 0.005 | 0.021 |
| IL6 | 1161.078 | 406.222 | -1.515 | 1.515 | 0.000 | 0.001 |
| ERVFRD-1 | 14.451 | 4.947 | -1.546 | 1.546 | 0.008 | 0.029 |
| FABP4 | 592.585 | 202.503 | -1.549 | 1.549 | 0.000 | 0.001 |
| AGER | 1196.330 | 400.610 | -1.578 | 1.578 | 0.007 | 0.027 |
| SGCG | 208.585 | 68.334 | -1.610 | 1.610 | 0.001 | 0.004 |
| FLJ34503 | 50.982 | 16.075 | -1.665 | 1.665 | 0.001 | 0.007 |
| CA3 | 545.383 | 162.711 | -1.745 | 1.745 | 0.001 | 0.005 |
| EMX2 | 25.659 | 7.538 | -1.767 | 1.767 | 0.002 | 0.009 |
| SLC6A4 | 583.391 | 144.676 | -2.012 | 2.012 | 0.000 | 0.004 |
| LGI3 | 59.623 | 14.208 | -2.069 | 2.069 | 0.000 | 0.000 |
| RP11-116O18.1 | 118.353 | 25.673 | -2.205 | 2.205 | 0.000 | 0.002 |
| SLC10A2 | 288.448 | 60.700 | -2.249 | 2.249 | 0.014 | 0.046 |


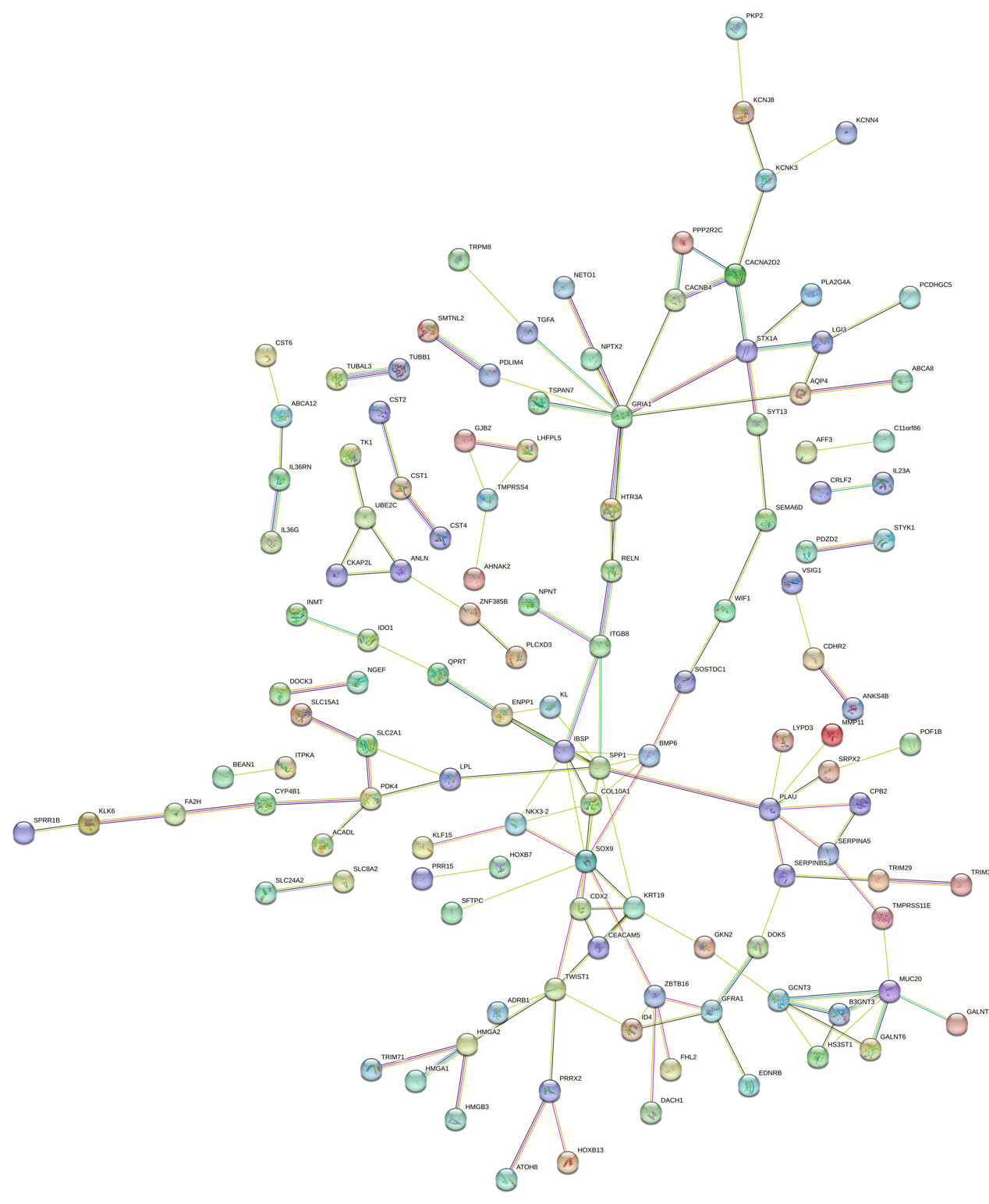


Figure S1. A protein-protein interaction network


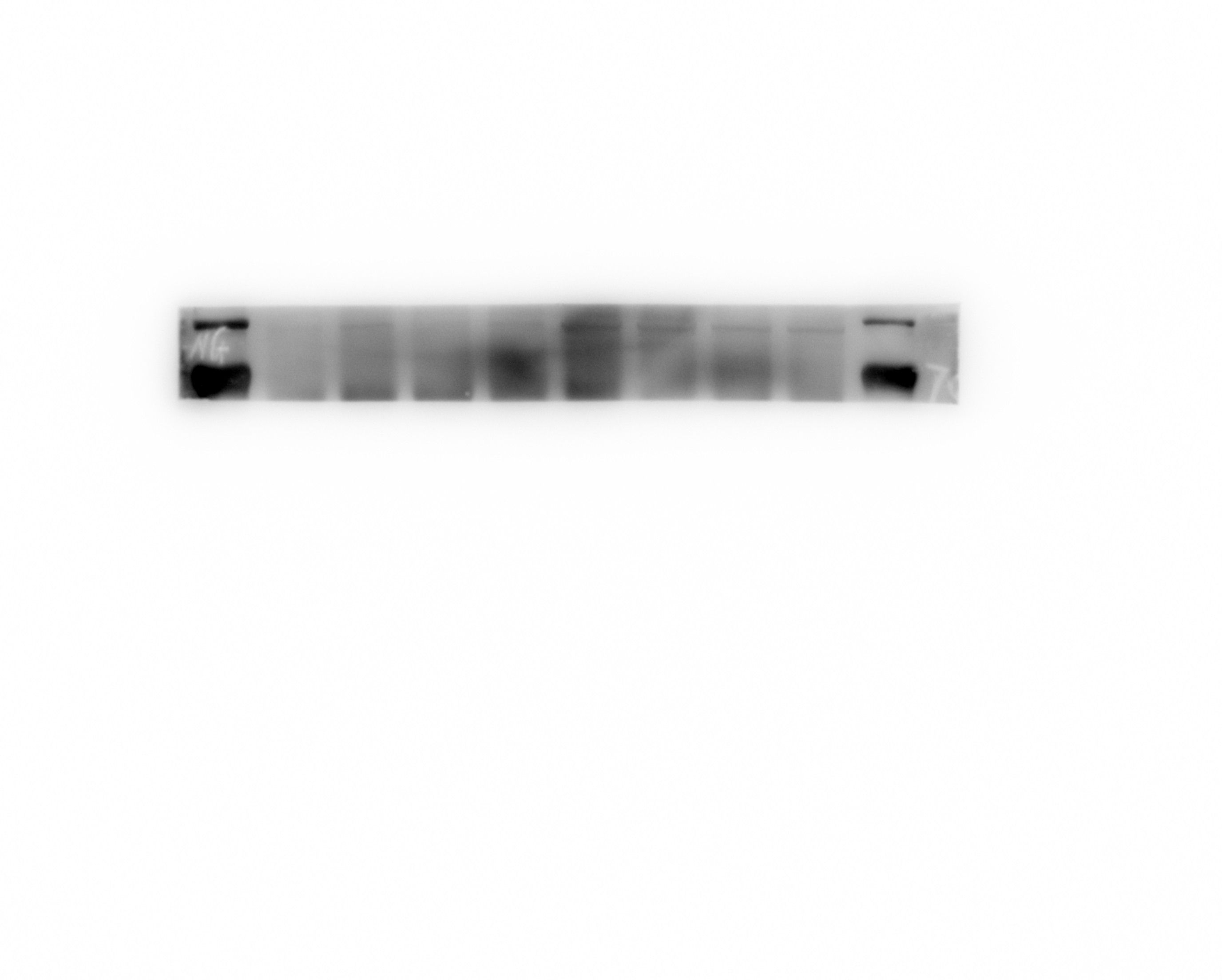


The raw western blot membranes of NGEF.


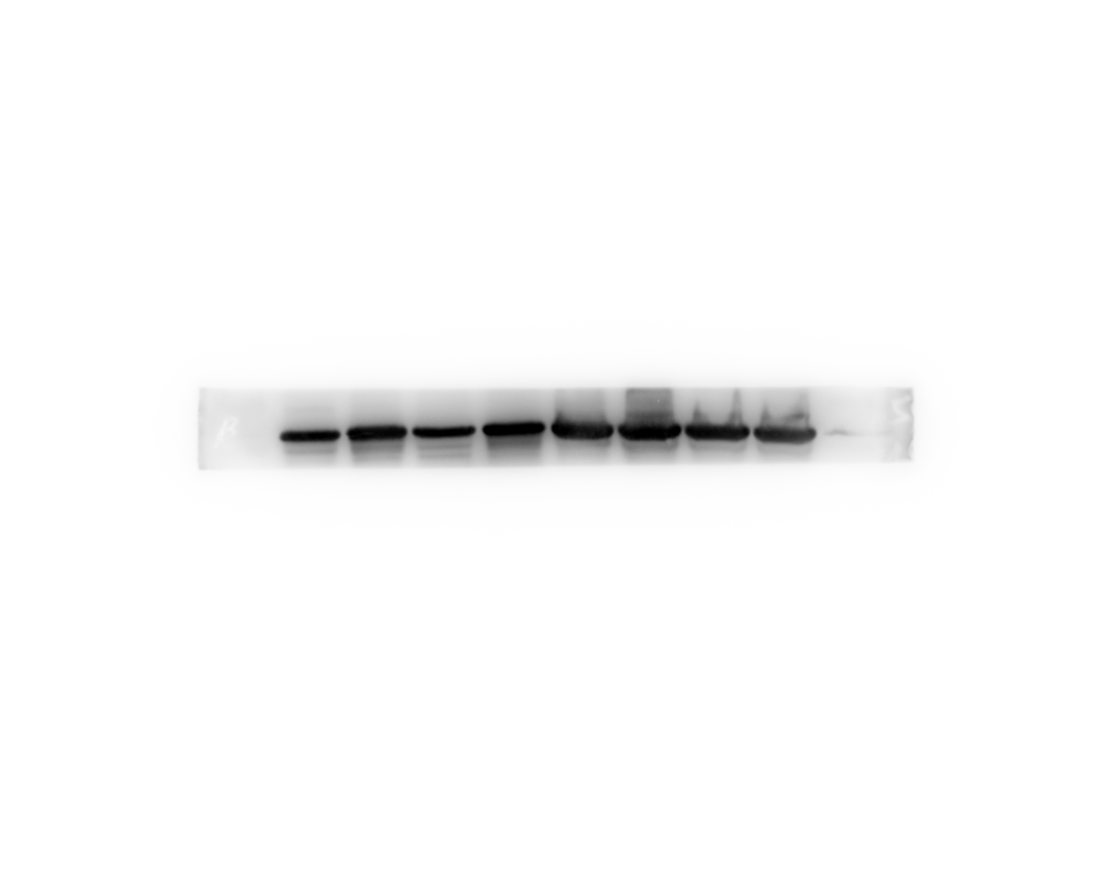


The raw western blot membranes of Beta-actin.
